# Quantitative and Molecular Genetic Analyses of Mutations Increasing *Drosophila* Life Span

**DOI:** 10.1371/journal.pgen.1001037

**Published:** 2010-07-29

**Authors:** Michael M. Magwire, Akihiko Yamamoto, Mary Anna Carbone, Natalia V. Roshina, Alexander V. Symonenko, Elena G. Pasyukova, Tatiana V. Morozova, Trudy F. C. Mackay

**Affiliations:** 1 Department of Genetics and W. M. Keck Center for Behavioral Biology, North Carolina State University, Raleigh, North Carolina, United States of America; 2 Institute of Molecular Genetics of the Russian Academy of Sciences, Moscow, Russia; Fred Hutchinson Cancer Research Center, United States of America

## Abstract

Understanding the genetic and environmental factors that affect variation in life span and senescence is of major interest for human health and evolutionary biology. Multiple mechanisms affect longevity, many of which are conserved across species, but the genetic networks underlying each mechanism and cross-talk between networks are unknown. We report the results of a screen for mutations affecting *Drosophila* life span. One third of the 1,332 homozygous *P*–element insertion lines assessed had quantitative effects on life span; mutations reducing life span were twice as common as mutations increasing life span. We confirmed 58 mutations with increased longevity, only one of which is in a gene previously associated with life span. The effects of the mutations increasing life span were highly sex-specific, with a trend towards opposite effects in males and females. Mutations in the same gene were associated with both increased and decreased life span, depending on the location and orientation of the *P*–element insertion, and genetic background. We observed substantial—and sex-specific—epistasis among a sample of ten mutations with increased life span. All mutations increasing life span had at least one deleterious pleiotropic effect on stress resistance or general health, with different patterns of pleiotropy for males and females. Whole-genome transcript profiles of seven of the mutant lines and the wild type revealed 4,488 differentially expressed transcripts, 553 of which were common to four or more of the mutant lines, which include genes previously associated with life span and novel genes implicated by this study. Therefore longevity has a large mutational target size; genes affecting life span have variable allelic effects; alleles affecting life span exhibit antagonistic pleiotropy and form epistatic networks; and sex-specific mutational effects are ubiquitous. Comparison of transcript profiles of long-lived mutations and the control line reveals a transcriptional signature of increased life span.

## Author Summary

Recent advances in medical science as well as vastly improved living conditions have resulted in a steady increase in human life span, with a concomitant increase in health issues associated with aging. In addition, understanding life history evolution requires that we know why organisms age and why there is variation in aging and senescence. To identify genes involved in aging, we assessed longevity in a collection of over 1,300 *Drosophila* lines homozygous for a single *P* transposable element mutation. We found 58 mutations in novel loci that increase life span by up to 33%. Most mutations had different effects on male and female life span, and for some the effects were opposite between the sexes. Effects of these mutations on starvation resistance, chill coma recovery, and climbing ability varied, but all had a deleterious effect on at least one other trait. A sample of ten mutations with increased life span formed genetic interaction networks, but the genetic interactions were different, and sometimes in opposite directions, in males and females. Transcript profiles of seven long-lived mutations and the control line reveal a core transcriptional signature of increased life span involving novel candidate genes for future analysis.

## Introduction

Understanding the genetic and environmental factors affecting variation in life span and health span is of major interest for human health and evolutionary biology. As the world population ages, the incidence of age-related diseases, such as Alzheimer's disease, cancer, cardiovascular disease and Huntington's disease, is concomitantly increasing. From the evolutionary perspective, we seek to understand why aging occurs, and why there is variation in aging between and within species [1], [2].

Multiple mechanisms affecting longevity have been documented, many of which are conserved across species. Dietary restriction [3]–[6], oxidative stress [7]–[8] and insulin/IGF signaling (IIS) [9]–[17] all affect longevity. Additional processes that change with age include stress response [18], [19], telomere shortening [20] and gene silencing [21], [22]. Life span extension is often accompanied by a decline in reproduction [23]–[27], a well–known trade–off that could explain limits to life span and maintenance of genetic variation for longevity within species [28]–[30]. However, this relationship is not universal [31]–[34]. Similarly, positive correlations between life span and stress resistance [18] are not always observed [35].

Given the heterogeneity of mechanisms affecting life span and the need to understand the genetic networks underlying each mechanism as well as cross–talk between networks, there is a clear need for unbiased, genome–wide screens to identify genes and genetic networks affecting life span. Studies using microarray technology to observe changes in gene expression during normal aging or following exposure to conditions that extend or reduce life span have indeed confirmed that expression of a substantial fraction of the genome changes with age [36]–[44]. However, these analyses are correlative, and cannot distinguish between changes in gene expression that cause aging from changes in gene expression that are a consequence of aging.

Genetic screens for mutations affecting life span give unambiguous insight regarding the genes and pathways required for normal aging, as elegantly demonstrated by mutagenesis and RNAi screens in the short-lived model organism, *C. elegans* [45]–[49]. Genetic screens for mutations affecting life span are more laborious in longer-lived species, such as *Drosophila*, and consequently there have been relatively few studies reporting mutations increasing life span in this organism [50]–[54]. Here, we report the results of a screen for mutations affecting *Drosophila* life span, utilizing a collection of over 1,000 single, homozygous *P*–element insertion lines that were constructed in isogenic backgrounds [55]. We identified 58 mutations with increased longevity, only one of which is in a gene previously associated with life span. The effects of the mutations are highly sex–specific, with life span extensions ranging from 5%–33%. The mutations have pleiotropic effects on resistance to starvation stress, chill coma recovery time, and locomotion, but the pleiotropic effects are highly variable. All of the mutations associated with increased life span have at least one deleterious pleiotropic effect on stress resistance or general health, indicating the complicated mutational basis of trade–offs between putative fitness components. We performed a quantitative genetic analysis of epistasis [56]–[58] among ten of these mutations to derive genetic interaction networks, and found that epistasis is pervasive and sex–specific. Finally, we obtained whole genome transcript profiles of seven of the mutant lines and the wild type control to evaluate the biological impact of the mutant alleles [59]–[61] and derive a common transcriptional signature of increased life span.

## Results

### Screen for mutations affecting life span

To identify mutations affecting *Drosophila* life span, we quantified the life span of males and females of 1,332 homozygous *P{GT1}* insertion lines [55], [57], [58], [62], [63] simultaneously with their co–isogenic control lines (Table S1). Analysis of variance (ANOVA, Table 1) revealed significant variation in life span among the *P*–element insert lines (*P*<0.0001) as well as significant sex–specific effects on life span (*P*<0.0001). Our estimates of the broad–sense mutational heritability (*H_M_* ^2^) and the cross–sex mutational genetic correlation (*r_FM_*±SE) of life span were *H_M_* ^2^ = 0.557 and *r_FM_* = 0.555±0.025. Averaged over all mutations, the standardized effects (*a/σ_P_* [64]) of the *P*–element insertions on life span were slightly negative, with *a/σ_P_* = −0.41 in females and *a/σ_P_* = −0.45 in males.

**Table 1:** Analyses of variance of life span of 1,332 *P{GT1}* insertion lines.

Analysis	Source	d.f.	MS	*F*	*P*	*σ* ^2^ a
Sexes pooled	Sex	1	274.89	6.11	0.0135	—
	Line	1331	241.89	5.37	<0.0001	31.39
	LinexSex	1312	101.56	2.26	<0.0001	25.24
	Error	3301	45.02	—	—	45.02
Males	Line	1321	164.50	4.75	<0.0001	57.93
	Error	1643	34.60	—	—	34.60
Females	Line	1322	180.11	3.25	<0.0001	55.37
	Error	1658	55.36	—	—	55.36

**^a^**Variance component.

**Figure 1:**
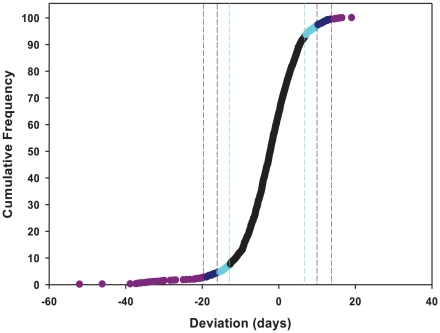
Cumulative frequency distribution of effects of mutations on life span. Life spans are averaged across sexes, and expressed as deviations from the mean of co–isogenic controls. Lines with mean life spans exceeding the 95%, 99% and 99.9% confidence intervals are depicted in cyan, dark blue and dark pink, respectively.

To identify the individual *P*–element insert lines that contributed to the significant variation in life span, we computed the confidence intervals (CIs) of deviations of line means from their corresponding controls (Figure 1), and performed Dunnett's *t*–tests to assess deviations of insert lines from the control line within each experimental block. Combining the results of both analyses, we identified 296 lines associated with reduced life span, 135 with increased life span, and 5 with sexually antagonistic effects on life span. At the 95%, 99% and 99.9% CIs, respectively, 139 (194), 55 (95) and 12 (49) lines had significantly increased (decreased) life span in at least one sex or averaged across sexes (Table S1). The Dunnett's tests indicated 70 (270) lines had increased (decreased) life span (*P*<0.05, after correction for multiple tests) (Table S1). Both analyses indicate an asymmetrical distribution of mutational effects, with more mutations decreasing than increasing longevity, as expected for components of fitness. It is generally assumed that mutations decreasing life span are less interesting than mutations increasing life span, since the former category of mutations could be generally deleterious and affect all aspects of fitness, while the latter are more likely to have specific effects on life span. Thus, we concentrated on confirming the effects of mutations associated with increased life span. We chose 83 mutations with increased life span and re-assessed their life span using larger sample sizes. We found that 58 of the 83 mutations (70%) remained formally significant for at least one sex, and 43 lines had effects that were significant following a Bonferroni correction for multiple testing (*P*≤0.0006) (Table 2). Thus, 4.4% of the mutations we screened are associated with increased longevity. This indicates a large mutational target for longevity and extensive pleiotropy among genes affecting life span.

**Table 2:** Effects of *P{GT1}* insertion lines with increased life span.

Line	Mutational Effects						*P*–values from ANOVA					Category
	Life Span (% a)			*a/σ_p_* b			Sexes Pooled c			Sexes Separate		
	♂,♀	♂	♀	♂,♀	♂	♀	*S*	*L*	*L*×*S*	*L*♂	*L*♀	
**BG00004 (F)**	60.1 (9.0)	61.7 (16.6)	58.4 (2.3)	0.18	0.36	0.04	ns	*	ns	**	ns	both sexes
BG00008 (F)	60.6 (9.8)	64.1 (21.1)	57.1 (0.1)	0.20	0.46	0.00	ns	***	**	****	ns	male–specific
**BG00010 (F)**	63.1 (14.4)	64.6 (22.1)	61.6 (8.0)	0.29	0.48	0.15	ns	****	ns	****	ns	both sexes
**BG00028 (F)**	61.8 (12.1)	67.7 (27.8)	56.4 (−1.2)	0.24	0.61	−0.02	*	****	****	****	ns	male–specific
BG00037 (F)	60.6 (10.0)	54.6 (3.3)	66.5 (16.5)	0.20	0.07	0.32	****	***	*	ns	****	female–specific
BG00039 (F)	59.5 (8.0)	52.6 (−0.5)	66.3 (16.2)	0.16	−0.01	0.31	****	*	**	ns	***	female–specific
BG00041 (F)	57.2 (3.7)	60.7 (14.7)	53.7 (−5.8)	0.07	0.32	−0.11	ns	ns	**	***	ns	male–specific
BG00042 (F)	64.1 (16.3)	70.6 (33.4)	57.8 (1.2)	0.33	0.73	0.02	*	****	***	****	ns	male–specific
**BG00043 (F)**	66.9 (21.4)	67.3 (27.2)	66.6 (16.7)	0.43	0.60	0.32	ns	****	ns	****	****	both sexes
BG00080 (B)	63.1 (8.3)	58.3 (7.7)	66.7 (6.9)	0.24	0.18	0.33	****	*	ns	ns	**	both sexes
BG00106 (F)	65.3 (19.8)	65.2 (25.6)	65.4 (14.6)	0.40	0.68	0.26	ns	****	ns	****	*	both sexes
BG00121 (F)	68.3 (3.7)	68.0 (9.3)	68.6 (−1.2)	0.12	0.27	−0.05	**	*	**	**	ns	male–specific
BG00218 (F)	61.7 (12.6)	61.8 (22.8)	61.6 (4.6)	0.32	0.65	0.13	****	****	****	****	*	male–biased
**BG00297 (F)**	62.9 (14.0)	65.5 (23.8)	60.3 (5.6)	0.28	0.52	0.11	ns	****	**	****	ns	male–specific
BG00336 (B)	66.7 (14.6)	64.2 (18.6)	69.3 (11.0)	0.42	0.44	0.54	****	****	ns	****	***	both sexes
**BG00346 (F)**	66.7 (12.8)	67.4 (26.8)	65.8 (1.5)	0.27	0.57	0.04	***	****	****	****	ns	male–specific
BG00472 (F)	60.5 (9.7)	62.5 (18.0)	58.5 (2.4)	0.19	0.40	0.05	ns	**	*	****	ns	male–specific
**BG00495 (F)**	70.2 (27.3)	70.2 (32.7)	70.1 (22.9)	0.55	0.72	0.44	ns	****	ns	****	****	both sexes
BG00528 (B)	67.4 (11.1)	68.0 (0.6)	66.9 (22.8)	0.24	0.01	0.63	****	****	***	ns	****	female–specific
BG00719 (F)	65.4 (−0.7)	65.6 (5.5)	65.1 (−6.2)	−0.02	0.16	−0.25	**	ns	**	*	*	sex–antagonistic
BG00757 (F)	60.7 (10.2)	67.1 (26.8)	54.5 (−4.5)	0.20	0.59	−0.09	*	**	****	****	ns	male–specific
**BG00761 (F)**	63.1 (14.5)	60.3 (14.0)	65.8 (15.3)	0.29	0.31	0.29	**	****	ns	**	****	both sexes
BG00767 (B)	69.2 (13.9)	71.5 (5.8)	66.7 (22.6)	0.30	0.13	0.62	****	****	**	*	****	female–biased
**BG00817 (F)**	69.2 (5.1)	70.1 (12.7)	68.3 (−1.7)	0.16	0.37	−0.07	*	*	**	***	ns	male–specific
BG00864 (B)	59.9 (−1.3)	57.5 (−14.9)	62.3 (14.4)	−0.03	−0.35	0.40	*	ns	****	****	**	sex–antagonistic
BG00890 (F)	60.9 (10.4)	63.0 (19.1)	58.8 (3.0)	0.21	0.42	0.06	ns	**	*	***	ns	male–specific
BG00907 (F)	61.4 (12.5)	65.8 (26.7)	57.4 (0.6)	0.25	0.71	0.01	ns	***	***	****	ns	male–specific
BG00915 (F)	64.0 (16.1)	66.7 (26.0)	61.3 (7.4)	0.32	0.57	0.14	ns	****	*	****	ns	male–specific
BG01004 (F)	59.6 (9.3)	61.3 (18.0)	58.0 (1.5)	0.19	0.48	0.03	ns	**	*	****	ns	male–specific
BG01030 (A)	62.7 (3.0)	64.8 (5.3)	74.8 (27.7)	0.08	0.15	0.64	*	****	****	ns	****	female–specific
BG01031 (A)	69.8 (16.3)	69.1 (13.3)	55.6 (−8.4)	0.35	0.34	−0.16	**	ns	**	**	ns	male–specific
**BG01042 (F)**	57.1 (3.9)	61.0 (21.1)	53.4 (−9.8)	0.10	0.55	−0.29	ns	ns	****	****	**	sex–antagonistic
BG01085 (F)	60.9 (11.7)	62.3 (20.1)	59.4 (4.1)	0.24	0.53	0.07	ns	**	*	****	ns	male–specific
BG01121 (F)	63.5 (0.6)	66.3 (6.6)	60.6 (−5.0)	0.02	0.23	−0.13	ns	ns	*	**	ns	male–specific
BG01283 (F)	61.7 (12.3)	60.5 (20.2)	62.8 (6.1)	0.32	0.53	0.18	**	****	*	****	ns	male–specific
BG01345 (A)	72.9 (21.3)	74.8 (21.5)	70.8 (21.0)	0.45	0.55	0.38	*	****	ns	****	****	both sexes
BG01403 (A)	68.2 (13.6)	65.9 (7.1)	70.5 (20.4)	0.29	0.18	0.37	ns	****	*	*	****	female–biased
BG01540 (B)	67.3 (4.6)	60.5 (−5.3)	73.5 (13.4)	0.14	−0.16	0.46	****	ns	****	ns	****	female–specific
BG01550 (F)	61.2 (−3.0)	66.1 (6.2)	56.4 (−11.6)	−0.09	0.21	−0.30	ns	ns	**	*	ns†	sex–antagonistic
BG01551 (F)	66.4 (5.2)	68.4 (10.0)	64.2 (0.6)	0.15	0.35	0.01	ns	*	ns	**	ns	both sexes
BG01553 (F)	59.9 (8.9)	58.8 (16.7)	61.0 (3.0)	0.23	0.44	0.09	****	***	*	****	ns	male–specific
BG01615 (A)	70.3 (17.0)	75.2 (22.0)	65.5 (11.8)	0.36	0.57	0.22	**	****	ns	****	*	both sexes
BG01677 (A)	71.7 (19.4)	72.0 (16.9)	71.4 (21.9)	0.41	0.44	0.40	ns	****	ns	****	****	both sexes
BG01700 (F)	70.6 (11.9)	73.2 (17.6)	68.2 (6.9)	0.35	0.61	0.18	ns	****	*	****	*	male–biased
BG01701 (F)	70.0 (11.0)	65.8 (5.8)	74.0 (15.8)	0.32	0.20	0.41	ns	*	ns	*	ns†	both sexes
BG01702 (A)	61.4 (1.0)	66.0 (8.2)	56.5 (−7.0)	0.03	0.24	−0.17	**	ns	**	*	ns	male–specific
BG01710 (A)	64.1 (5.3)	62.9 (3.2)	65.1 (7.2)	0.14	0.09	0.17	ns	*	ns	ns	*	both sexes
BG01878 (B)	65.7 (8.3)	63.5 (−6.1)	68.0 (24.9)	0.18	−0.14	0.69	**	***	****	ns	****	female–specific
BG01918 (A)	63.8 (4.8)	66.5 (9.0)	61.3 (1.0)	0.13	0.26	0.02	ns	ns†	ns	*	ns	male–specific
BG01950 (B)	52.7 (−13.1)	47.5 (−29.8)	63.1 (15.9)	−0.29	−0.69	0.44	ns	***	****	****	****	sex–antagonistic
BG01976 (B)	64.5 (6.2)	61.4 (−9.2)	67.5 (24.0)	0.13	−0.21	0.66	*	*	****	*	****	sex–antagonistic
BG02019 (B)	62.5 (3.0)	63.1 (−6.7)	62.0 (14.0)	0.07	−0.16	0.39	****	ns	****	ns	****	female–specific
BG02039 (A)	66.3 (9.0)	67.5 (10.7)	65.2 (7.3)	0.23	0.31	0.18	ns	***	ns	**	ns	both sexes
BG02049 (B)	68.1 (12.2)	71.3 (5.5)	65.1 (19.6)	0.27	0.13	0.54	****	****	*	ns	****	female–specific
BG02128 (B)	63.8 (5.0)	67.2 (−0.7)	60.2 (10.7)	0.11	−0.02	0.30	****	ns	*	ns	***	female–specific
BG02257 (B)	63.5 (8.9)	63.7 (10.3)	63.3 (7.4)	0.25	0.30	0.20	ns	****	ns	****	**	both sexes
BG02395 (B)	62.8 (8.0)	59.7 (10.4)	65.3 (4.6)	0.23	0.24	0.23	****	**	ns	*	ns	both sexes
BG02644 (B)	61.3 (5.2)	61.1 (12.9)	61.4 (−1.5)	0.15	0.30	−0.07	***	*	***	***	ns	male–specific

Candidate genes used for analysis of epistasis are shown in bold font. Letters in parenthesis after the Line name denote different co-isogenic *Canton S* host strains for *P{GT1}*–element insertion.

**^a^**Percent deviation from the mean life span of the control line.

**^b^**Standardized mutational effect. *a* = one half of the difference in life span between the homozygous mutant and control line, *σ_P_* = the phenotypic standard deviation of the control.

**^c^** *S* and *L* denote the main cross-classified effects of Sex and Line, respectively in the ANOVA of life span. ns *P*>0.1, † 0.05<*P*<0.1, * *P*<0.05, ** *P*<0.01, *** *P*<0.001, **** *P*<0.0001. Bold and unlined asterisks denote *P*–values that are significant after Bonferroni correction for multiple tests.

### Mutational effects on life span

We quantified the magnitude of the mutational effects on life span for the 58 mutations with increased life span in terms of percentage increase over the control strain, and by computing their standardized mutational effects, *a/σ_P_* [64] (Table 2). The effects of the mutations on life span correspond to an average change in longevity relative to the control of 12% pooled across sexes, 17% in males and 15% in females. The average absolute value of *a/σ_P_* is 0.27 pooled across sexes, 0.43 in males and 0.39 in females. Thus the average effects of *P*–element insertions on longevity, although statistically significant, are subtle, but effects range from 5% to as large as 33%.

We observed substantial variation in sexual dimorphism for the effects of *P{GT1}*–element insertions on longevity, as indicated by significant line by sex interaction terms in the ANOVAs pooled across sexes (Table 2). The cross–sex mutational genetic correlation for longevity among the 58 long-lived mutant lines was negative and significantly different from zero (*r_FM_* = −0.295±0.128, *t*_56_ = 2.308, *P*<0.05). Mutations associated with an increase in longevity have highly sex–specific effects, with a trend towards opposite effects in males and females.

We used the pattern of significance of the line (*L*) and line by sex (*L*×*S*) terms from ANOVAs comparing the life span of each long-lived mutant line to the control to infer whether the mutations affected both sexes, or were sex–specific, sex-biased, or sex antagonistic (Table 2). Mutations in 17 lines affected both sexes (the *L* term was significant, but the *L*×*S* term was not significant). The remaining 41 mutations (70.7%) affected males and females differently. We categorized the mutational effects as “sex–specific” if the *L*×*S* interaction from the analysis pooled across sexes was significant, and the *L* term from the separate sex analysis was significant only in one sex; “sex-biased” if the *L* and *L*×*S* terms from the analysis pooled across sexes were both significant, and the *L* term from the separate sex analysis was significant in both sexes; and “sex–antagonistic” if the *L* term from the analysis pooled across sexes was not significant, but the *L*×*S* interaction was significant, and the *L* term from the separate sex analysis was significant in both sexes. We found 22 male–specific, two male-biased, nine female–specific, two female-biased, and six sex–antagonistic mutations (Table 2).

### Candidate genes

To identify candidate genes affecting life span, we mapped the sequences flanking the *P*–element insertion sites to the reference genome (Table S1, Table 3). The flanking sequences of 47 of the *P*–element mutations associated with increased life span mapped to unique insertion sites (Table 3). Eight of the *P*–element insertions were ≥2 kb from the nearest annotated gene, and either have long–range effects on the nearest neighboring gene(s) or affect an un-annotated gene in the more immediate vicinity. The remaining 39 *P*–element inserts were <2 kb from the nearest gene. Of these, 27 were adjacent to or within the predicted transcript of the only gene in the region, and most parsimoniously affect these genes. A total of 13 inserts were located in an intergenic region, <2 kb from each flanking gene, and could affect either or both adjacent genes.

**Table 3:** Candidate genes for *P{GT1}* insertions associated with increased life span.

Line	Cytological Location	Nearest Gene(s)	*P{GT1}* Insertion SiteRelative to Gene(s)	Gene Ontology	
				Molecular Function	Biological Process
**BG00004 (F)**	32F3	No gene in region			
BG00008 (F)	85B7	*polychaetoid*	630 bp upstream	guanylate kinase activity	cell–cell adhesion; fusion cell fate specification; branch fusion, open tracheal system
**BG00010 (F)**	82D1	*CG31531*	170 bp upstream	Unknown	Unknown
**BG00028 (F)**	85B7	*polychaetoid*	630 bp upstream	See BG00008	See BG00008
BG00037 (F)	70E2	*CG9238*	230 bp upstream	protein phosphatase type 1 regulator activity; protein phosphatase 1 binding	Unknown
BG00039 (F)	79A2	*mushroom body expressed*	1.1 kb into gene (1st intron)	poly(C) RNA binding	regulation of alternative nuclear mRNA splicing, via spliceosome
BG00041 (F)	79A2	*mushroom body expressed*	1 kb into gene (1st intron)	See BG00039	See BG00039
BG00042 (F)	79A2	*mushroom body expressed*	1 kb into gene (1st intron)	See BG00039	See BG00039
**BG00043 (F)**	79A2	*mushroom body expressed*	1 kb into gene (1st intron)	See BG00039	See BG00039
BG00080 (B)	82E6	No gene in region			
BG00106 (F)	79A2	*mushroom body expressed*	1 kb into gene (1st intron)	See BG00039	See BG00039
BG00121 (F)	77E–F	No sequence			
BG00218 (F)	5A12	*Trapped in endoderm 1*/*Gustatory receptor 5a*	Adjacent *Trapped in endoderm 1*/750 bp upstream *Gustatory receptor 5a*	taste receptor activity	sensory perception of sweet taste; response to trehalose stimulus/germ cell migration; germ cell development
**BG00297 (F)**	75B7	No gene in region			
BG00336 (B)	64B13	*CG18418*/*Guanine nucleotide exchange factor 64C*	1.6 kb upstream *CG18418*/1.1 kb upstream *Guanine nucleotide exchange factor 64C*	oxoglutarate∶malate antiporter activity; transmembrane transporter activity/guanyl–nucleotide exchange factor activity	mitochondrial transport; malate transport; alpha–ketoglutarate transport/inter–male aggressive behavior; axon guidance; spiracle morphogenesis, open tracheal system
**BG00346 (F)**	33A2	*crooked legs*/*CG14939*	60 bp into gene (1st exon) *crooked legs*/700 bp downstream from *CG14939*	RNA polymerase II transcription factor activity/Unknown	negative regulation of transcription; cell adhesion; negative regulation of Wnt receptor signaling pathway; regulation of transcription from RNA polymerase II promoter; imaginal disc-derived wing morphogenesis; positive regulation of mitotic cell cycle/Unknown
BG00472 (F)	6D8	No gene in region			
**BG00495 (F)**	12B4	*CG10990*	8 kb into gene (3rd intron)	Unknown	Unknown
BG00528 (B)	83E2	*Osiris 9*	150 bp upstream	Unknown	Unknown
BG00719 (F)	Unknown	No sequence			
BG00757 (F)	Unknown	No sequence			
**BG00761 (F)**	70E2	*CG9238*	200 bp upstream	**See** BG00037	**See** BG00037
BG00767 (B)	64B13	*CG18418*/*Guanine nucleotide exchange factor 64C*	1.6 kb upstream *CG18418*/1.1 kb upstream *Guanine nucleotide exchange factor 64C*	See BG00336	See BG00336
**BG00817 (F)**	88A	No sequence			
BG00864 (B)	42E5	*Tetraspanin 42Ef*	12 bp into gene (1st exon)	Unknown	Unknown
BG00890 (F)	Unknown	No sequence			
BG00907 (F)	Unknown	No sequence			
BG00915 (F)	50B1	*CG13334*/*CG13333*	350 bp upstream *CG13334*/850 bp downstream *CG13333*	L–lactate dehydrogenase activity/Unknown	oxidation reduction; cellular carbohydrate metabolic process; glycolysis/Unknown
BG01004 (F)	32E1	No gene in region			
BG01030 (A)	58D2	*Verprolin 1*/*mei–S332*	400 bp upstream *Verprolin 1*/850 bp into gene (2nd exon) *mei–S332*	actin filament binding/Unknown	regulation of cell shape; myoblast fusion; actin filament organization; positive regulation of actin filament polymerization/female meiosis; male meiosis; sister chromatid cohesion
BG01031 (A)	47A13	*pipsqueak*	7.3 kb into gene (3rd intron)	DNA binding	olfactory behavior; imaginal disc-derived wing morphogenesis
**BG01042 (F)**	35D2	*escargot*	500 bp downstream	specific RNA polymerase II transcription factor activity; RNA polymerase II transcription factor activity	central nervous system development; germ–line stem cell maintenance; regulation of compound eye pigmentation; olfactory behavior; asymmetric neuroblast division; maintenance of imaginal histoblast diploidy
BG01085 (F)	Unknown	No sequence			
BG01121 (F)	86C7	*CG14696*/*CG4674*	200 bp upstream *CG14696*/500 bp upstream *CG4674*	Unknown/Unknown	Unknown/Unknown
BG01283 (F)	9B11–12	*lethal (1) G0289*/*CG32679*	60 bp into gene (1st exon) *l(1)G0289*/250 bp downstream *CG32679*	Unknown/Unknown	Unknown/defense response
BG01345 (A)	75B4	*Ecdysone-induced protein 75B*	30 kb into gene (1st intron)	heme binding	molting cycle, chitin-based cuticle; antimicrobial humoral response; ecdysis, chitin-based cuticle; regulation of ecdysteroid metabolic process
BG01403 (A)	50B2	No gene in region			
BG01540 (B)	13F1	*scalloped*	350 bp upstream	specific RNA polymerase II transcription factor activity	sensory organ development; imaginal disc-derived wing morphogenesis; imaginal disc-derived leg morphogenesis; compound eye morphogenesis
BG01550 (F)	99B11	*kayak*	5.3 kb into gene (1st intron)	protein binding; sequence–specific DNA binding; RNA polymerase II transcription factor activity; DNA binding	anatomical structure development; organ development; cell motion; response to stress; ovarian follicle cell development; cell cycle; sensory organ development; response to external stimulus; organ morphogenesis; gamete generation
BG01551 (F)	30F5	*CG13130*/*big brain*	35 bp in 1st exon of *CG13130*/25 bp upstream *big brain*	Unknown/cation channel activity	Unknown/cell–cell adhesion; mesoderm development
BG01553 (F)	88A5	*forkhead box, sub–group O*	100 bp into gene (1st exon)	transcription factor activity	regulation of biological process; regulation of response to stimulus; response to stress; regulation of insulin receptor signaling pathway; response to DNA damage stimulus; response to bacterium; negative regulation of cell size; cellular macromolecule metabolic process; determination of adult life span; anatomical structure development; response to hormone stimulus
BG01615 (A)	39D2	*nervana 3*	110 bp into gene (1st exon)	sodium∶potassium-exchanging ATPase activity	potassium ion transport; ATP biosynthetic process; sodium ion transport
BG01677 (A)	9B12	*CG17841*	Adjacent	Unknown	Unknown
BG01700 (F)	49F10	*CG4630*/*CG4646*	200 bp upstream/40 bp downstream	carnitine transporter activity/Unknown	transmembrane transport/Unknown
BG01701 (F)	18C1	No gene in region			
BG01702 (A)	53D14	*Dek*	1 kb into gene (2nd intron)	nucleic acid binding	mRNA processing
BG01710 (A)	61E1	*Glucose transporter 1*	45 kb into gene (3rd intron)	glucose transmembrane transporter activity; GTP binding; protein binding	regulation of cell proliferation; regulation of cell cycle
BG01878 (B)	Unknown	No sequence			
BG01918 (A)	23A3	*Phosphoglycerate kinase*	1.8 kb upstream *P*	phosphoglycerate kinase activity	synaptic transmission
BG01950 (B)	Unknown	No sequence			
BG01976 (B)	Unknown	No sequence			
BG02019 (B)	25F5	*CG9171*/*CG14005*	15 bp in gene (1st exon) *CG9171*/300 bp downstream *CG14005*	N–acetyllactosaminide beta–1,6–N–acetylglucosaminyltransferase activity/Unknown	inter–male aggressive behavior/Unknown
BG02039 (A)	58E9	*Defense repressor 1*	22 kb into gene (1st intron)	protein binding; zinc ion binding	negative regulation of biosynthetic process of antibacterial peptides active against Gram–negative bacteria; immune response
BG02049 (B)	Unknown	No sequence			
BG02128 (B)	12E5	*lethal (1) G0007*	17.4 kb into gene (2nd intron)	RNA splicing factor activity, transesterification mechanism; ATP–dependent helicase activity; ATP–dependent RNA helicase activity	inter–male aggressive behavior; regulation of alternative nuclear mRNA splicing, via spliceosome
BG02257 (B)	5A12	*Trapped in endoderm 1*/*Gustatory receptor 5a*	Adjacent *Trapped in endoderm 1*/750 bp upstream *Gustatory receptor 5a*	See BG00218	See BG00218
BG02395 (B)	2B17	No gene in region			
BG02644 (B)	57E6	*Fkbp13*	400 bp into gene (1st intron)	FK506 binding; peptidyl–prolyl cis–trans isomerase activity	inter–male aggressive behavior

Candidate genes used for analysis of epistasis are shown in bold font. Letters in parenthesis after the Line name denote different co-isogenic *Canton S* host strains for *P{GT1}*–element insertion.

Only one of the *P*–element tagged candidate genes, *forkhead box*, *sub–group O* (*foxo*) has been previously implicated to affect adult life span [26], [34]. All others are novel candidate genes affecting longevity, and fall into a wide range of gene ontology categories, including early development, metabolism, chemosensation, immune response and transcription factors (Table 3).

Several of the *P*–elements inserted into identical or nearly identical positions: five inserts in the first intron of *mushroom–body expressed* (*mub*), two inserts 500 base pairs upstream of *polychaetoid* (*pyd*), two inserts adjacent to *CG9238*, two inserts in the *Tre1/Gr5a* intergenic region, and two inserts between *CG8418* and *Gef64C*. Since this screen is far from saturation, these sites likely represent hotspots for *P{GT1}* element insertion [65].

The effects of multiple inserts in the same genomic region are often, but not always, heterogeneous. Two of the inserts in *mub* affected both sexes, two were male–specific, and one was female–specific. One insert near *CG9238* was female–specific, while the other affected both sexes. One of the inserts in the *CG18418/Gef64C* intergenic region affected both sexes, while the other was strongly female-biased. On the other hand, both inserts in the *Tre1/Gr5a* intergenic region affected both sexes, and both inserts near *polychaetoid* were male–specific.

To add to the complexity, not all inserts in the same gene affect longevity in the same direction. The mutations in *esg*, *pyd* and *mub* associated with increased life span were all in the Canton S F genetic background. In addition, seven mutations in *esg*, two mutations in *pyd*, and two mutations in *mub* were associated with reduced life span in the initial screen. All of these mutations were in the Canton S A or B genetic backgrounds, and with one exception (*mub* ^BG02497^) were in different locations from the mutations in these genes associated with increased life span (Table S1).

### Analysis of revertant alleles

We re-mobilized the *P*–element insertions in three of the lines associated with increased life span (*mub* ^BG00043^, *crol* ^BG00346^ and *esg* ^BG01042^) to create revertant alleles in which the *P*–element was precisely (or nearly precisely) excised, while maintaining the co–isogenic background. We measured the life span of the revertant alleles, the parental strains, and the *P*–element insert line simultaneously. If the disruption of the adjacent gene by the *P*–element insertion causes the increase in life span, we expect that the life span of the revertant alleles will not be significantly different from the control. This expectation was realized for each of the revertant alleles.

**Figure 2:**
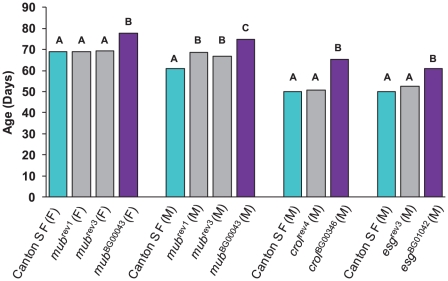
Analysis of revertant alleles. M denotes males and F denotes females. Canton S F control genotypes are depicted in cyan, and *mub* ^BG00043^, *crol* ^BG00346^ and *esg* ^BG01042^ genotypes in dark pink. Grey bars denote the revertant genotypes *mub* ^rev1^ (precise), *mub* ^rev3^ (imprecise), *crol* ^rev4^ (precise) and *esg* ^rev3^ (precise). The letters indicate the results of Tukey tests for significant differences between control, mutant and revertant lines. Genotypes with the same letter are not significantly different from each other.

The *mub* ^BG00043^ allele was associated with increased life span in both sexes. We obtained one precise revertant (*mub* ^rev1^) and one imprecise revertant (*mub* ^rev3^). Both revertant alleles had mean female life spans that fully reverted to the control, whereas the mean male life spans were intermediate between the control and mutant line (Figure 2). The *crol* ^BG00346^ and *esg* ^BG01042^ alleles were both associated with increased male life span, and the male life spans of the precise revertant alleles *crol* ^rev4^ and *esg* ^rev3^ were not significantly different from the control (Figure 2). These results show that the *P*–element mediated gene disruption is indeed responsible for the mutant phenotypes.

### Epistatic interactions among mutations that increase life span

Since all independently isolated long-lived *P*–element insertions result in increased life span, we asked whether these genes would be part of interacting genetic networks, and, if so, to what extent such networks would differ between the two sexes. We selected 10 *P*–element insertion lines in the Canton S F genetic background to assess epistatic interactions affecting life span, using a half–diallel crossing scheme in which all possible double heterozygotes were constructed (without reciprocals) [56]–[58]. The mean life spans of all double heterozygote genotypes are given in Table S2. We observed significant variation in life span among the double heterozygote genotypes (*P*<0.0001), between males and females (*P*<0.0001), and the genotype by sex interaction (*P*<0.0001) (Table S3). The effect of double heterozygous genotype was also highly significant in the individual analyses of males and females (Table S3); however, the cross–sex genetic correlation, *r_FM_* = −0.276±0.146, is not significantly different from zero (*t*_43_ = 1.88, *P*>0.05). Thus, the effects of the double heterozygous genotypes on life span are independent in the two sexes.

Variation among the double heterozygote genotypes can arise from two sources: variation in mean heterozygous effects of the different mutations, and variation from epistatic interactions.

Since all mutations are in the same co–isogenic background, all genetic variation among the genotypes must be attributable to one these sources. Diallel cross analysis enables us to partition the variation among the double heterozygous genotypes into their general (*GCA*) and specific (*SCA*) combining abilities. The *GCA* of each mutation estimates its average dominance in combination with all other mutations. The *SCA* of each double heterozygous genotype is the difference in the observed life span of the genotype from that expected given the *GCA*s of the two parental lines. Since alleles at all other loci are fixed and homozygous, any statistically significant *SCA* values must be due to dominance×dominance epistasis. We found significant variation in *GCA* and *SCA* values (*P*<0.0001) when pooled over both sexes, as well as significant *GCA*×Sex and *SCA*×Sex interaction terms (*P*<0.0001), indicating sex–specific *GCA* and *SCA* effects (Table S3).

**Figure 3:**
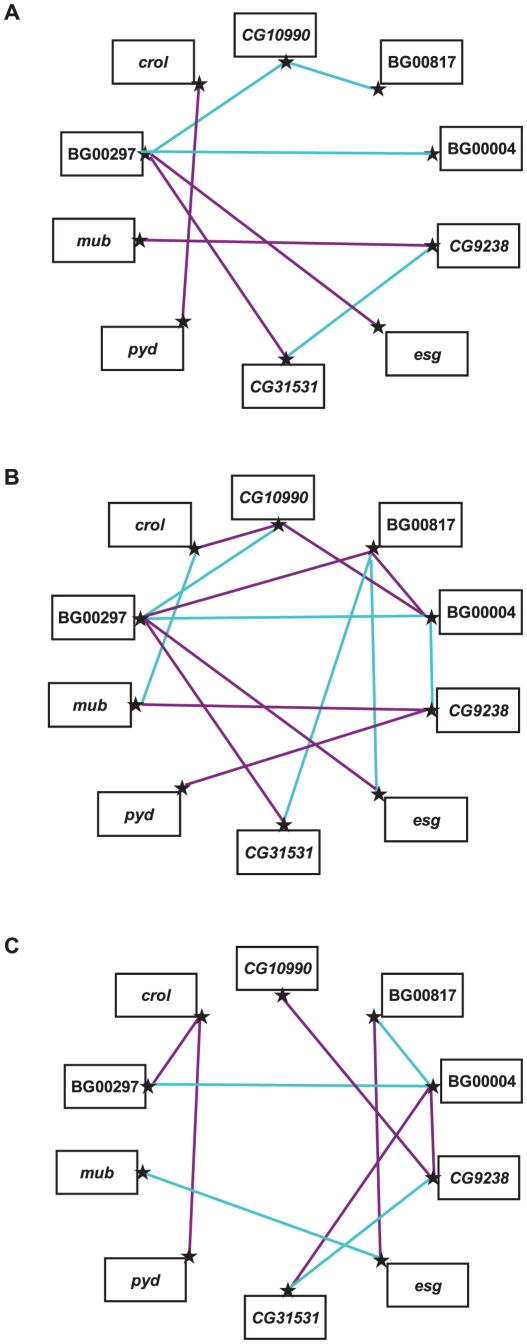
Epistatic interactions between *P*–element insert lines associated with increased life span. Significant *SCA* effects that enhance the mutant phenotype (*i.e.*, are longer-lived than expected) are indicated by dark pink lines, and significant *SCA* effects that suppress the mutant phenotype (*i.e.*, are shorter-lived than expected) are indicated by cyan lines. (A) Sexes pooled. (B) Males. (C) Females.

We estimated the *GCA* effects of each mutation and the *SCA* effects of all double heterozygous genotypes (Table S4). Epistatic effects can either suppress or enhance the mutant phenotype: the former occurs when the life span of a double heterozygote genotype is less than expected (closer to the wild–type, with a negative *SCA*), and the latter when the life span of the double heterozygote genotype is greater than expected (longer-lived, with a positive *SCA*). We identified eight statistically significant epistatic interactions in the analysis pooled across sexes; 10 significant interactions for females and 14 significant interactions for males (Figure 3, Table S4). The cross–sex correlation of *SCA* values was *r_FM_* = −0.137±0.151, which is not significantly different from zero (*t*_43_ = 0.907, *P*>0.05). Thus, when examined separately the two sexes displayed vastly different epistatic interactions (Figure 3, Table S4). Of the 21 significant epistatic interactions in males and/or females, only one was common to both sexes, 16 were unique to each sex, and three epistatic interactions were sexually antagonistic, with enhancing effects in one sex and suppressing effects in the other (BG00817–BG00004, BG00817–*esg*, BG00004–*CG9238*).

### Pleiotropic effects of mutations increasing life span

**Figure 4:**
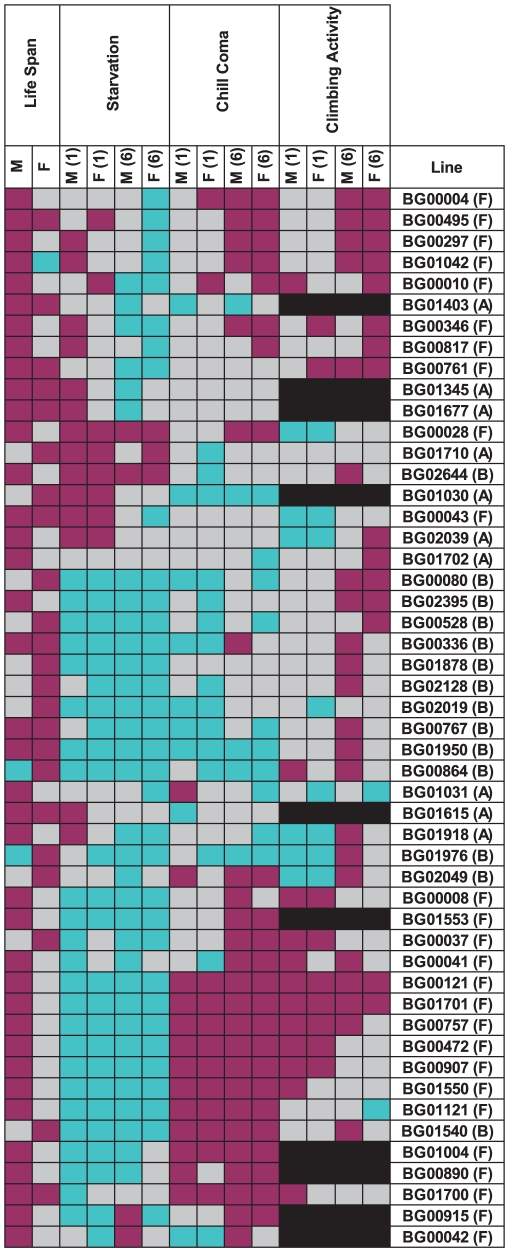
Pleiotropic effects of *P*–element insert lines associated with increased life span on starvation resistance, chill coma recovery, and climbing activity in males (M) and females (F) at one (1) and six (6) weeks of age. Dark pink indicates increased fitness (greater resistance to starvation stress and climbing ability, reduced time to recover from chill coma), and cyan indicates decreased fitness (less resistance to starvation stress and climbing ability, increased time to recover from chill coma), relative to the co–isogenic control; grey indicates no significant difference from the control; and black indicates that the measurement was not done.

We assessed whether mutations with significantly increased life span had pleiotropic effects on stress resistance (chill coma recovery and starvation resistance) as well as a general measure of health (climbing activity) at one week and six weeks of age. We observed substantial pleiotropy. Of the 50 lines tested for starvation resistance, 44 were significantly different from the control at one week (16 with increased starvation resistance and 28 with decreased starvation resistance in one or both sexes), and 46 were significantly different from the control at six weeks (five with increased starvation resistance and 42 with decreased starvation resistance – one line had sexually antagonistic effects) (Figure 4, Table S5). Of the 50 lines tested for chill coma recovery, 32 were significantly different from the control at one week (15 with decreased chill coma recovery times and 17 with increased chill coma recovery times), and 42 were significantly different from the control at six weeks (29 with decreased chill coma recovery times and 13 with increased chill coma recovery times) (Figure 4, Table S5). We only assessed 40 of the lines for climbing ability. Of these, 23 were significantly different from the control at one week (14 with increased climbing ability and nine with decreased climbing ability), and 30 were significantly different from the control at six weeks (28 with increased climbing ability and two with decreased climbing ability) (Figure 4, Table S5). Thus, on average, by six weeks of age the lines with increased longevity have overall decreased resistance to starvation stress, but increased resistance to chill coma stress and increased general activity relative to the controls.

There was significant variation among the lines and significant sex by line interactions for all three traits (Table S6), indicating that the mutations do indeed have heterogeneous pleiotropic effects, and that the effects are sex–specific. Broad sense mutational heritabilities ranged from *H* ^2^ = 0.43–0.60 for starvation resistance and chill coma recovery, but were lower for climbing ability (*H* ^2^ = 0.21 averaged over week 1 and week 6 measurements) (Table 4). Although all cross–sex genetic correlations were significantly different from unity, the estimates of *r_MF_* were high for all traits except for climbing ability at six weeks (Table 4).

**Table 4:** Effects of mutations increasing life span on starvation resistance, chill coma recovery, and climbing ability.

Trait	Week	Mean	*H* ^2^ a	*r_MF_* b
Starvation resistance	1	39.24	0.608	0.698
	6	28.70	0.434	0.686
Chill coma recovery	1	10.95	0.464	0.826
	6	19.30	0.545	0.898
Climbing Activity	1	14.15	0.259	0.871
	6	6.23	0.163	0.398

**^a^**Broad sense heritability, *H* ^2^ = (*σ_L_* ^2^+*σ_SL_* ^2^)/(*σ_L_* ^2^+*σ_SL_* ^2^+*σ_E_* ^2^).

**^b^**Cross-sex genetic correlation, *r_MF_* = *σ_L_* ^2^/(*σ_LM_ σ_LF_*).

If the mutations affecting increased life span are generally more robust, we would expect positive correlations between life span and stress resistance and general health, expressed as deviations from the control. Similarly, if the mutations affecting increased life span have delayed senescence, the correlations between longevity and the other traits should be positive at six weeks of age, when the control line flies are beginning to die, but the long-lived mutant individuals are still alive. However, this was not the pattern observed. We consider the overall pleiotropic effects of the mutations separately for males and females, since the effects of the mutations on life span were not correlated between the sexes. In females, the correlation (±SE) between longevity and chill coma recovery time was positive and significant at both one week (*r* = 0.328±0.136, *t*_48_ = 2.41, *P* = 0.020) and six weeks (*r* = 0.418±0.131, *t*_48_ = 3.19, *P* = 0.0025) (Table 5). Thus, there is a tendency for mutations affecting female life span to be inversely associated with resistance to chill coma stress, at either age. The correlation between starvation resistance and climbing ability was significant and negative at one week (*r* = −0.329±0.153, *t*_38_ = 2.15, *P* = 0.038). None of the other correlations were significantly different from zero (Table 5). In males, however, the correlation (±SE) between longevity and starvation resistance was positive and significant at both one week (*r* = 0.303±0.138, *t*_48_ = 2.20, *P* = 0.038) and six weeks (*r* = 0.554±0.120, *t*_48_ = 4.61, *P* = <0.0001) (Table 5). Further, the correlation between male life span and chill coma recovery time was negative and significant at six weeks (*r* = −0.577±0.118, *t*_48_ = 4.90, *P* = <0.0001) (Table 5). Thus, mutations affecting male life span do show the expected positive associations with stress resistance and delayed senescence for stress resistance. However, the correlation between male starvation stress resistance and climbing activity was significant and negative at one week (*r* = −0.619±0.127, *t*_38_ = 4.86, *P* = <0.0001); *i.e.*, mutations associated with increased stress resistance were less active (Table 5).

**Table 5:** Mutational correlations among life span, starvation resistance, chill coma recovery, and climbing ability.

		Week 1				Week 6			
		Males				Males			
		LS	SR	CC	CA	LS	SR	CC	CA
	**LS**		**0.303**	−0.153	0.159		**0.554**	**−0.577**	−0.149
	**SR**	−0.080		0.236	**−0.619**	−0.093		−0.162	−0.145
	**CC**	**0.328**	−0.266		−0.282	**0.418**	0.096		−0.024
	**CA**	−0.083	**−0.329**	−0.238		0.151	0.048	−0.060	

Correlations in bold font are significantly different from zero. LS = life span, SR = starvation resistance, CC = chill coma recovery, CA = climbing activity.

The combination of significant pleiotropy but little directional correlation between longevity and other traits indicates that the pleiotropic effects are highly variable, as illustrated in Figures S1, S2, S3, S4. The complex pattern of variation in pleiotropic effects among the *P*–insert lines associated with increased life span in at least one sex is depicted in Figure 4. Notably, all of the mutations are associated with at least one deleterious pleiotropic effect on stress resistance or general health, indicating the complicated mutational basis of trade–offs between putative fitness components.

### Effects of mutations increasing life span on whole-genome transcript abundance

**Figure 5:**
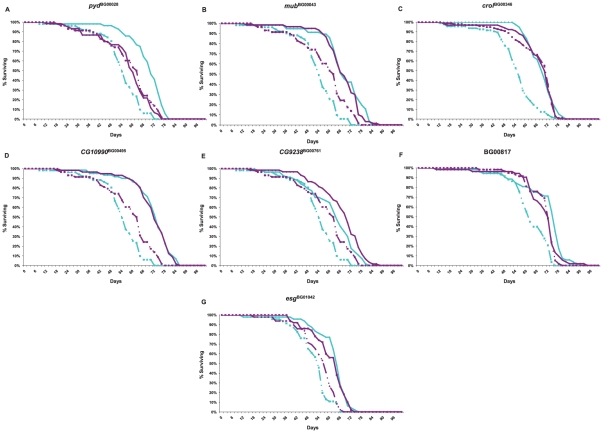
Survival curves of *P*–element insertion lines associated with increased life span (diamonds and solid lines) and the co–isogenic control line (Canton S F, squares and dashed lines) used for whole genome microarray profiling. Cyan lines denote males; dark pink lines denote females. (A) *pyd* ^BG00028^; (B) *mub* ^BG00043^; (C) *crol* ^BG00346^; (D) *CG10990* ^BG00495^; (E) *CG9238* ^BG00761^; (F) BG00817; (G) *esg* ^BG01042^.

Genetic networks of mutations that affect a common phenotype can serve as focal points for the identification of additional candidate genes affecting that phenotype by transcript profiling [59]. Transcripts that are co-regulated in the genetic background of the mutant lines are themselves candidate genes affecting longevity, and the clustering of co-regulated transcripts can yield insights about the function of predicted genes tagged by the mutations. We assessed the extent to which seven of the mutations associated with increased life span (*pyd* ^BG00028^, *mub* ^BG00043^, *crol* ^BG00346^, *CG10990* ^BG00495^, *CG9238* ^BG00761^, BG00817 and *esg* ^BG01042^) affected whole genome transcriptional regulation. We performed these analyses at six weeks of age for all mutant lines and the Canton S F co–isogenic control – the age at which the control lines are beginning to die, but at which most of the *P*–element insert lines remain alive, and at which we assessed differences among these lines in senescence. The survival curves for this experiment are given in Figure 5. We independently confirmed the effects of all mutations on life span, with one exception. In our initial and secondary screens, *mub* ^BG01042^ females had reduced longevity, but in this assay, both males and females were long-lived.

Not all transcripts on the array are expressed in six week old adults. We eliminated all transcripts that were not considered present in both replicates of at least one line and sex, leaving 12,636 transcripts for analysis. We performed several analyses of variation of gene expression (Table S7). First, we assessed the extent to which there was variation in the main effects of sex, genotype, and the genotype by sex interaction among all lines for each transcript, using a false discovery rate criterion to account for multiple tests [66]. At a *q*–value of 0.001 (0.0001), we found 11,111 (10,603) sexually dimorphic transcripts. Remarkably, genotype was significant for 4,488 transcripts (35.5%) at *q*≤0.001, and 1,996 transcripts (15.8%) at *q*≤0.0001. The genotype by sex interaction was significant for 1,621 transcripts (12.8%) at *q*≤0.001, and 434 transcripts (3.4%) at *q*≤0.0001. We also ran reduced ANOVAs separately for each sex, and for each of the mutant lines compared to the control. A total of 619 and 561 transcripts were significant at *q*≤0.001 for females and males, respectively. The magnitude of transcriptional co–regulation varied among the mutant lines. At a significance level of *q*≤0.05, we observed 276 significant transcripts for *CG10990* ^BG00495^; 313 for *pyd* ^BG00028^; 777 for *CG9238* ^BG00761^; 1,815 for BG00814; 2,141 for *crol* ^BG00346^; 2,193 for *esg* ^BG01042^; and 3,969 for *mub* ^BG00043^.

We analyzed the Biological Process Gene Ontology (GO) categories represented by the significant transcripts to determine if particular categories are over-represented. In the separate sex analyses of all genotypes, there was over–representation of significant transcripts in the DNA integration, metabolism (particularly carbohydrate metabolism) and proteolysis categories in both sexes (Table S8). Genes affecting detection of external stimuli, particularly light and abiotic stimuli, were enriched in females, while genes affecting mating and reproductive behavior and muscle development were enriched in males (Table S8).

**Figure 6:**
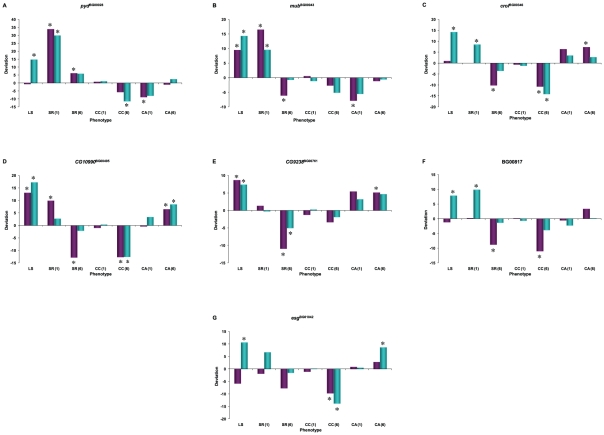
Pleiotropic effects on of *P*–element insertion lines associated with increased life span used for whole-genome microarray profiling, expressed as deviations from the co-isogenic Canton S F control line. SR: Starvation resistance; CC: chill coma recovery; CA: climbing activity. Numbers in parentheses refer to assays at week 1 or week 6. Cyan denotes males; dark pink denotes females. (A) *pyd* ^BG00028^; (B) *mub* ^BG00043^; (C) *crol* ^BG00346^; (D) *CG10990* ^BG00495^; (E) *CG9238* ^BG00761^; (F) BG00817; (G) *esg* ^BG01042^. Asterisks denote significant deviation from the control.

Although all of the mutations assessed are long-lived, they have variable and sex–specific pleiotropic effects on longevity, resistance to starvation and chill coma stress, and climbing activity (Figure 4 and Figure 6). Thus, we expected to find both common and variable patterns of transcriptional co–regulation among the mutations. This is indeed what we observed.

*pyd* affects the biological processes of the cell–cell adhesion, fusion cell fate specification and branch fusion in the open tracheal system (Table 3). Over-represented co-regulated transcripts in the *pyd* ^BG00028^ mutant background fell into the categories of DNA integration; prosthetic group, pyruvate, nucleoside, lipid, chitin and glucosamine metabolism; proteolysis; and mating and reproductive behavior (Table S8).

*mub* is a regulator of alternative nuclear mRNA splicing via the spliceosome, and is hence likely to have far-reaching pleiotropic effects (Table 3). Categories that are over-represented among co-regulated *mub* ^BG00043^ probe sets are consistent with this annotation, and include DNA replication and repair, RNA processing, the cell cycle, and chromatin modification and silencing. However, the largest over-represented categories in this mutation were in DNA, RNA, cellular and macromolecular metabolism (Table S8).

*crol* is an RNA polymerase II transcription factor that has pleiotropic effects on cell adhesion and proliferation, regulation of transcription, wing morphogenesis and regulation of the mitotic cell cycle and the Wnt receptor signaling pathway. Over-represented transcripts in the *crol* ^BG00346^ mutation primarily affect ribosome biogenesis, histone mRNA 3′ end processing and metabolism, transcription, protein metabolism and proteolysis, sleep, and reproductive and mating behavior (Table S8).

*CG10990* is a predicted gene of unknown function (Table 3). The top over-represented GO categories in *CG10990* ^BG00495^ mutants are DNA integration, peptidyl–proline modification and amino acid derivative metabolism; but insulin signaling, proteolysis, and mating, reproductive and locomotor behavior are also over-represented (Table S8).

*CG9238* is a predicted gene that is annotated to regulate protein phosphatase type 1 activity. Type 1 protein phosphatase is involved in the regulation of many processes so it is not that surprising that the *CG9238* ^BG00761^ mutant background is over-represented in several categories, including metabolism, embryonic and larval development, as well as visual, locomotor, mating, reproductive and rhythmic (circadian) behaviors (Table S8).

We do not know the exact insertion site of the *P*–element in line BG00817. However, many GO categories associated with muscle development are highly over-represented among significant co-regulated transcripts in this line. Lipid catabolism, proteolysis, and lipid, carbohydrate and protein metabolism are also over-represented (Table S8).

*esg* is an RNA polymerase II transcription factor with pleiotropic effects on multiple biological processes: central nervous system development, germ–line stem cell maintenance, regulation of compound eye pigmentation, olfactory behavior, asymmetric neuroblast division and maintenance of imaginal histoblast diploidy. The large number of over-represented GO categories among the co-regulated transcripts in the *esg* ^BG01042^ mutation is consistent with highly pleiotropic functions of *esg*. Genes involved in RNA processing and localization, ribosome biogenesis, RNA and DNA metabolism, primary metabolism and fertilization are over-represented. However, the most significant over-representation of co-regulated transcripts in *esg* ^BG01042^ is related to vision (response to light, visual perception, phototransduction) (Table S8).

Since all seven mutant lines have increased life span relative to the control, we sought to define the transcriptional signature of increased life span from the probe sets with common patterns of co–regulation across multiple lines. A total of 553 transcripts were common to four or more of the mutant lines; of these, 187 probe sets were up-regulated relative to the control and 270 were down-regulated relative to the control (Table S9). The up-regulated probe sets are enriched for genes affecting proteolysis, whereas the down-regulated transcripts are enriched for genes affecting gene expression and RNA metabolism. However, the transcriptional signature of increased life span is most notable for the large number of computationally predicted transcripts of unknown function as well as the diversity of biological functions represented. The transcripts in common to four or more of the mutant lines are encoded by genes affecting reproduction, chemosensation, metabolism, immunity/defense response, function of the nervous system and development.

Genes that are co-regulated in the mutant backgrounds are themselves candidate genes affecting life span. Therefore, we tabulated variation in gene expression of known genes affecting life span in the mutant lines associated with increased life span (Table S10). First, five of the six focal genes for which we know the genes tagged by the *P*–element (*pyd*, *mub*, *CG10990*, *CG9238* and *esg*) are themselves significantly differentially expressed in the analysis considering all genotypes. Three of these genes (*mub*, *CG10990* and *CG9238*) are also differentially expressed relative to the control in their own mutant backgrounds. Further, *mub* is differentially expressed in the *pyd* ^BG00028^ and *esg* ^BG01042^ mutant lines, and *esg* is differentially expressed in the *pyd* ^BG00028^, *crol* ^BG00346^ and *CG9238* ^BG00761^ mutant lines. Six additional genes in which *P*–element mutations were associated with increased life span in our screen were differentially regulated among the seven mutations profiled in the array analysis (*CG31531*, *Trapped in endoderm–1*, *CG18418*, *meiotic from via Salaria 332*, *kayak* and *Dek*). A further 13 genes in which *P*–element insertions were associated with decreased life span had differentially regulated transcripts in the mutant backgrounds (*CG14478*, *CG31176*, *CG4004*, *CG6854*, *couch potato*, *inaF*, *ken and barbie*, *Laminin A*, *Lipid storage droplet–2*, *Malic enzyme*, *Protein kinase 61C*, *Rab23* and *singed*). Finally, eight genes in which mutations have been described to negatively regulate life span were also differentially co-regulated in the mutant backgrounds (*I'm not dead yet*, *chico*, *Insulin–like receptor*, *Superoxide dismutase*, *Alcohol dehydrogenase*, *Sirt2*, *Vacuolar H^+^–ATPase SFD subunit* and *CTP:phosphocholine cytidylyltransferase 1*).

## Discussion

### The mutational landscape of longevity

We performed an unbiased, forward genetic screen of 1,332 *P{GT1}* insertional mutations that were generated in one of six Canton S co–isogenic backgrounds for mutations affecting *Drosophila* longevity. In the initial screen, we identified 436 (32.7%) mutations with mean life spans that were significantly different from their co–isogenic control. Of these, 296 (67.89%) were associated with reduced life span, 135 (30.96%) were associated with increased life span, and 5 (1.15%) had sexually antagonistic effects on life span. The sample size per mutation in the initial screen was not large; therefore, many of the significant effects could be false positives. Nevertheless, if nearly one–third of mutations affect life span, the mutational target size for longevity must be large, consistent with the many mechanisms that are known to affect life span. We know the locations of the *P*–element inserts for 290 of the mutations associated with significant effects on longevity. Of these, 56 map to gene deserts (regions of the genome with no computationally predicted genes) and likely define novel un-annotated genes. With the exception of *foxo* [26], [34], none of the mutations tagged genes that have been previously associated with life span. Thus, forward genetic screens for mutations with subtle, quantitative effects on life span in a co–isogenic background is an efficient method for identifying novel genes affecting longevity and other complex traits [57], [58], [62], [63], [67].

Substantially more mutations were associated with decreased than increased life span. It is generally assumed that mutations decreasing life span are less interesting than mutations increasing life span, since the former category of mutations could be generally deleterious and affect all aspects of fitness, while the latter are more likely to have specific effects on life span. Thus, we concentrated on confirming the effects of mutations associated with increased life span with larger sample sizes in a secondary screen, and identified 58 mutations associated with increased life span. The mutations associated with significant increases in life span represent pathways known to affect life span (e.g., the insulin and metabolic pathways), as well as novel pathways involving taste, the nervous system and embryonic development.

Mutations reducing life span are typically inferred to be in genes required for normal life span; over–expression of such genes may extend longevity, as has been observed for dFOXO [26]. Conversely, mutations increasing life span are thought to be in genes that normally function to limit life span; reducing expression of these genes thus extends longevity [27], [68]. This logic presumes that all mutations in genes affecting life span have effects in the same direction. The proclivity of *P*–elements to insert in genomic hot–spots generated many insertions in the same genes enabled us to observe directly the distribution of mutational effects in the same genes. While many mutations in the same genes did indeed have similar effects on life span, this was not always true. Mutations in the same gene can be associated with both increased and decreased life span, often in a sex–specific manner, depending on the location and orientation of the *P*–element insertion, and genetic background. Examples include insertions in the *Tre1/Gr5a* intergenic region [63], *mub*, *pyd* and *Defense repressor 1* (*Dnr1*) (Table 2, Table S1). These observations highlight the inaccuracy of referring to genes that are required for normal life span or that normally limit life span. Mutational analysis identifies genes that are relevant to the modulation of life span, but variable allelic effects preclude inferring directionality of wild type function.

### Epistatic interactions among mutations increasing life span

Mutations in different locations in the same gene could have variable effects on longevity if they interfere with different aspects of gene regulation, or if some are in regulatory regions and others directly affect the protein. Different mutational effects could also arise due to variation in the amount of the vector inserted into the genome or by partial genomic excision during the insertion process. Variable effects of mutations in the same location and orientation but different genetic backgrounds may also be attributable to epistatic interactions with different alleles. Indeed, diallel crosses among just 10 of the mutations associated with increased life span revealed a surprisingly complex network of epistatic interactions involving all 10 mutations, suggesting pervasive epistasis between alleles affecting life span.

Mutations in the *Tre1/Gr5a* intergenic regions interact epistatically with mutations in genes affecting insulin signaling [63]. It will be interesting to determine to what extent the other mutations interact with components of this well-established pathway, and to what extent the effects on life span are independent of insulin signaling. Epistasis has repeatedly been observed between QTL alleles affecting variation in life span [69], [70] as well as between QTL alleles without main effects on life span [71], although the identities of the genetic loci underlying the QTLs are not known.

Further evidence for the importance of epistasis in the genetic architecture of *Drosophila* life span comes from observations that the effects of transgene over–expression and single mutations on longevity vary according to genetic background. The effect on increased life span of over-expressing *Drosophila Superoxide dismutase* was greater in the background of a relatively short-lived strain than in a long-lived strain background [72]. Similarly, the *Indy* mutation increased life span by 40–80% in the short-lived *Shaker*, *Hyperkinetic* and *drop dead* strains, but only by 15% in a strain selected for increased life span [52]. Over–expression of human *SOD* in *Drosophila* motor neurons increases life span [7], but the magnitude of the increase varies in different wild type genetic backgrounds in a sex–dependent manner [73]. Likewise, introgression of each of three morphological mutations into seven wild-derived backgrounds showed considerable background–dependent effects on life span [74]. These observations highlight the importance to assess the effects of the mutations increasing life span in a range of naturally derived genetic backgrounds, and to identify the genes with which the mutations interact.

### Sex-specific effects of mutations on life span

A striking feature of our screen is that the effects of mutations increasing life span are highly sex–specific, with a low, but significant, negative cross sex–genetic correlation of *r_MF_*∼−0.3. Epistatic effects were similarly sex–specific, and in three cases the direction of the epistasis was opposite in males and females. This observation is consistent with previous studies documenting sex–specific effects on life span, beginning with Maynard Smith's [75] analysis showing that the genetic control of longevity was independent in *D. subobscura* males and females. More recently, QTLs affecting variation in life span between two laboratory strains, Oregon and 2b, have sex–specific effects [69], . Most studies of aging examine only one sex [80], but when both sexes are included, sex–specific mutational effects are surprisingly common. For example, the effects of mutations in the *Drosophila insulin–like receptor* (*InR*) [16], the insulin receptor substrate *chico* [15] and *DTS–3*, a gene involved in ecdysone biosynthesis [54] had female-biased or female–specific effects on life span. As noted above, over–expression of human *SOD* in *Drosophila* motor neurons in different genetic backgrounds has sex–specific effects on life span [73]. Further, the benefits of dietary restriction on increased life span of *D. melanogaster* are greater in females than males [81]. Conditional over–expression of both wild type and mutant *p53* transgenes has sexually antagonistic effects on male and female life span that are in opposite directions depending on the developmental stage of over–expression [82].The causes of the sex–specific effects remain mysterious [80]. However, it should be noted that sex–specific effects of mutations and QTLs are a common feature of the genetic architecture of complex traits in *Drosophila* and other organisms [83], although such effects on life span are particularly extreme. It remains to be seen whether a common mechanism underlies sex–specificity for all traits.

### Pleiotropic effects of mutations increasing life span on organismal phenotypes

The concept of trade–offs (antagonistic pleiotropy) is central to many evolutionary hypotheses for limited life span and senescence. Such trade–offs were historically envisioned to be governed by alleles with beneficial fitness effects early in life, when the force of natural selection is strong, but detrimental effects later in life, when natural selection is weak [2], [28]. Kirkwood [84] phrased this concept in terms of a physiological trade–off caused by the need to optimally allocate resources to reproduction and somatic maintenance. Support for antagonistic pleiotropy comes from quantitative genetic studies documenting negative genetic correlations between early and late fitness components [28]–[31], [85], [86]; but these negative genetic correlations are not always found [87]–[90]. *Drosophila* mutations affecting increased life span often exhibit antagonistic pleiotropy: mutations in *chico* and *InR* show a dwarf phenotype and have reduced fecundity [15], [16], and mutations of *Indy* have decreased fecundity under adult caloric restriction [91].

We have shown here that antagonistic pleiotropy is pervasive, in that all *P*–element insert lines associated with increased longevity were also associated with at least one deleterious pleiotropic effect on resistance to starvation stress, recovery after chill coma, and/or a general measure of health (climbing activity) at one week and/or six weeks of age (Figure 4). On average, the lines with increased longevity have overall decreased resistance to starvation stress and increased resistance to chill coma stress and increased general activity relative to the controls at six weeks of age. Mutations in genes in the insulin signaling pathway tend to have increased resistance to starvation and oxidative stress, accompanied by a trade–off in growth and fecundity [23], [25], [26], [32], [33], [92]–[94]. Thus, our observation that resistance to starvation stress actually decreases in older flies from the long-lived strains runs counter to this theme. It will be interesting in the future to assess early and late age fecundity on these mutations. However, it should be noted that the negative genetic correlation between the sexes for longevity is itself a trade–off, and that patterns of pleiotropy are different for males and females. Mutations affecting female life span have antagonistic pleiotropic effects on resistance to chill coma stress. Mutations affecting male life span have positive pleiotropic effects on resistance to starvation and chill coma stress, but there is antagonistic pleiotropy between male starvation stress resistance and climbing activity.

### Pleiotropic effects of mutations increasing life span on gene expression

Whole genome expression profiling of mutations that have been derived in the same co–isogenic background is a powerful tool for identifying networks of co-regulated genes that potentially affect the trait(s) affected by the mutations. Taken at face value, our analysis of gene expression of six week old adults in seven mutant lines associated with increased life span and the control strain indicate that many genes affect life span. We identified 4,488 transcripts that were differentially expressed among all eight genotypes using a false discovery rate criterion of *q*≤0.001 [66]. Transcripts from many of the candidate genes identified in the *P*–element screen and from genes that have been previously shown to affect life span were also differentially expressed in the background of the seven focal lines. This suggests that the co-regulated genes are indeed excellent candidate genes affecting life span. The fact that transcripts of three of the focal mutations were differentially expressed in the appropriate mutant background provides independent evidence that the *P*–element does affect the gene in which it has inserted. Further, mutations in co-regulated genes may interact epistatically with mutations in the focal genes [59], defining genetic networks affecting longevity. The large number of co-regulated genes in each mutant background is consistent with the large number of epistatic interactions we observed among just 10 mutations associated with increased life span. The mutations in *pyd*, *mub*, *crol*, *CG10990* and *esg* affected a diverse array of biological processes that were somewhat unexpected, given their functional annotations. For example, these genes were not expected *a priori* to affect metabolism and reproduction; yet these categories were over-represented overall. These observations suggest that these loci may interact with insulin signaling and other well-described pathways affecting life span.

Several other studies have reported whole genome changes in gene expression in aging *Drosophila* and *C. elegans*. Pletcher *et al.* [40] examined both aging and caloric restriction, and found considerable over–representation for biological processes involving the cell cycle, metabolism, DNA repair and replication, transcription, RNA processing, gametogenesis and perception of light. Similarly, we observed over–representation of gene ontologies for metabolism, cell cycle, mating behavior and response to light. Unfortunately, the expression data of Pletcher *et al.* [40] are not publicly available, precluding a direct comparison of the lists of genes that were co-regulated by mutation associated with increased life span and those implicated in the analysis of normal aging and prolonged life span through caloric restriction.

However, we were able to compare the genes that were co-regulated in the seven *P*–element lines associated with increased life span with the analysis of normal aging in two *Drosophila* strains [44]. We observe extensive overlap with the 48 candidate genes postulated by Lai *et al.* [44], on the basis that they exhibited significant changes in transcript abundance with age and between the two strains, and that were located in known life span QTL [74], [75], [80], [87]. Almost 23% (11) of these genes were significantly different between our genotypes at *q*<0.0001, 50% (24) were significant between our genotypes at *q*<0.001.

There was also significant overlap of the genes that were co-expressed in *Drosophila* mutations associated with increased life span with many of the *C. elegans* orthologs that were co-regulated in the long-lived *daf–2* mutant background [59]. 30 of the 39 up-regulated genes and 11 of the 20 down-regulated genes identified by Murphy *et al.* [60] had *Drosophila* homologs. 30% (9) of the up-regulated genes were significant in our study at *q*<0.0001, and 63% (19) were significant at *q*<0.001. For the down-regulated genes, only 27% (3) were significant at *q*<0.001 (none were significant at *q*<0.0001). These numbers are slightly inflated as several heat shock genes in *C. elegans* are homologous to a single *Drosophila* gene, *lethal (2) essential for life*.

Many genes that have been previously shown to affect life span showed differential expression in the mutant lines associated with increased life span. For example, *InR* was down-regulated in both the *CG9238* and *CG10990* backgrounds, consistent with the previously observed decrease of *InR* expression associated with increased life span [16]. *Alcohol dehydrogenase* (*Adh*) was up-regulated in the mutant *pyd*, *mub* and *esg* backgrounds. *Adh* expression has been shown to decrease with age [37] so an increase in expression could conceivably be associated with an increase in life span. The expression of *Sirt2*, a member of the *Drosophila* Sirtuin family [95], was strongly decreased in the *mub*, BG00817 and *esg* mutant backgrounds. The *mub* mutant displayed an increase in *Sod* expression and a decrease in *chico* expression which mirrors previous reports of changes of the expression of these genes in association with increased life span [7], [15].

### Conclusions and future prospects

We performed an unbiased, forward genetic screen for mutations affecting *Drosophila* longevity, and identified 58 mutations associated with increased life span. These mutations represent pathways known to affect life span (e.g., the insulin and metabolic pathways, gene silencing and immune response), as well as novel pathways involving taste and nervous system and embryonic development. Mutations in the same gene can be associated with both increased and decreased life span, which could be caused by different insertion sites or epistatic interactions with different genetic backgrounds. Pervasive epistasis for mutations affecting life span is indicated by a diallel cross analysis of ten of the mutations associated with increased life span. A striking feature of our screen is that the main and epistatic effects of mutations increasing life span are highly sex–specific. Further, antagonistic pleiotropy of mutational effects is pervasive, in that all *P*–element insert lines associated with increased longevity were also associated with at least one deleterious pleiotropic effect on a component of fitness. However, the patterns of pleiotropy are also sex–specific and different for males and females. The 4,488 transcripts that are differentially expressed among all eight genotypes provide a glimpse into complex genetic networks affecting longevity, which include many genes previously shown to affect life span. Further studies are required to establish that *P*–element disruptions of all candidate genes cause the changes in longevity and to determine interactions of these novel mutations with mutations in genes of the insulin signaling pathway and other pathways known to affect life span. The causes of the sex–specific and background–dependent epistatic effects remain to be elucidated, as do any effects on early and late reproduction, and the contribution of the novel loci to naturally occurring variation in life span – in *Drosophila*, and other organisms.

## Materials and Methods

### *Drosophila* stocks

The *P{GT1}* insertion lines [55] used in this study were constructed in six co–isogenic *w^1118^* Canton–S backgrounds (A, B, C, D, E and F) as part of the Berkeley *Drosophila* Gene Disruption Project [55], and were obtained from Hugo Bellen (Baylor College of Medicine, Houston, TX). All lines were maintained at 60–80% humidity and 25°C under a 12∶12 hour light∶dark cycle.

### Screen for mutations affecting life span

We screened 1,332 homozygous viable *P{GT1}* insertion lines for changes in life span relative to their control line. The initial screen was conducted in blocks of 50–100 insert lines and the appropriate Canton S control line. Each block was initiated with virgin males and females that had eclosed within 48 hours of each other, with two replicate vials per sex per insert line, and ten replicate vials per sex of the control line. Each replicate vial contained five flies of the same sex and 5 ml cornmeal/molasses medium. We recorded deaths every two days until all flies were dead, and transferred the flies to fresh culture medium every 1–2 days.

We evaluated mutational variation for life span using analyses of variance (ANOVAs) of the mean life span of replicate vials, expressed as deviations from the appropriate contemporaneous control means for each sex. The full two–factor mixed effects model for pooled sexes was *Y* = *μ*+*L*+*S*+*L*×*S*+*ε*, and the reduced model for the analysis of sexes separately was *Y* = *μ*+*L*+*ε*, where *μ* is the overall mean, *L* is the line effect (random), *S* is the sex effect (fixed) and *ε* is the environmental variance between replicate vials. We computed the mutational broad sense heritability (*H_M_* ^2^) from the full model as *H_M_* ^2^ = (*σ_L_* ^2^+*σ_SL_* ^2^)/(*σ_L_* ^2^+*σ_SL_* ^2^+*σ_ε_* ^2^), where *σ_L_* ^2^, *σ_SL_* ^2^ and *σ_ε_* ^2^ are, respectively, line, sex by line, and environmental variance components; and the cross–sex genetic correlation (*r_MF_*) as *r_MF_* = *cov_FM_*/*σ_LF_σ_LM_*, where *cov_FM_* is the covariance between the mean life span of males and females, and *σ_LF_* and *σ_LM_* are the square roots of the variance components from the separate sex analyses of females and males, respectively.

We used two methods to identify insert lines with mean life spans that were significantly different from the control. We computed the 95%, 99% and 99.9% confidence intervals of the deviations from the control mean, assuming normality, as ±*z_α_ σ*/(*n*)^½^. *z_α_* is the critical value for the normal distribution (1.96, 2.575 and 3.3 respectively for the 95%, 99% and 99.9% confidence intervals). *σ* is the phenotypic standard deviation, estimated as (*σ_L_* ^2^+*σ_SL_* ^2^+*σ_ε_* ^2^)^½^ for the full model and (*σ_L_* ^2^+*σ_ε_* ^2^)^½^ for the reduced models. *n* is the number of replicate vials for each insert line (*n* = 4 in the full model and *n* = 2 in the reduced models). We also used the Dunnett's *t*–test, which corrects for multiple tests of different insert lines relative to a common control, to identify insert lines that were significantly different from the control within each block.

We re-assessed the life span of 83 lines with increased life span under the same conditions as the previous assay, but with larger sample sizes of at least 12 replicate vials per sex per line. We evaluated the significance of the difference in life span between each insert line and the control by ANOVAs pooled across sexes and for each sex separately, using the models *Y* = *μ*+*L*+*S*+*L*×*S*+*R*(*L*×*S*)+*ε* (full model) and *Y* = *μ*+*L*+*R*(*L*)+*ε* (reduced model); where *μ* and *S* are defined above; *L*, the fixed effect of line, is the difference between the *P*–element insertion line and the co–isogenic control; *R* is the random effect of replicate vial; and *ε* is the environmental variance between individuals within each replicate vial. We computed the standardized effect of each mutation as *a*/*σ_P_*, where *a* is one–half the difference in mean life span between the homozygous mutant and the corresponding control line, and *σ_P_* is the phenotypic variation of the control line [64].

### *P*–element insertion sites

Bellen *et al.* [55] identified flanking sequences for the majority of lines using inverse PCR. We used the same technique to identify several more insertion sites. We isolated DNA from ∼25 individuals using the Puregene protocol, digested the DNA with *Hin*p1I and ligated it to obtain circular fragments containing both genomic and *P*–element DNA from both ends of the insert. We used PCR to amplify the 5′ end with oligonucleotide primers pGT1.5 (CCGCACGTAAGGGTTAATG) and pGT1.d (GAAGTTAAGCGTCTCCAGG) and the 3′ end with primers Pry1 (CCTTAGCATGTCCGTG–GGGTTTGAAT) and Pry4 (CAATCATATCGCTGTCTCACTCA), at annealing temperatures of 55°C. We sequenced the resulting products using (5′) Sp1 (ACACAACCTTTCCTCTCAA–CAA) and (3′) Spep1 (GACACTCAGAATACTATTC). We aligned the flanking sequences to the *D. melanogaster* genome using BLAST [68]. The inverse PCR protocol failed for lines BG00121, BG01700 and BG00817. For these lines, we determined the cytological location of the inserts by *in situ* hybridization to polytene chromosomes. We generated biotin-labeled probes using the BioNick Labeling System (Invitrogen) protocol, and used the Vectastain ABC kit (Vector Laboratories) for signal detection.

### Revertant alleles

We generated revertant lines for two chromosome *2* insert lines (BG00346, BG01042) and one chromosome *3* insert line (BG00043), using crossing schemes that preserved the co–isogenic background of the revertant lines. To construct the chromosome *2* revertant lines, we crossed *w* ^1118^; *P*; *iso3* females to *w* ^1118^; *CyO/Sp*; *SbΔ2–3/TM6,Tb* males. We mated male offspring of genotype *w* ^1118^; *CyO*/*P*; *SbΔ2–3/iso3* to *w* ^1118^; *CyO/Sp*; *iso3* females, and single male offspring of genotype *w* ^1118^; *CyO*/*P* ^−^; *iso3* in which the *P*–element had excised were crossed to *w* ^1118^; *CyO/Sp*; *iso3* females. In the following generation, males and females of genotype *w* ^1118^; *CyO*/*P* ^−^; *iso3* were mated *inter se* to make a homozygous revertant stock of genotype *w* ^1118^; *P* ^−^; *iso3*. To construct the chromosome *3* revertant lines, we crossed *w* ^1118^; *iso2*; *P* females to *w* ^1118^; *CyO/Sp*; *SbΔ2–3/TM6,Tb* males. We mated male offspring of genotype *w* ^1118^; *CyO*/*iso2*; *SbΔ2–3/P* to *w* ^1118^; *iso2*; *TM3*, *Sb/H* females, and single male offspring of genotype *w* ^1118^; *iso2*; *H/P* ^−^ were crossed to *w* ^1118^; *iso2*; *TM3*, *Sb/H* females. In the following generation, males and females of genotype *w* ^1118^; *iso2*; *TM3*, *Sb/P* ^−^ were mated inter se to make a homozygous revertant stock of genotype *w* ^1118^; *iso2*; *P* ^−^. Here *w* ^1118^, *iso2* and *iso3* are the three isogenic chromosomes of the *Canton S F* strain; *P* refers to the chromosome from the *Canton S F* strain with the *P*–element insertion associated with increased life span; and *P* ^−^ indicates a *P*–element excision allele.

We assessed the life span of the revertant lines simultaneously with the corresponding insert and control lines, with 12 replicates for each line and sex. The analysis of the BG00043 revertants was done in Raleigh under the same conditions described for the previous tests. The analysis of the BG00346 and BG01042 revertants was done in Moscow, Russia. We used the same ANOVA models described above for the second analysis of life span to assess differences in life span among the lines, and Tukey tests to identify significant differences between mutant, revertant and control lines.

### Epistasic interactions

We evaluated epistatic interactions among 10 mutations, generated in different genes in the F background, that had increased life span relative to the Canton S F strain (BG00004, BG00010, BG00028, BG00043, BG00297, BG00346, BG00495, BG00761, BG00817, BG01042). We crossed these lines in a half–diallel crossing scheme (excluding homozygous insert lines and reciprocal crosses) to create all 45 possible double heterozygote F_1_ genotypes following Griffing's [96] Method 4 and Model 1. We measured the life span of each F_1_ genotype as described above, with eight replicate vials per genotype per sex. The general combining ability (*GCA*) for each mutation is the difference between the mean life span of all genotypes containing that mutation and the overall mean [97]. We estimated *GCA* values as *GCA_i_* = [*T_i_*/(*n−*2)]−Σ*T*/*n*(*n*−2), where *T_i_* is the sum of mean life spans for all genotypes with the *i*th mutation, Σ*T* is twice the sum of mean life spans of all double–heterozygote genotypes, and *n* is the total number of mutant lines [64]. The specific combining ability (*SCA*) for any particular genotype is the difference between the mean life span of the genotype and the life span expected from the sum of the *GCA*s of the mutants involved in the cross [97]. We estimated *SCA* values as *SCA_ij_* = *X_ij_*−(*T_i_*+*T_j_*)/(*n*−2)+Σ*T*/(*n*−1)(*n*−2), where *X_ij_* is the mean life span of the offspring resulting from the cross of the *i*th and *j*th mutant lines. We also estimated *GCA*s and *SCA*s separately for each sex. We used Diallel–SAS05 [98] to estimate individual *GCA* and *SCA* effects and their standard errors; to perform ANOVAs to assess the significance of variation among the double heterozygous genotypes (*G*) for the full model pooled across sexes (*Y* = *μ*+*G*+*S*+*G*×*S*+*R*(*G*×*S*)+*ε*) and for the analyses of each sex separately (*Y* = *μ*+*G*+*R*(*G*)+*ε*); and to partition the *G* term into its *GCA* and *SCA* components, pooled across sexes (*Y* = *GCA*+*SCA*+*S*+*GCA*×*S*+*SCA*×*S*+*R*(*S*)+*ε*) and separately by sex (*Y* = *GCA*+*SCA*+*R*(*S*)+*ε*).

### Pleiotropic effects on organismal phenotypes

We assessed pleiotropic effects of mutations with significantly increased life span on stress resistance (chill coma recovery and starvation resistance) as well as a general measure of health (climbing ability) for virgin flies at one week and six weeks of age. We tested the F lines in three blocks, the A lines in two blocks, and all B lines simultaneously. Each block included the appropriate control line. We measured chill coma recovery time and climbing ability for individuals within each block within 48 hours, and scored all individuals within a block for starvation resistance at the same time.

#### Chill coma recovery

We transferred 30 same–sex individuals without anesthesia into empty vials and placed the vials in chambers containing melting ice. After three hours, we transferred the vials to room temperature, and placed the flies from each vial on their backs. We scored the time it took for each individual to stand on its legs in one minute intervals, for a maximum of 30 minutes for one week old flies and 45 minutes for six week old flies. The total sample size was 30 males and 30 females per line per age. We evaluated the significance of the difference in chill coma recovery time between each insert line and the control using ANOVAs pooled across sexes (*Y* = *μ*+*L*+*S*+*L*×*S*+*ε*) and for each sex separately(*Y* = *μ*+*L*+*ε*).

#### Starvation resistance

We assessed survival time of flies in vials containing non–nutritive medium (1.5% agar in 5 ml water). The sample size was 30 males and 30 females per line per age, with 10 same–sex flies in each of three replicate vials per sex per line. We recorded survival every eight hours until all flies were dead. We evaluated the significance of the difference in survival between each insert line and the control using ANOVAs pooled across sexes (*Y* = *μ*+*L*+*S*+*L*×*S*+*R*(*L*×*S*)+*ε*) and for each sex separately (*Y* = *μ*+*L*+*R*(*L*)+*ε*).

#### Climbing assay

We assessed the climbing ability of 30 flies per sex per line. We placed single flies in empty glass vials (15cm high×2.5cm diameter) without medium for one hour. We then tapped the flies to the bottom of the vial to stimulate a negative geotactic climbing response, and scored the height of the fly in the vial after 10 seconds (1 height unit = 5 mm). All climbing assays were conducted between 11:00 am and 12:30 pm. We evaluated the significance of the difference in climbing activity between each insert line and the control using ANOVAs pooled across sexes (*Y* = *μ*+*L*+*S*+*L*×*S*+*ε*) and for each sex separately(*Y* = *μ*+*L*+*ε*).

We also assessed variation for these three traits among all the long-lived mutant lines, expressed as deviations from controls, by similar two–factor mixed effects ANOVA pooled across sexes and for sexes separately, but treating the *L* term as a random effect. We computed cross–sex genetic correlations as described above. We estimated Pearson's product–moment correlations among line means, expressed as deviations from the control, to quantify directional pleiotropy among the traits.

### Whole-genome expression analysis

We chose seven *P*–element mutations in the Canton S F genetic background that were associated with increased life span in at least one sex, and for which we knew the exact *P*–element insertion sites (with the exception of BG00817) for whole genome expression analysis. These seven lines were a subset of the lines used for the epistasis analysis: BG00028, BG00043, BG00346, BG00495, BG00761, BG00817 and BG01042. We collected over 100 virgin flies of each sex over a 4–day interval from each of the *P*–element insert lines and the co–isogenic Canton S F control line, and maintained them as described for the previous life span assays. We froze 42 day–old flies, and created two replicate pools of 25 flies per sex per line for RNA extraction. We used a TRIZOL (Gibco BRL)/chloroform protocol to extract RNA from whole flies, and prepared cRNA from 5 µg RNA following the recommended protocol for eukaryotic one–cycle target labeling. We hybridized fragmented cRNA to Affymetrix *Drosophila* Genome 2.0 GeneChip arrays.

#### Analysis

For each of the 18,800 probe sets on the array there are 14 25mer perfect match (PM) oligonucleotides and 14 25mer single nucleotide mis–match (MM) pairs, with the mis–match at the 13^th^ base. We used the weighted log(PM–MM) intensity of each probe set as the quantitative measure of expression, and scaled the expression scores to a median intensity of 500. Each probe set is identified as being present, marginal or absent. After excluding probe sets that did not have a present signal for both replicates for at least one sex and line, we retained 12,635 probe sets for analysis. We performed ANOVAs of gene expression on each probe set using the model *Y* = *μ*+*L*+*S*+*L*×*S*+*ε*. We considered probe sets for which the *P*–value for the *L* term exceeded a *q*–value threshold of *q*<0.0001 [66] to be significant after correction for multiple tests. We also performed reduced ANOVAs for males and females separately for the probe sets with a significant *L*×*S* term. To identify in which mutant lines gene expression was different from the control line, we used Tukey tests on probe sets for which the *L* or *L*×*S* terms were significant, and full model ANOVAs comparing each individual line with the control. We used χ^2^ tests to evaluate over– and under–representation of Gene Ontology (GO) biological process categories for probe sets with a significant *L* effect for all mutant lines together (*q*<0.0001) as well as individual mutant lines (*q*<0.05) and individual sexes (*q*<0.001). We based the expected values on the ratio of the biological process probe sets in the significant lists to the total number of biological process probe sets on the array.

Data from all arrays used in the study are located at the Gene Expression Omnibus (GEO) public data repository (GSM216501–GSM216513, GSM216515–GSM216533).

### Statistics

We performed all statistical tests using SAS V8.2, V9.1 and Microsoft Office Excel. We used QVALUE software [66] to compute *q*–values.

## Supporting Information

Figure S1 Scatterplots of pleiotropic effects of *P*-element insertions associated with increased life span on starvation resistance, chill coma recovery, and climbing activity, in males at one week of age. All values are expressed as deviations from the control. (A) Life span and starvation stress. (B) Life span and chill coma recovery. (C) Life span and climbing activity. (D) Starvation resistance and chill coma recovery. (E) Starvation resistance and climbing activity. (F) Chill coma recovery and climbing activity.
(0.06 MB PDF)

Figure S2 Scatterplots of pleiotropic effects of *P*-element insertions associated with increased life span on starvation resistance, chill coma recovery and climbing activity, in females at one week of age. All values are expressed as deviations from the control. (A) Life span and starvation stress. (B) Life span and chill coma recovery. (C) Life span and climbing activity. (D) Starvation resistance and chill coma recovery. (E) Starvation resistance and climbing activity. (F) Chill coma recovery and climbing activity.
(0.06 MB PDF)

Figure S3 Scatterplots of pleiotropic effects of *P*-element insertions associated with increased life span on starvation resistance, chill coma recovery and climbing activity, in males at six weeks of age. All values are expressed as deviations from the control. (A) Life span and starvation stress. (B) Life span and chill coma recovery. (C) Life span and climbing activity. (D) Starvation resistance and chill coma recovery. (E) Starvation resistance and climbing activity. (F) Chill coma recovery and climbing activity.
(0.06 MB PDF)

Figure S4 Scatterplots of pleiotropic effects of *P*-element insertions associated with increased life span on starvation resistance, chill coma recovery and climbing activity, in females at six weeks of age. All values are expressed as deviations from the control. (A) Life span and starvation stress. (B) Life span and chill coma recovery. (C) Life span and climbing activity. (D) Starvation resistance and chill coma recovery. (E) Starvation resistance and climbing activity. (F) Chill coma recovery and climbing activity.
(0.06 MB PDF)

Table S1 Results of initial screen for effects of *P*-element insertions on life span. Cases in which the *P*-element insertion could possibly affect more than one gene (G1) are noted by listing the second (G2) and occasionally third (G3) gene in the region of the insertion. Mean life spans and life spans expressed as a deviation from the contemporaneous control are given for females, males and pooled across sexes. The 95, 99 and 99.9 confidence intervals (CI) are color coded for lines with increased (H) or decreased (L) life spans from the control line. The results from Dunnett's *t*-tests (D) are also color coded for lines with increased (H) or decreased (L) life spans from the control line.
(0.44 MB XLS)

Table S2 Diallel cross between ten *P{GT1}* insertion lines with increased life span. The table lists mean life span of double heterozygous genotypes and estimated *GCA* values for (A) sexes pooled; (B) females and (C) males. Parental homozygous *P{GT1}* insertion lines are indicated on the top row and first column of each panel. *T_i_* and *GCA* are defined in the text. Significant *GCA* values are indicated in bold font.
(0.07 MB DOC)

Table S3 Analyses of variance of life span of (A) double heterozygote genotypes and (B) general and specific combining abilities.
(0.05 MB DOC)

Table S4 Estimates of (A) general (*GCA*) and (B) specific (*SCA*) combining abilities.
(0.11 MB DOC)

Table S5 Pleiotropic effects of mutations increasing life span. (A) Starvation resistance (week 1); (B) Starvation resistance (week 6); (C) Chill coma recovery (week 1); (D) Chill coma recovery (week 6); (E) Climbing ability (week 1); (F) Climbing ability (week 6).
(0.54 MB DOC)

Table S6 Analyses of variance of pleiotropic effects of mutations with increased life span. (A) Starvation resistance; (B) Chill coma recovery; (C) Climbing ability.
(0.10 MB DOC)

Table S7 Statistical analysis of gene expression data. The results of ANOVAs of gene expression are given for each probe set declared to be present, for three separate analyses. Entries in the columns are *P*-values and *q*-values for the main effects of genotype (*G*), sex (*S*) and genotype by sex (*GS*). The first six columns refer to the analysis including all genotypes, pooled across sexes. The next four columns refer to the analysis including all genotypes, separately for males and females. The next 14 columns refer to the analysis comparing each mutant line to the control. The colors in each column highlight probe sets that are significant, with the darker colors denoting the more stringent significance threshold, accounting for multiple tests. For the analyses of each probe set compared to the control, blue denotes down-regulation and pink indicates up-regulation.
(4.49 MB XLS)

Table S8 Over-represented Biological Process Gene Ontology (GO) categories. (A) Females; (B) Males; (C) *pyd*; (D) *mub*; (E) *crol*; (F) *CG10990*; (G) *CG9238*; (H) BG00817; (I) *esg*.
(1.12 MB DOC)

Table S9 Probe sets in common to four or more mutations affecting increased life span. Red cells indicate probe sets with increased expression relative to the control, blue cells indicate probe sets with decreased expression relative to the control, and grey cells indicate probe cells with levels of expression not significantly different from the control.
(0.10 MB XLS)

Table S10 Candidate genes on the array.
(0.20 MB DOC)
