# Supplementary figures and images for: Quantitative and Molecular Genetic Analyses of Mutations Increasing Drosophila Life Span
Source: PLoS Genet. 2010 Jul 29;6(7):e1001037. doi: 10.1371/journal.pgen.1001037 (PMC2912381; doi:10.1371/journal.pgen.1001037)

**A**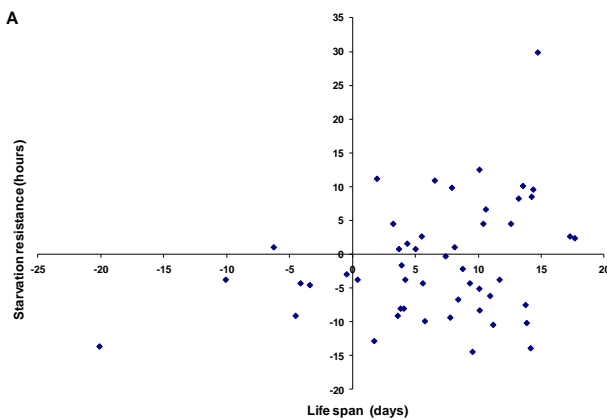**B**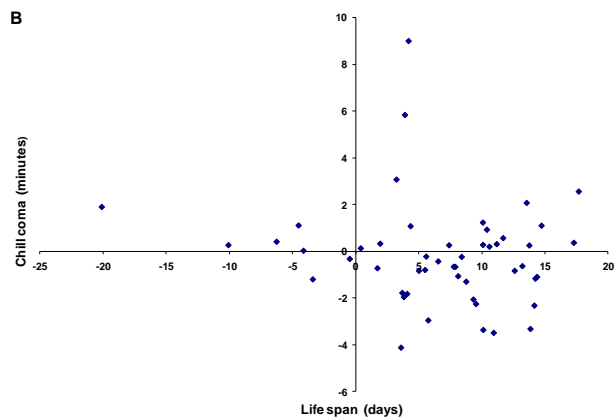**C**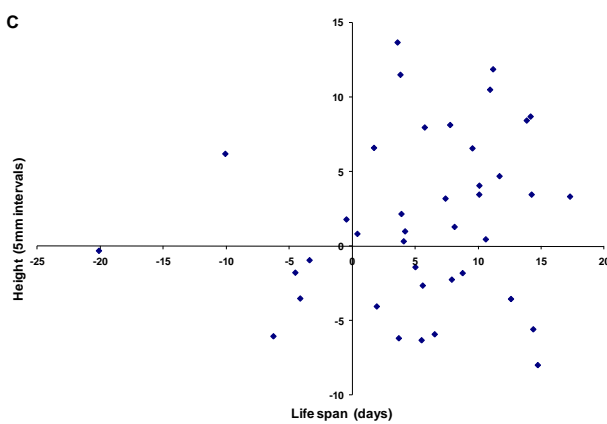**D**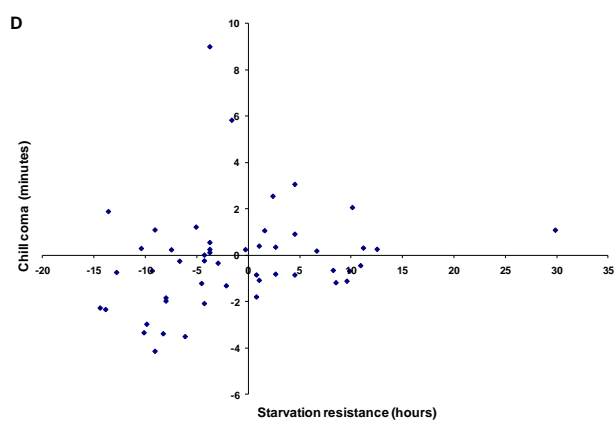**E**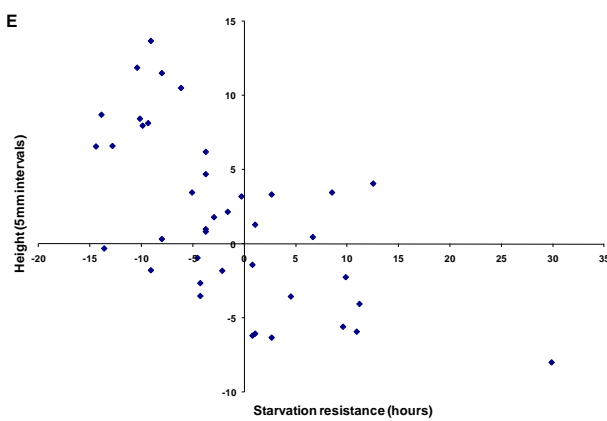**F**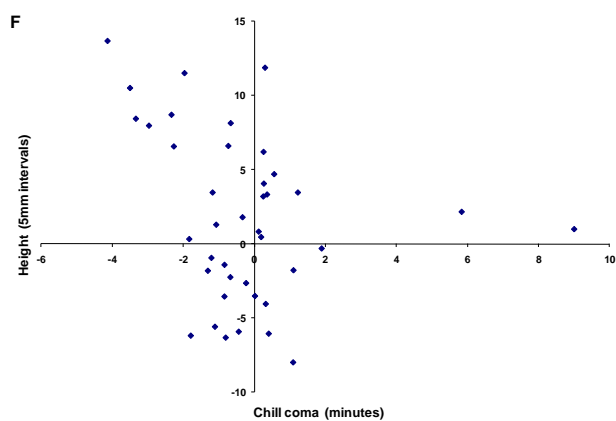

Supplement: Figure S1 — Scatterplots of pleiotropic effects of P-element insertions associated with increased life span on starvation resistance, chill coma recovery, and climbing activity, in males at one week of age. All values are expressed as deviations from the control. (A) Life span and starvation stress. (B) Life span and chill coma recovery. (C) Life span and climbing activity. (D) Starvation resistance and chill coma recovery. (E) Starvation resistance and climbing activity. (F) Chill coma recovery and climbing activity. (0.06 MB PDF) [file pgen.1001037.s001.pdf]

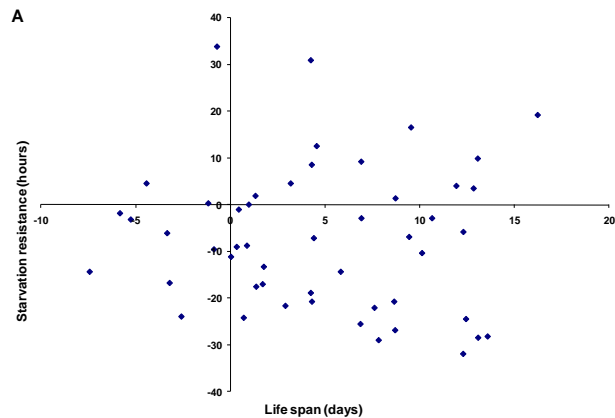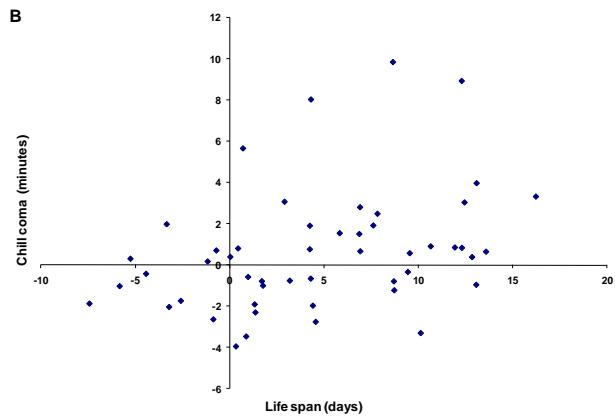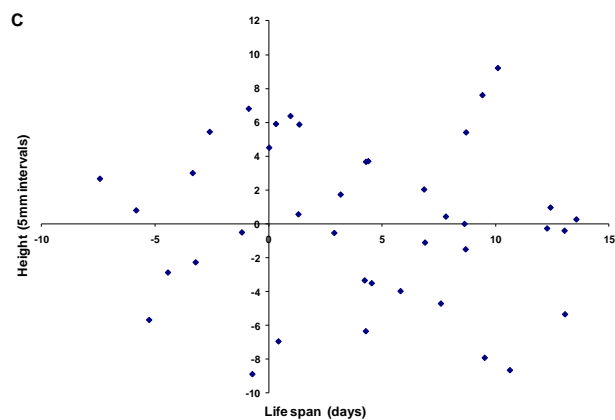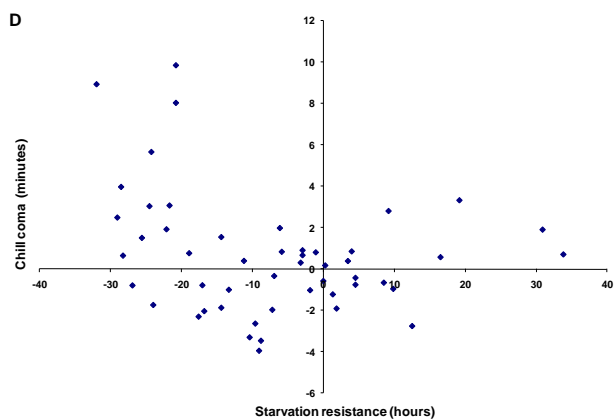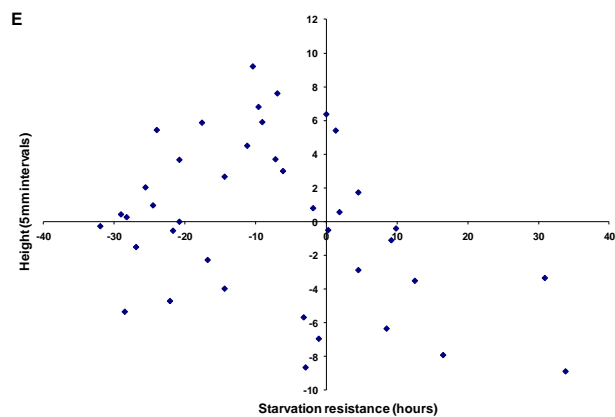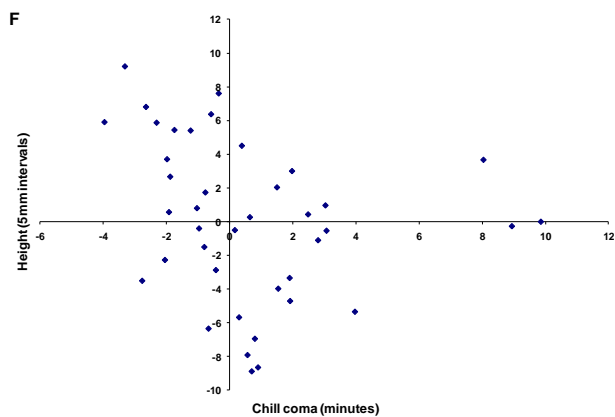

Supplement: Figure S2 — Scatterplots of pleiotropic effects of P-element insertions associated with increased life span on starvation resistance, chill coma recovery and climbing activity, in females at one week of age. All values are expressed as deviations from the control. (A) Life span and starvation stress. (B) Life span and chill coma recovery. (C) Life span and climbing activity. (D) Starvation resistance and chill coma recovery. (E) Starvation resistance and climbing activity. (F) Chill coma recovery and climbing activity. (0.06 MB PDF) [file pgen.1001037.s002.pdf]

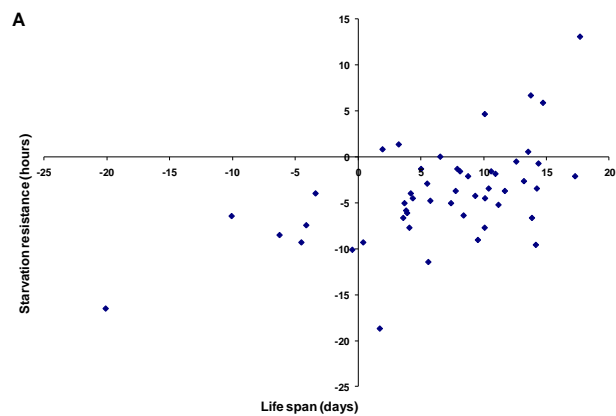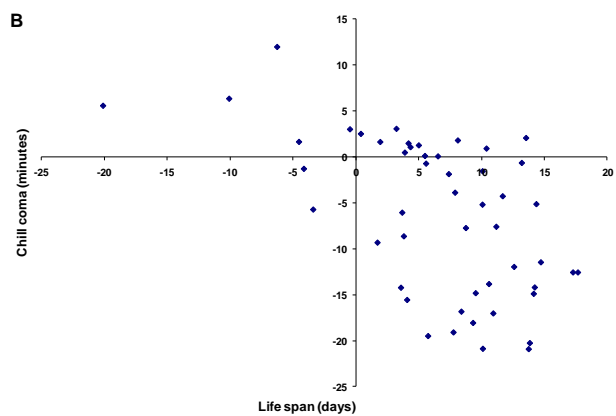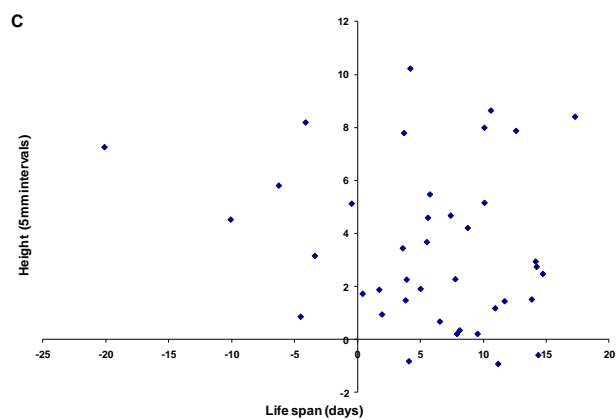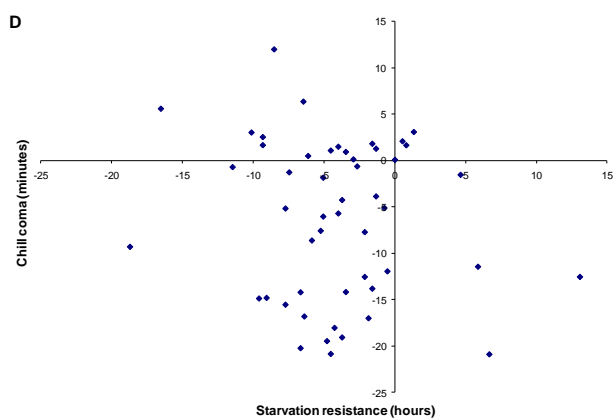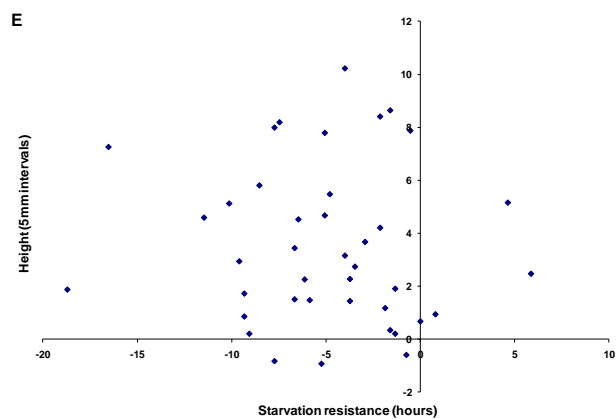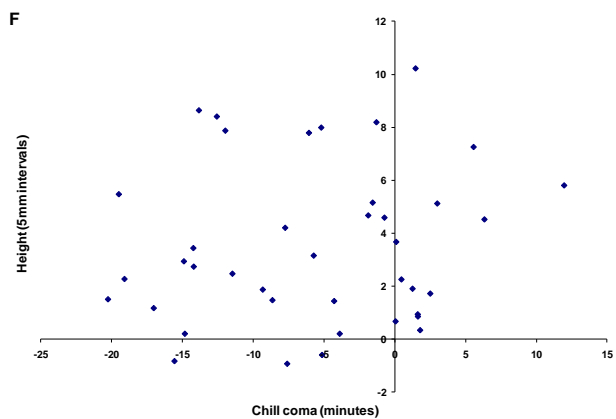

Supplement: Figure S3 — Scatterplots of pleiotropic effects of P-element insertions associated with increased life span on starvation resistance, chill coma recovery and climbing activity, in males at six weeks of age. All values are expressed as deviations from the control. (A) Life span and starvation stress. (B) Life span and chill coma recovery. (C) Life span and climbing activity. (D) Starvation resistance and chill coma recovery. (E) Starvation resistance and climbing activity. (F) Chill coma recovery and climbing activity. (0.06 MB PDF) [file pgen.1001037.s003.pdf]

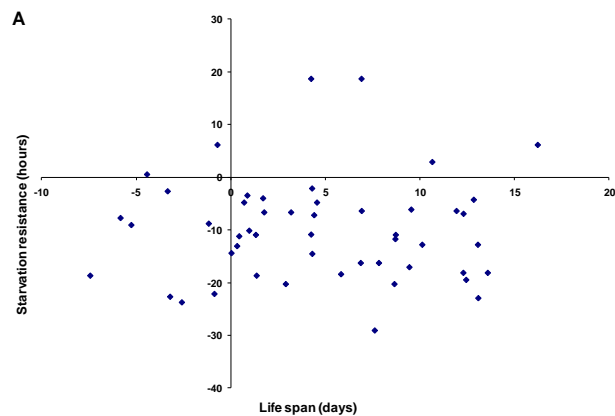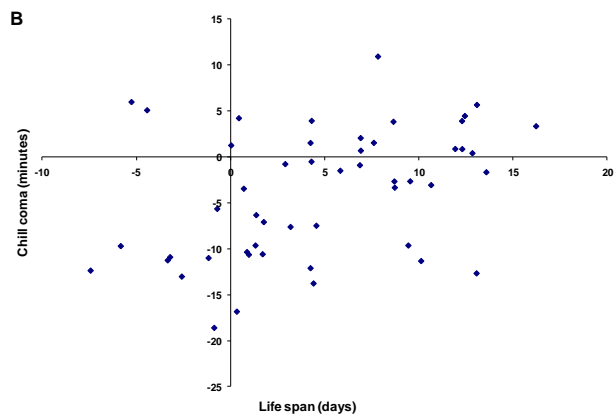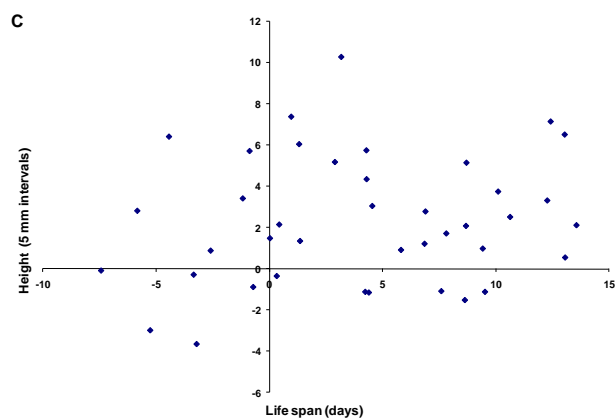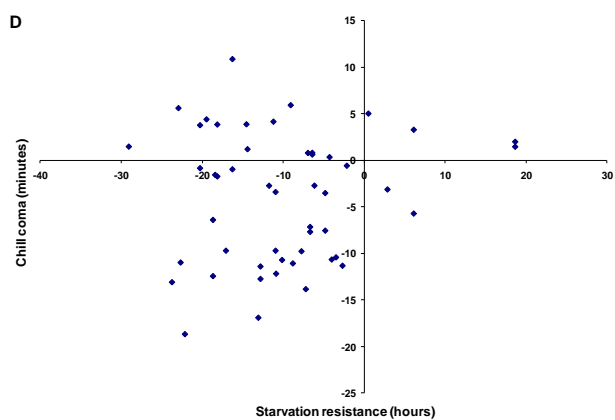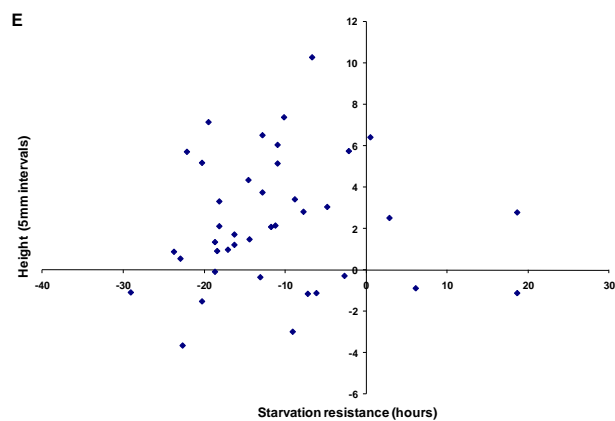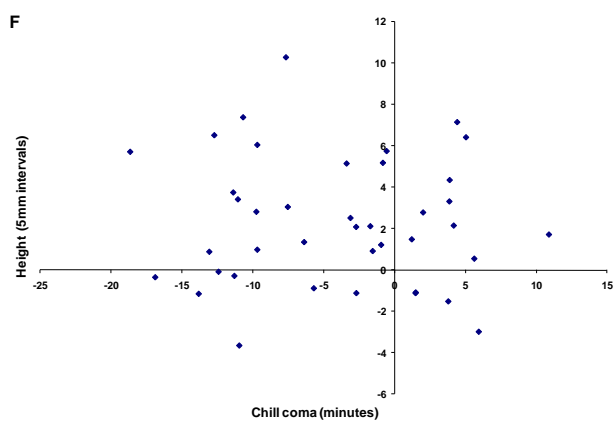

Supplement: Figure S4 — Scatterplots of pleiotropic effects of P-element insertions associated with increased life span on starvation resistance, chill coma recovery and climbing activity, in females at six weeks of age. All values are expressed as deviations from the control. (A) Life span and starvation stress. (B) Life span and chill coma recovery. (C) Life span and climbing activity. (D) Starvation resistance and chill coma recovery. (E) Starvation resistance and climbing activity. (F) Chill coma recovery and climbing activity. (0.06 MB PDF) [file pgen.1001037.s004.pdf]
