# Supplementary material for: Quantitative and Molecular Genetic Analyses of Mutations Increasing Drosophila Life Span
Source: PLoS Genet. 2010 Jul 29;6(7):e1001037. doi: 10.1371/journal.pgen.1001037 (PMC2912381; doi:10.1371/journal.pgen.1001037)
Supplement: Table S2 — Diallel cross between ten P{GT1} insertion lines with increased life span. The table lists mean life span of double heterozygous genotypes and estimated GCA values for (A) sexes pooled; (B) females and (C) males. Parental homozygous P{GT1} insertion lines are indicated on the top row and first column of each panel. Ti and GCA are defined in the text. Significant GCA values are indicated in bold font. (0.07 MB DOC) [file pgen.1001037.s006.doc]

**Table S2**

**Diallel cross between ten *P{GT1}* insertion lines with increased life span**

A. Pooled sexes

|  | **Male parent** | | | | | | | | | | |
| --- | --- | --- | --- | --- | --- | --- | --- | --- | --- | --- | --- |
| **Female parent** | **BG00817** | **BG00004** | ***CG9238*** | ***esg*** | ***CG31531*** | ***pyd*** | ***mub*** | **BG00297** | ***crol*** | ***T_i_*** | ***GCA*** |
| ***CG10990*** | 69.39 | 72.48 | 72.45 | 71.31 | 73.48 | 70.36 | 69.01 | 65.44 | 70.05 | 633.97 | –0.6638 |
| **BG00817** |  | 73.81 | 72.39 | 72.49 | 72.32 | 73.12 | 73.73 | 72.30 | 72.57 | 652.11 | **1.5670** |
| **BG00004** |  |  | 73.46 | 71.35 | 74.85 | 72.51 | 69.95 | 64.72 | 70.40 | 643.52 | 0.4812 |
| ***CG9238*** |  |  |  | 69.59 | 68.87 | 72.33 | 73.76 | 68.61 | 70.15 | 641.62 | 0.2563 |
| ***esg*** |  |  |  |  | 72.81 | 68.74 | 68.39 | 70.89 | 68.09 | 633.66 | –0.7269 |
| ***CG31531*** |  |  |  |  |  | 70.87 | 71.16 | 73.15 | 74.35 | 651.87 | **1.5870** |
| ***pyd*** |  |  |  |  |  |  | 70.28 | 67.72 | 74.06 | 640.00 | 0.0561 |
| ***mub*** |  |  |  |  |  |  |  | 70.27 | 68.54 | 635.10 | –0.5655 |
| **BG00297** |  |  |  |  |  |  |  |  | 70.99 | 624.09 | **–1.9396** |
| ***crol*** |  |  |  |  |  |  |  |  |  | 639.22 | –0.0518 |

**B. Females**

|  | **Male parent** | | | | | | | | | | |
| --- | --- | --- | --- | --- | --- | --- | --- | --- | --- | --- | --- |
| **Female parent** | **BG00817** | **BG00004** | ***CG9238*** | ***esg*** | ***CG31531*** | ***pyd*** | ***mub*** | **BG00297** | ***crol*** | ***T_i_*** | ***GCA*** |
| ***CG10990*** | 68.45 | 71.90 | 79.18 | 69.98 | 75.00 | 70.69 | 68.80 | 67.38 | 65.25 | 636.62 | 0.5955 |
| **BG00817** |  | 70.28 | 75.58 | 73.33 | 73.50 | 72.00 | 72.92 | 68.85 | 67.95 | 642.85 | 1.2635 |
| **BG00004** |  |  | 82.48 | 70.77 | 78.28 | 73.20 | 71.76 | 63.80 | 70.30 | 652.76 | **2.4923** |
| ***CG9238*** |  |  |  | 69.31 | 67.71 | 71.50 | 74.02 | 71.02 | 68.18 | 658.98 | **3.2779** |
| ***esg*** |  |  |  |  | 70.18 | 63.54 | 61.31 | 65.38 | 62.68 | 606.47 | **–3.2864** |
| ***CG31531*** |  |  |  |  |  | 68.31 | 69.50 | 70.03 | 71.75 | 644.26 | **1.5548** |
| ***pyd*** |  |  |  |  |  |  | 70.84 | 65.08 | 73.68 | 628.84 | –0.4742 |
| ***mub*** |  |  |  |  |  |  |  | 70.56 | 66.44 | 626.16 | –0.8261 |
| **BG00297** |  |  |  |  |  |  |  |  | 70.22 | 612.31 | **–2.5550** |
| ***crol*** |  |  |  |  |  |  |  |  |  | 616.44 | **–2.0422** |

**C. Males**

|  | **Male parent** | | | | | | | | | | |
| --- | --- | --- | --- | --- | --- | --- | --- | --- | --- | --- | --- |
| **Female parent** | **BG00817** | **BG00004** | ***CG9238*** | ***esg*** | ***CG31531*** | ***pyd*** | ***mub*** | **BG00297** | ***crol*** | ***T_i_*** | ***GCA*** |
| ***CG10990*** | 70.35 | 73.05 | 66.05 | 72.65 | 72.74 | 70.03 | 69.23 | 63.50 | 74.97 | 632.58 | **–1.8068** |
| **BG00817** |  | 77.62 | 69.21 | 71.64 | 71.10 | 74.24 | 74.54 | 75.84 | 77.08 | 661.62 | **1.8424** |
| **BG00004** |  |  | 64.21 | 71.92 | 71.41 | 71.82 | 68.22 | 65.68 | 70.51 | 634.46 | **–1.5666** |
| ***CG9238*** |  |  |  | 69.91 | 69.98 | 73.18 | 73.49 | 66.20 | 72.18 | 624.40 | **–2.8056** |
| ***esg*** |  |  |  |  | 75.51 | 74.23 | 76.06 | 76.54 | 73.37 | 661.82 | **1.9000** |
| ***CG31531*** |  |  |  |  |  | 73.50 | 72.87 | 76.20 | 76.82 | 660.13 | **1.6623** |
| ***pyd*** |  |  |  |  |  |  | 69.75 | 70.38 | 74.44 | 651.54 | 0.5674 |
| ***mub*** |  |  |  |  |  |  |  | 69.97 | 70.60 | 644.73 | –0.2794 |
| **BG00297** |  |  |  |  |  |  |  |  | 71.77 | 636.08 | **–1.3586** |
| ***crol*** |  |  |  |  |  |  |  |  |  | 661.73 | **1.8449** |

The table lists mean life span of double heterozygous genotypes and estimated *GCA* values for (A) sexes pooled; (B) females and (C) males. Parental homozygous *P{GT1}* insertion lines are indicated on the top row and first column of each panel. *T_i_* and *GCA* are defined in the text. Significant *GCA* values are indicated in bold font.
