# Supplementary material for: Quantitative and Molecular Genetic Analyses of Mutations Increasing Drosophila Life Span
Source: PLoS Genet. 2010 Jul 29;6(7):e1001037. doi: 10.1371/journal.pgen.1001037 (PMC2912381; doi:10.1371/journal.pgen.1001037)
Supplement: Table S3 — Analyses of variance of life span of (A) double heterozygote genotypes and (B) general and specific combining abilities. (0.05 MB DOC) [file pgen.1001037.s007.doc]

**Table S3**

**A. Analyses of variance of life span of double heterozygote genotypes**

| **Analysis** | **Source** | **d.f.** | **SS** | ***F*** | ***P*** |
| --- | --- | --- | --- | --- | --- |
| **Pooled Sexes** | Genotype | 44 | 18338.5 | 5.70 | < 0.0001 |
|  | Sex | 1 | 2335.2 | 31.94 | < 0.0001 |
|  | Genotype × Sex | 44 | 32427.4 | 10.08 | < 0.0001 |
|  | Rep (Genotype × Sex) | 78 | 259583.8 | 45.51 | < 0.0001 |
|  | Error | 3316 | 242467.4 | ― | ― |
| **Females** | Genotype | 44 | 30021.3 | 7.06 | < 0.0001 |
|  | Rep (Sex) | 39 | 152482.6 | 40.48 | < 0.0001 |
|  | Error | 1655 | 15986.5 | ― | ― |
| **Males** | Genotype | 44 | 20712.2 | 9.47 | < 0.0001 |
|  | Rep (Sex) | 39 | 107101.2 | 55.22 | < 0.0001 |
|  | Error | 1661 | 82606.9 | ― | ― |

**B. Analyses of variance of general and specific combining abilities**

| **Analysis** | **Source** | **d.f.** | **SS** | ***F*** | ***P*** |
| --- | --- | --- | --- | --- | --- |
| **Pooled Sexes** | *GCA* | 9 | 6427.9 | 9.76 | < 0.0001 |
|  | *SCA* | 35 | 11770.5 | 4.60 | < 0.0001 |
|  | Sex | 1 | 2335.2 | 31.94 | < 0.0001 |
|  | *GCA* × Sex | 9 | 16099.2 | 24.46 | < 0.0001 |
|  | *SCA* × Sex | 35 | 15465.5 | 6.04 | < 0.0001 |
|  | Rep(Sex) | 78 | 259583.8 | 45.51 | < 0.0001 |
|  | Error | 3316 | 242467.4 | ― | ― |
| **Females** | *GCA* | 9 | 13511.7 | 15.54 | < 0.0001 |
|  | *SCA* | 35 | 16556.0 | 4.90 | < 0.0001 |
|  | Rep | 39 | 152482.6 | 40.48 | < 0.0001 |
|  | Error | 1655 | 15986.5 | ― | ― |
| **Males** | *GCA* | 9 | 9015.4 | 20.14 | < 0.0001 |
|  | *SCA* | 35 | 10680.1 | 6.14 | < 0.0001 |
|  | Rep | 39 | 107101.2 | 55.22 | < 0.0001 |
|  | Error | 1661 | 82606.9 | ― | ― |
