# Supplementary material for: Quantitative and Molecular Genetic Analyses of Mutations Increasing Drosophila Life Span
Source: PLoS Genet. 2010 Jul 29;6(7):e1001037. doi: 10.1371/journal.pgen.1001037 (PMC2912381; doi:10.1371/journal.pgen.1001037)
Supplement: Table S4 — Estimates of (A) general (GCA) and (B) specific (SCA) combining abilities. (0.11 MB DOC) [file pgen.1001037.s008.doc]

**Table S4**

**Estimates of (A) general (*GCA*) and (B) specific (*SCA*) combining abilities**

**A**

|  |  | **Sexes Pooled** | | **Females** | | **Males** | |
| --- | --- | --- | --- | --- | --- | --- | --- |
| **Line** | **Gene** | ***GCA*** | ***P*** | ***GCA*** | ***P*** | ***GCA*** | ***P*** |
| BG00495 | *CG10990* | –0.6638 | 0.1641 | 0.5955 | 0.4325 | –**1.8068** | **0.0012** |
| BG00817 | BG00817 | **1.5670** | **0.0009** | 1.2635 | 0.084 | **1.8424** | **0.0011** |
| BG00004 | BG00004 | 0.4812 | 0.3059 | **2.4923** | **0.0006** | –**1.5666** | **0.0051** |
| BG00761 | *CG9238* | 0.2563 | 0.5867 | **3.2779** | **<0.0001** | –**2.8056** | **<0.0001** |
| BG01042 | *esg* | –0.7269 | 0.1254 | –**3.2864** | **<0.0001** | **1.9000** | **0.0008** |
| BG00010 | *CG31531* | **1.5870** | **0.0009** | **1.5548** | **0.0418** | **1.6623** | **0.0031** |
| BG00028 | *pyd* | 0.0561 | 0.9053 | –0.4742 | 0.5155 | 0.5674 | 0.3109 |
| BG00043 | *mub* | –0.5655 | 0.2304 | –0.8261 | 0.2569 | –0.2794 | 0.6177 |
| BG00297 | BG00297 | –**1.9396** | **<.0001** | –**2.5550** | **0.0004** | –**1.3586** | **0.0151** |
| BG00346 | *crol* | –0.0518 | 0.9127 | –**2.0422** | **0.0053** | **1.8449** | **0.0010** |

Significant *GCA* values are indicated in bold font

**B**

| Parents | | Sexes pooled | | Females | | Males | |
| --- | --- | --- | --- | --- | --- | --- | --- |
| Female | Male | *SCA* | *P* | *SCA* | *P* | *SCA* | *P* |
| *CG10990* | BG00817 | **–2.6385** | **0.0362** | **–**3.8020 | 0.0505 | **–**1.5620 | 0.2978 |
| *CG10990* | BG00004 | 1.5840 | 0.1971 | **–**1.4972 | 0.4317 | **4.5304** | **0.0019** |
| *CG10990* | *CG9238* | 1.7647 | 0.1548 | **4.9736** | **0.0105** | **–**1.2306 | 0.3978 |
| *CG10990* | *esg* | 1.6296 | 0.1851 | 2.3565 | 0.2164 | 0.6638 | 0.6488 |
| *CG10990* | *CG31531* | 1.9008 | 0.1730 | 3.5255 | 0.1733 | 0.9725 | 0.5081 |
| *CG10990* | *pyd* | **–**0.0433 | 0.9722 | 0.3098 | 0.8720 | **–**0.5479 | 0.7092 |
| *CG10990* | *mub* | **–**0.8485 | 0.4918 | **–**1.2787 | 0.5021 | **–**0.6191 | 0.6734 |
| *CG10990* | BG00297 | **–3.0326** | **0.0136** | **–**0.9749 | 0.6086 | **–5.2276** | **0.0003** |
| *CG10990* | *crol* | **–**0.3162 | 0.7979 | **–**3.6126 | 0.0582 | **3.0207** | **0.0399** |
| BG00817 | BG00004 | 0.6810 | 0.5842 | **–3.7901** | **0.0455** | **5.4464** | **0.0003** |
| BG00817 | *CG9238* | **–**0.4150 | 0.7402 | 0.7992 | 0.6791 | **–**1.6181 | 0.2762 |
| BG00817 | *esg* | 0.5964 | 0.6305 | **5.0580** | **0.0083** | **–3.9553** | **0.0073** |
| BG00817 | *CG31531* | **–**1.8877 | 0.1267 | 0.3724 | 0.8453 | **–4.2355** | **0.0040** |
| BG00817 | *pyd* | 0.4603 | 0.7130 | 0.9369 | 0.6278 | **–**0.0279 | 0.9850 |
| BG00817 | *mub* | 1.6460 | 0.1841 | 2.1875 | 0.2530 | 1.0394 | 0.4798 |
| BG00817 | BG00297 | 1.5871 | 0.2022 | **–**0.1482 | 0.9382 | **3.3979** | **0.0222** |
| BG00817 | *crol* | **–**0.0297 | 0.981 | **–**1.6136 | 0.4040 | 1.5152 | 0.3030 |
| BG00004 | *CG9238* | 1.6761 | 0.1737 | **6.3954** | **0.0007** | **–3.2356** | **0.0278** |
| BG00004 | *esg* | 0.5017 | 0.6840 | 1.2157 | 0.5249 | **–**0.3014 | 0.8361 |
| BG00004 | *CG31531* | 1.7028 | 0.1699 | **3.9120** | **0.0421** | **–**0.5600 | 0.7031 |
| BG00004 | *pyd* | 0.9017 | 0.4621 | 0.8725 | 0.6449 | 0.9312 | 0.5223 |
| BG00004 | *mub* | **–**1.0340 | 0.4035 | **–**0.2022 | 0.9166 | **–**1.8220 | 0.2107 |
| BG00004 | BG00297 | **–4.8906** | **<.0001** | **–6.4467** | **0.0007** | **–3.2667** | **0.0278** |
| BG00004 | *crol* | **–**1.1228 | 0.3622 | **–**0.4594 | 0.8084 | **–**1.7222 | 0.2412 |
| *CG9238* | *esg* | **–**0.9705 | 0.4427 | **–**0.9697 | 0.6122 | **–**1.0160 | 0.5078 |
| *CG9238* | *CG31531* | **–4.0712** | **0.0010** | **–7.4721** | **0.0001** | **–**0.7747 | 0.5947 |
| *CG9238* | *pyd* | 0.9181 | 0.4563 | **–**1.6130 | 0.3945 | **3.4610** | **0.0186** |
| *CG9238* | *mub* | **2.9702** | **0.0160** | 1.2639 | 0.5046 | **4.6155** | **0.0017** |
| *CG9238* | BG00297 | **–**0.7777 | 0.5258 | **–**0.0072 | 0.9969 | **–**1.5288 | 0.2937 |
| *CG9238* | *crol* | **–**1.0947 | 0.3745 | **–**3.3700 | 0.0755 | 1.3273 | 0.3666 |
| *esg* | *CG31531* | 0.8908 | 0.4798 | 1.5912 | 0.4132 | 0.1123 | 0.9404 |
| *esg* | *pyd* | **–**1.6157 | 0.1968 | **–**2.9622 | 0.1217 | **–**0.0987 | 0.9476 |
| *esg* | *mub* | **–**1.3070 | 0.2988 | **–4.8410** | **0.0115** | 2.6844 | 0.0770 |
| *esg* | BG00297 | **2.5025** | **0.0424** | 0.9071 | 0.6319 | **4.1636** | **0.0047** |
| *esg* | *crol* | **–**2.2278 | 0.0767 | **–**2.3558 | 0.2278 | **–**2.2526 | 0.1297 |
| *CG31531* | *pyd* | **–**1.8490 | 0.1383 | **–**3.0835 | 0.1094 | **–**0.6269 | 0.6728 |
| *CG31531* | *mub* | **–**0.9071 | 0.4627 | **–**1.5380 | 0.4200 | **–**0.3445 | 0.8147 |
| *CG31531* | BG00297 | **2.4624** | **0.0461** | 0.7646 | 0.6910 | **4.0033** | **0.0060** |
| *CG31531* | *crol* | 1.7581 | 0.1651 | 1.9280 | 0.3314 | 1.4536 | 0.3275 |
| *pyd* | *mub* | **–**0.3082 | 0.8034 | 1.7926 | 0.3534 | **–**2.4309 | 0.0951 |
| *pyd* | BG00297 | **–**1.4650 | 0.2321 | **–**2.2051 | 0.2442 | **–**0.7268 | 0.6175 |
| *pyd* | *crol* | **3.0011** | **0.0159** | **5.9521** | **0.0021** | 0.0670 | 0.9637 |
| *mub* | BG00297 | 1.6853 | 0.1754 | 3.5976 | 0.0599 | **–**0.2644 | 0.8586 |
| *mub* | *crol* | **–**1.8966 | 0.1240 | **–**0.9816 | 0.6080 | **–2.8584** | **0.0497** |
| BG00297 | *crol* | 1.9286 | 0.1176 | **4.5129** | **0.0173** | **–**0.5505 | 0.7079 |

Significant *SCA* values are indicated in bold font
