# Supplementary material for: Quantitative and Molecular Genetic Analyses of Mutations Increasing Drosophila Life Span
Source: PLoS Genet. 2010 Jul 29;6(7):e1001037. doi: 10.1371/journal.pgen.1001037 (PMC2912381; doi:10.1371/journal.pgen.1001037)
Supplement: Table S5 — Pleiotropic effects of mutations increasing life span. (A) Starvation resistance (week 1); (B) Starvation resistance (week 6); (C) Chill coma recovery (week 1); (D) Chill coma recovery (week 6); (E) Climbing ability (week 1); (F) Climbing ability (week 6). (0.54 MB DOC) [file pgen.1001037.s009.doc]

**Table S5**

**Pleiotropic effects of mutations increasing life span**

1. **S**tarvation resistance (week 1)

| **Line** | **Mutational effects** | | | | | | ***P*-values from ANOVA** | | | | |
| --- | --- | --- | --- | --- | --- | --- | --- | --- | --- | --- | --- |
|  | **Mean (%^a^)** | | | ***a/σ*_p_^b^** | | | **Sexes Pooled^c^** | | | **Sexes Separate** | |
|  | **♂,♀** | **♂** | **♀** | **♂,♀** | **♂** | **♀** | ***S*** | ***L*** | ***L*×*S*** | ***L*♂** | ***L*♀** |
| BG00004 (F) | 36.7 (-0.4) | 30.9 (-6.5) | 42.4 (4.6) | -0.01 | -0.12 | 0.09 | **** | ns | ns | ns | ns |
| BG00008 (F) | 34.0 (-24.1) | 31.7 (-24.7) | 36.3 (-23.6) | -0.38 | -1.02 | -0.29 | * | **** | ns | **** | * |
| BG00010 (F) | 41.2 (12.0) | 29.3 (-11.3) | 53.1 (30.9) | 0.21 | -0.21 | 0.60 | **** | * | **** | ns† | **** |
| BG00028 (F) | 68.7 (86.6) | 62.9 (90.3) | 74.4 (83.6) | 1.53 | 1.64 | 1.61 | **** | **** | ns | **** | **** |
| BG00037 (F) | 34.9 (-22.0) | 29.3 (-30.4) | 40.5 (-14.6) | -0.35 | -1.25 | -0.18 | *** | **** | ns | **** | ns |
| BG00041 (F) | 35.5 (-17.9) | 29.3 (-24.1) | 41.6 (-12.8) | -0.30 | -0.70 | -0.20 | **** | **** | ns | **** | ns† |
| BG00042 (F) | 32.3 (-25.3) | 41.1 (6.2) | 23.5 (-50.8) | -0.43 | 0.18 | -0.78 | ns† | **** | **** | ns | **** |
| BG00043 (F) | 49.9 (35.5) | 42.7 (29.0) | 57.1 (40.8) | 0.63 | 0.53 | 0.79 | **** | **** | ns† | **** | **** |
| BG00080 (B) | 41.1 (-23.0) | 38.1 (-8.9) | 44.0 (-32.1) | -0.42 | -0.32 | -0.91 | **** | **** | **** | * | **** |
| BG00121 (F) | 35.1 (-21.7) | 32.3 (-23.4) | 37.9 (-20.2) | -0.34 | -0.96 | -0.25 | * | **** | ns | **** | * |
| BG00297 (F) | 41.3 (12.3) | 37.6 (13.7) | 45.1 (11.2) | 0.22 | 0.25 | 0.22 | **** | * | ns | * | ns |
| BG00336 (B) | 38.0 (-28.8) | 36.8 (-12.1) | 39.2 (-39.5) | -0.52 | -0.44 | -1.12 | **** | **** | **** | ** | **** |
| BG00346 (F) | 41.1 (11.6) | 41.6 (25.8) | 40.5 (0.0) | 0.20 | 0.47 | 0.00 | ns† | * | * | *** | ns |
| BG00472 (F) | 28.8 (-35.7) | 27.7 (-34.2) | 29.9 (-37.1) | -0.56 | -1.41 | -0.46 | ns† | **** | ns | **** | *** |
| BG00495 (F) | 43.1 (17.0) | 35.7 (8.1) | 50.4 (24.3) | 0.30 | 0.15 | 0.47 | **** | ** | ns† | ns | ** |
| BG00528 (B) | 39.2 (-26.5) | 38.1 (-8.9) | 40.3 (-37.9) | -0.48 | -0.32 | -1.08 | **** | **** | **** | * | **** |
| BG00757 (F) | 25.9 (-42.3) | 28.3 (-32.9) | 23.5 (-50.6) | -0.67 | -1.35 | -0.62 | ns | **** | * | **** | **** |
| BG00761 (F) | 37.3 (1.4) | 32.8 (-0.8) | 41.9 (3.3) | 0.03 | -0.01 | 0.06 | **** | ns | ns | ns | ns |
| BG00767 (B) | 36.5 (-31.5) | 40.3 (-3.8) | 32.8 (-49.4) | -0.57 | -0.14 | -1.41 | **** | **** | **** | ns | **** |
| BG00817 (F) | 41.9 (13.8) | 42.9 (29.8) | 40.8 (0.7) | 0.24 | 0.54 | 0.01 | ns | ** | * | **** | ns |
| BG00864 (B) | 36.9 (-30.8) | 38.1 (-8.9) | 35.7 (-44.9) | -0.56 | -0.32 | -1.28 | **** | **** | **** | * | **** |
| BG00890 (F) | 30.5 (-29.3) | 30.4 (-21.4) | 30.7 (-35.8) | -0.50 | -0.62 | -0.55 | ** | **** | * | **** | **** |
| BG00907 (F) | 35.2 (-21.4) | 32.0 (-24.1) | 38.4 (-19.1) | -0.34 | -0.99 | -0.24 | ** | **** | ns | **** | * |
| BG00915 (F) | 30.0 (-30.6) | 31.2 (-19.3) | 28.8 (-39.7) | -0.52 | -0.56 | -0.60 | ns | **** | * | *** | **** |
| BG01004 (F) | 36.7 (-15.1) | 34.4 (-11.0) | 38.9 (-18.4) | -0.26 | -0.32 | -0.28 | *** | ** | ns | * | * |
| BG01030 (A) | 47.7 (33.1) | 38.7 (13.3) | 56.8 (51.1) | 0.55 | 0.28 | 0.74 | **** | **** | *** | * | **** |
| BG01031 (A) | 32.8 (-3.1) | 36.0 (3.1) | 29.6 (-9.8) | -0.06 | 0.14 | -0.13 | ** | ns | ns | ns | ns |
| BG01042 (F) | 39.2 (6.5) | 39.7 (20.2) | 38.7 (-4.6) | 0.11 | 0.37 | -0.09 | ns† | ns | * | ** | ns |
| BG01121 (F) | 32.4 (-27.7) | 34.1 (-19.0) | 30.7 (-35.4) | -0.44 | -0.78 | -0.44 | ns | **** | * | **** | *** |
| BG01345 (A) | 37.1 (3.3) | 42.4 (24.2) | 31.7 (-15.6) | 0.06 | 0.51 | -0.23 | ns† | ns | *** | *** | ns† |
| BG01403 (A) | 38.7 (7.8) | 35.7 (4.7) | 41.6 (10.6) | 0.13 | 0.10 | 0.15 | * | ns | ns | ns | ns |
| BG01540 (B) | 37.6 (-29.5) | 37.3 (-10.8) | 37.9 (-41.6) | -0.54 | -0.39 | -1.18 | **** | **** | **** | * | **** |
| BG01550 (F) | 33.6 (-25.0) | 34.1 (-19.0) | 33.1 (-30.3) | -0.39 | -0.78 | -0.37 | ns | **** | ns | **** | ** |
| BG01553 (F) | 33.2 (-23.1) | 32.0 (-17.2) | 34.4 (-27.9) | -0.39 | -0.50 | -0.43 | ** | **** | ns† | *** | **** |
| BG01615 (A) | 39.5 (10.0) | 44.3 (29.7) | 34.7 (-7.8) | 0.17 | 0.62 | -0.11 | ns | ns† | ** | **** | ns |
| BG01677 (A) | 39.9 (11.2) | 38.7 (13.3) | 41.1 (9.2) | 0.18 | 0.28 | 0.13 | ns | * | ns | * | ns |
| BG01700 (F) | 38.1 (-14.9) | 36.0 (-14.6) | 40.3 (-15.2) | -0.23 | -0.60 | -0.19 | * | ** | ns | **** | ns† |
| BG01701 (F) | 35.1 (-21.7) | 33.1 (-21.5) | 37.1 (-21.9) | -0.34 | -0.89 | -0.27 | * | **** | ns | **** | ** |
| BG01702 (A) | 36.5 (7.9) | 35.7 (2.3) | 37.3 (13.8) | 0.14 | 0.10 | 0.18 | ns | ns† | ns | ns | ns |
| BG01710 (A) | 54.9 (62.2) | 46.1 (32.1) | 63.7 (94.3) | 1.14 | 1.43 | 1.24 | **** | **** | **** | **** | **** |
| BG01878 (B) | 37.1 (-30.5) | 37.6 (-10.2) | 36.5 (-43.6) | -0.56 | -0.37 | -1.24 | **** | **** | **** | * | **** |
| BG01918 (A) | 34.7 (2.4) | 37.6 (7.6) | 31.7 (-3.3) | 0.04 | 0.34 | -0.04 | * | ns | ns | * | ns |
| BG01950 (B) | 36.1 (-32.3) | 28.3 (-32.5) | 44.0 (-32.1) | -0.59 | -1.17 | -0.91 | **** | **** | * | **** | **** |
| BG01976 (B) | 39.6 (-25.8) | 42.9 (2.5) | 36.3 (-44.0) | -0.47 | 0.09 | -1.25 | **** | **** | **** | ns | **** |
| BG02019 (B) | 41.7 (-27.2) | 40.3 (-18.4) | 43.2 (-33.9) | -0.64 | -0.76 | -0.94 | **** | **** | *** | **** | **** |
| BG02039 (A) | 43.6 (28.7) | 45.9 (31.3) | 41.3 (26.0) | 0.53 | 1.39 | 0.34 | ns† | **** | ns | **** | * |
| BG02049 (B) | 52.3 (-2.0) | 42.7 (1.9) | 61.9 (-4.5) | -0.04 | 0.07 | -0.13 | **** | ns | ns | ns | ns |
| BG02128 (B) | 44.7 (-16.3) | 38.9 (-7.0) | 50.4 (-22.2) | -0.30 | -0.25 | -0.63 | **** | **** | ** | ns† | **** |
| BG02395 (B) | 40.4 (-24.3) | 37.6 (-10.2) | 43.1 (-33.5) | -0.44 | -0.37 | -0.95 | **** | **** | **** | * | **** |
| BG02644 (B) | 63.9 (19.7) | 54.4 (29.9) | 74.0 (14.2) | 0.36 | 1.08 | 0.40 | **** | **** | ns | **** | * |

Letters in parenthesis after the Line name denote different co-isogenic *Canton S* host strains for *P{GT1}*-element insertion.  ^a^ Percent deviation from the mean life span of the control line. ^b^ standardized mutational effect (see text for explanation), ^c^ *S* and *L* denote the main cross-classified effects of Sex and Line, respectively in the ANOVA of life span. ns *P* > 0.1, † 0.05 < *P* < 0.1, * *P* < 0.05, ** *P* < 0.01,*** *P* < 0.001, **** *P* < 0.0001.

1. **S**tarvation resistance (week 6)

| **Line** | **Mutational effects** | | | | | | ***P*-values from ANOVA** | | | | |
| --- | --- | --- | --- | --- | --- | --- | --- | --- | --- | --- | --- |
|  | **Mean (%^a^)** | | | ***a/σ*_p_^b^** | | | **Sexes Pooled^c^** | | | **Sexes Separate** | |
|  | **♂,♀** | **♂** | **♀** | **♂,♀** | **♂** | **♀** | ***S*** | ***L*** | ***L*×*S*** | ***L*♂** | ***L*♀** |
| BG00004 (F) | 26.5 (-19.8) | 23.5 (-8.3) | 29.6 (-27.0) | -0.32 | -0.20 | -0.65 | **** | **** | ** | ns† | **** |
| BG00008 (F) | 28.7 (-23.5) | 23.6 (-18.2) | 31.7 (-31.2) | -0.27 | -0.40 | -0.38 | **** | **** | * | ** | *** |
| BG00010 (F) | 28.8 (-12.9) | 21.9 (-14.6) | 35.7 (-11.8) | -0.21 | -0.35 | -0.29 | **** | *** | ns | ** | * |
| BG00028 (F) | 39.1 (18.1) | 31.5 (22.9) | 46.7 (15.1) | 0.29 | 0.55 | 0.37 | **** | *** | ns | *** | * |
| BG00037 (F) | 20.8 (-44.4) | 10.1 (-65.0) | 29.1 (-37.0) | -0.50 | -1.44 | -0.45 | **** | **** | ns | **** | *** |
| BG00041 (F) | 29.6 (-9.8) | 25.1 (-13.0) | 34.1 (-7.2) | -0.14 | -0.29 | -0.10 | **** | ns† | ns | * | ns |
| BG00042 (F) | 36.9 (12.6) | 41.9 (45.4) | 32.0 (-13.0) | 0.18 | 1.00 | -0.17 | ns | ns† | *** | **** | ns |
| BG00043 (F) | 29.8 (-9.9) | 24.9 (-2.9) | 34.4 (-15.1) | -0.16 | -0.07 | -0.37 | **** | * | ns† | ns | * |
| BG00080 (B) | 31.7 (-22.6) | 26.1 (-13.3) | 37.2 (-28.1) | -0.32 | -0.40 | -0.60 | **** | **** | *** | ** | **** |
| BG00121 (F) | 24.0 (-35.9) | 24.0 (-16.7) | 24.0 (-48.0) | -0.41 | -0.37 | -0.59 | **** | **** | **** | *** | **** |
| BG00297 (F) | 29.5 (-10.9) | 25.1 (-2.1) | 33.9 (-16.4) | -0.18 | -0.05 | -0.40 | **** | * | ns† | ns | * |
| BG00336 (B) | 28.9 (-29.3) | 22.4 (-25.7) | 35.5 (-31.4) | -0.42 | -0.77 | -0.67 | **** | **** | ** | **** | **** |
| BG00346 (F) | 26.3 (-20.6) | 22.1 (-13.5) | 30.4 (-25.0) | -0.33 | -0.33 | -0.60 | **** | **** | * | ** | *** |
| BG00472 (F) | 23.6 (-37.0) | 19.7 (-31.5) | 27.5 (-40.5) | -0.42 | -0.70 | -0.49 | **** | **** | * | **** | **** |
| BG00495 (F) | 25.6 (-22.6) | 23.5 (-8.3) | 27.7 (-31.6) | -0.36 | -0.20 | -0.76 | **** | **** | **** | ns† | **** |
| BG00528 (B) | 26.5 (-35.2) | 20.8 (-31.0) | 32.3 (-37.6) | -0.50 | -0.93 | -0.80 | **** | **** | *** | **** | **** |
| BG00757 (F) | 20.8 (-44.5) | 19.2 (-33.3) | 22.4 (-51.4) | -0.51 | -0.74 | -0.63 | **** | **** | *** | **** | **** |
| BG00761 (F) | 25.1 (-24.2) | 20.5 (-19.8) | 29.6 (-27.0) | -0.39 | -0.48 | -0.65 | **** | **** | * | *** | **** |
| BG00767 (B) | 29.2 (-28.6) | 24.0 (-20.4) | 33.6 (-35.1) | -0.41 | -0.61 | -0.74 | **** | **** | ** | **** | **** |
| BG00817 (F) | 28.0 (-15.3) | 24.3 (-5.2) | 31.7 (-21.7) | -0.25 | -0.13 | -0.52 | **** | ** | * | ns | ** |
| BG00864 (B) | 30.2 (-26.2) | 23.7 (-21.5) | 35.5 (-31.4) | -0.37 | -0.65 | -0.67 | **** | **** | ** | **** | **** |
| BG00890 (F) | 28.5 (-13.0) | 24.3 (-15.7) | 32.8 (-10.9) | -0.19 | -0.35 | -0.14 | **** | * | ns | * | ns |
| BG00907 (F) | 27.6 (-26.3) | 22.1 (-23.1) | 33.1 (-28.3) | -0.30 | -0.51 | -0.35 | **** | **** | ns | **** | ** |
| BG00915 (F) | 30.8 (-6.2) | 35.5 (23.1) | 25.9 (-29.5) | -0.09 | 0.51 | -0.39 | ns | ns | **** | *** | ** |
| BG01004 (F) | 28.9 (-11.8) | 24.5 (-14.8) | 33.3 (-9.4) | -0.17 | -0.33 | -0.12 | **** | * | ns | ** | ns |
| BG01030 (A) | 35.3 (11.8) | 26.7 (5.3) | 44.0 (16.2) | 0.14 | 0.13 | 0.20 | **** | ns† | ns | ns | ns |
| BG01031 (A) | 24.3 (-18.0) | 21.3 (-7.0) | 27.2 (-25.0) | -0.26 | -0.23 | -0.42 | **** | **** | ** | ns | *** |
| BG01042 (F) | 28.4 (-14.1) | 24.0 (-6.3) | 32.8 (-19.1) | -0.23 | -0.15 | -0.46 | **** | *** | * | ns | ** |
| BG01121 (F) | 22.3 (-40.6) | 21.1 (-26.9) | 23.5 (-49.1) | -0.46 | -0.59 | -0.60 | **** | **** | *** | **** | **** |
| BG01345 (A) | 26.8 (-15.2) | 22.7 (-10.5) | 30.9 (-18.3) | -0.18 | -0.26 | -0.22 | **** | * | ns | * | ns† |
| BG01403 (A) | 26.1 (-17.3) | 20.8 (-17.9) | 31.5 (-16.9) | -0.21 | -0.44 | -0.21 | **** | ** | ns | *** | ns† |
| BG01540 (B) | 33.1 (-19.2) | 26.1 (-13.3) | 40.0 (-22.7) | -0.28 | -0.40 | -0.48 | **** | **** | *** | ** | **** |
| BG01550 (F) | 25.2 (-32.7) | 22.9 (-20.4) | 27.5 (-40.5) | -0.37 | -0.45 | -0.49 | **** | **** | ** | **** | **** |
| BG01553 (F) | 26.3 (-19.9) | 22.4 (-22.2) | 30.1 (-18.1) | -0.28 | -0.49 | -0.24 | **** | *** | ns | **** | * |
| BG01615 (A) | 28.7 (-9.3) | 25.9 (2.1) | 31.5 (-16.9) | -0.11 | 0.05 | -0.21 | **** | ns† | ns† | ns | ns† |
| BG01677 (A) | 27.7 (-12.2) | 21.9 (-13.7) | 33.6 (-11.3) | -0.15 | -0.33 | -0.14 | **** | * | ns | ** | ns |
| BG01700 (F) | 32.9 (-12.1) | 26.9 (-6.5) | 38.9 (-15.6) | -0.14 | -0.14 | -0.19 | **** | * | ns | ns | ns† |
| BG01701 (F) | 27.7 (-26.0) | 22.1 (-23.1) | 33.3 (-27.7) | -0.29 | -0.51 | -0.34 | **** | **** | ns | **** | ** |
| BG01702 (A) | 29.2 (-1.4) | 21.6 (-5.8) | 36.8 (1.5) | -0.02 | -0.19 | 0.02 | **** | ns | ns | ns | ns |
| BG01710 (A) | 39.3 (32.9) | 23.7 (3.5) | 54.9 (51.5) | 0.47 | 0.12 | 0.86 | **** | **** | **** | ns | **** |
| BG01878 (B) | 28.1 (-31.3) | 22.7 (-24.8) | 33.6 (-35.1) | -0.45 | -0.75 | -0.74 | **** | **** | *** | **** | **** |
| BG01918 (A) | 22.5 (-23.9) | 20.0 (-12.8) | 25.1 (-30.9) | -0.34 | -0.42 | -0.52 | **** | **** | ** | ** | **** |
| BG01950 (B) | 27.0 (-34.0) | 13.6 (-54.9) | 31.5 (-39.2) | -0.49 | -1.65 | -0.83 | **** | **** | ns | **** | **** |
| BG01976 (B) | 25.9 (-36.7) | 21.6 (-28.3) | 28.8 (-44.3) | -0.53 | -0.85 | -0.94 | **** | **** | **** | **** | **** |
| BG02019 (B) | 28.7 (-40.1) | 20.8 (-31.0) | 36.5 (-44.3) | -0.47 | -0.93 | -1.13 | **** | **** | **** | **** | **** |
| BG02039 (A) | 28.5 (-3.6) | 22.9 (0.0) | 34.1 (-5.9) | -0.05 | 0.00 | -0.10 | **** | ns | ns | ns | ns |
| BG02049 (B) | 39.6 (-3.3) | 25.1 (-16.8) | 54.6 (5.6) | -0.05 | -0.51 | 0.12 | **** | ns | * | *** | ns |
| BG02128 (B) | 26.7 (-34.9) | 20.0 (-33.6) | 33.3 (-35.6) | -0.50 | -1.01 | -0.75 | **** | **** | ** | **** | **** |
| BG02395 (B) | 25.1 (-38.8) | 18.7 (-38.1) | 31.5 (-39.2) | -0.56 | -1.14 | -0.83 | **** | **** | ** | **** | **** |
| BG02644 (B) | 50.3 (22.8) | 34.8 (15.4) | 70.4 (36.1) | 0.33 | 0.46 | 0.76 | **** | **** | *** | * | **** |

Letters in parenthesis after the Line name denote different co-isogenic *Canton S* host strains for *P{GT1}*-element insertion.  ^a^ Percent deviation from the mean life span of the control line. ^b^ standardized mutational effect (see text for explanation), ^c^ *S* and *L* denote the main cross-classified effects of Sex and Line, respectively in the ANOVA of life span. ns *P* > 0.1, † 0.05 < *P* < 0.1, * *P* < 0.05, ** *P* < 0.01,*** *P* < 0.001, **** *P* < 0.0001.

1. **C**hill coma recovery (week 1)

| **Line** | **Mutational effects** | | | | | | ***P*-values from ANOVA** | | | | |
| --- | --- | --- | --- | --- | --- | --- | --- | --- | --- | --- | --- |
|  | **Mean (%^a^)** | | | ***a/σ*_p_^b^** | | | **Sexes Pooled^c^** | | | **Sexes Separate** | |
|  | **♂,♀** | **♂** | **♀** | **♂,♀** | **♂** | **♀** | ***S*** | ***L*** | ***L*×*S*** | ***L*♂** | ***L*♀** |
| BG00004 (F) | 9.4 ( -14.3) | 10.3 (-11.2) | 8.4 ( -18.5) | -0.27 | -0.26 | -0.30 | ** | ** | ns | ns† | * |
| BG00008 (F) | 12.7 (2.6) | 13.7 (2.2) | 11.8 (3.4) | 0.07 | 0.06 | 0.09 | **** | ns | ns | ns | ns |
| BG00010 (F) | 9.9 ( -9.9) | 12.2 (4.8) | 7.6 ( -26.7) | -0.18 | 0.11 | -0.43 | **** | * | *** | ns | **** |
| BG00028 (F) | 11.9 (8.4) | 12.7 (9.4) | 11.1 (6.8) | 0.16 | 0.21 | 0.11 | * | ns | ns | ns | ns |
| BG00037 (F) | 11.9 (-4.4) | 12.7 (-5.5) | 11.0 (-3.0) | -0.11 | -0.15 | -0.08 | **** | ns | ns | ns | ns |
| BG00041 (F) | 13.0 (5.2) | 12.7 (-5.0) | 13.4 (17.4) | 0.13 | -0.14 | 0.46 | ns | ns | ** | ns | ** |
| BG00042 (F) | 16.0 (34.9) | 15.0 (20.5) | 16.9 (50.2) | 0.86 | 0.64 | 1.08 | ns | **** | * | ** | **** |
| BG00043 (F) | 10.7 (-2.3) | 10.5 (-9.5) | 10.9 (5.5) | -0.04 | -0.22 | 0.09 | ns | ns | ns | ns | ns |
| BG00080 (B) | 18.6 (84.6) | 20.5 (78.3) | 16.7 (93.1) | 2.14 | 2.95 | 3.30 | *** | **** | ns | **** | **** |
| BG00121 (F) | 9.6 ( -22.8) | 10.4 (-22.1) | 8.7 ( -23.3) | -0.57 | -0.61 | -0.62 | *** | **** | ns | *** | **** |
| BG00297 (F) | 10.2 (-7.1) | 10.8 (-7.2) | 9.6 ( -7.4) | -0.13 | -0.16 | -0.12 | * | ns | ns | ns | ns |
| BG00336 (B) | 11.4 (13.3) | 12.7 (10.6) | 10.1 (17.4) | 0.34 | 0.40 | 0.62 | **** | *** | ns | * | ** |
| BG00346 (F) | 10.1 (-7.9) | 10.5 (-10.1) | 9.8 ( -5.6) | -0.15 | -0.23 | -0.09 | ns | ns | ns | ns | ns |
| BG00472 (F) | 10.1 (-18.4) | 11.1 (-16.9) | 9.1 ( -20.3) | -0.46 | -0.47 | -0.54 | **** | **** | ns | **** | **** |
| BG00495 (F) | 10.7 (-2.6) | 12.0 (3.1) | 9.4 ( -9.3) | -0.05 | 0.07 | -0.15 | *** | ns | ns | ns | ns |
| BG00528 (B) | 11.6 (15.7) | 11.6 (1.0) | 11.7 (35.1) | 0.40 | 0.04 | 1.25 | **** | **** | **** | ns | **** |
| BG00757 (F) | 10.4 (-16.6) | 11.1 (-17.4) | 9.6 ( -15.3) | -0.41 | -0.48 | -0.41 | ** | *** | ns | * | * |
| BG00761 (F) | 10.5 (-4.7) | 11.9 (2.1) | 9.1 ( -11.9) | -0.09 | 0.05 | -0.19 | ** | ns | ns | ns | ns |
| BG00767 (B) | 17.5 (73.3) | 17.3 (50.7) | 17.6 (103.5) | 1.85 | 1.91 | 3.67 | ** | **** | ** | **** | **** |
| BG00817 (F) | 10.8 (-2.1) | 11.0 (-5.8) | 10.5 (1.6) | -0.04 | -0.13 | 0.03 | ns | ns | ns | ns | ns |
| BG00864 (B) | 11.4 (13.6) | 11.8 (2.2) | 11.1 (28.8) | 0.34 | 0.08 | 1.02 | *** | ** | * | ns | *** |
| BG00890 (F) | 9.8 ( -17.4) | 9.1 ( -27.1) | 10.5 (-7.0) | -0.43 | -0.85 | -0.15 | ns | **** | ** | **** | ns |
| BG00907 (F) | 8.7 ( -29.5) | 10.1 (-24.9) | 7.4 ( -34.8) | -0.74 | -0.69 | -0.93 | **** | **** | ns | **** | **** |
| BG00915 (F) | 12.4 (4.3) | 12.7 (1.9) | 12.0 (6.7) | 0.11 | 0.06 | 0.14 | * | ns | ns | ns | ns |
| BG01004 (F) | 9.0 ( -23.8) | 10.4 (-16.6) | 7.8 ( -30.8) | -0.59 | -0.52 | -0.66 | **** | **** | ns† | *** | **** |
| BG01030 (A) | 11.6 (37.5) | 12.5 (32.4) | 10.7 (44.8) | 0.79 | 1.09 | 0.83 | **** | **** | ns | **** | **** |
| BG01031 (A) | 9.1 ( -4.3) | 9.7 ( -10.0) | 8.5 ( 3.7) | -0.09 | -0.27 | 0.10 | **** | ns | * | ** | ns |
| BG01042 (F) | 10.6 (-3.6) | 11.8 (1.6) | 9.3 ( -10.0) | -0.07 | 0.04 | -0.16 | **** | ns | ns | ns | ns |
| BG01121 (F) | 10.5 (-15.8) | 11.6 (-13.7) | 9.3 ( -18.0) | -0.39 | -0.38 | -0.48 | **** | **** | ns | ** | ** |
| BG01345 (A) | 8.5 ( 0.8) | 8.8 ( -6.8) | 8.2 ( 11.2) | 0.02 | -0.23 | 0.21 | **** | ns | * | ns | ns |
| BG01403 (A) | 9.5 ( 11.8) | 10.5 (11.3) | 8.3 ( 11.4) | 0.25 | 0.38 | 0.21 | **** | ** | ns | ** | ns |
| BG01540 (B) | 9.1 ( -9.8) | 10.3 (-10.5) | 7.8 ( -9.3) | -0.25 | -0.40 | -0.33 | **** | *** | ns | ** | * |
| BG01550 (F) | 10.5 (-15.6) | 11.4 (-14.7) | 9.5 ( -16.5) | -0.39 | -0.40 | -0.44 | **** | **** | ns | ** | ** |
| BG01553 (F) | 11.3 (-5.1) | 12.2 (-2.0) | 10.3 (-9.0) | -0.13 | -0.06 | -0.19 | ** | ns | ns | ns | ns |
| BG01615 (A) | 9.9 ( 17.0) | 11.5 (21.8) | 8.1 ( 8.9) | 0.36 | 0.73 | 0.16 | **** | *** | ns† | *** | ns |
| BG01677 (A) | 9.0 ( 6.6) | 10.4 (9.7) | 7.8 ( 5.1) | 0.14 | 0.33 | 0.09 | **** | ns† | ns | ns† | ns |
| BG01700 (F) | 9.7 ( -22.2) | 9.9 ( -26.1) | 9.4 ( -17.4) | -0.55 | -0.72 | -0.47 | ** | **** | * | **** | *** |
| BG01701 (F) | 8.7 ( -30.1) | 9.3 ( -30.8) | 8.1 ( -29.1) | -0.75 | -0.85 | -0.78 | **** | **** | ns | **** | **** |
| BG01702 (A) | 8.8 ( -6.9) | 9.9 ( -7.8) | 7.7 ( -5.3) | -0.15 | -0.21 | -0.15 | **** | ns† | ns | ns | ns |
| BG01710 (A) | 10.6 (11.6) | 11.1 (3.0) | 10.1 (23.3) | 0.26 | 0.08 | 0.65 | **** | ** | * | ns | *** |
| BG01878 (B) | 10.4 (3.6) | 11.5 (0.1) | 9.3 ( 7.4) | 0.09 | 0.01 | 0.26 | **** | ns | ns | ns | ns |
| BG01918 (A) | 9.5 ( -0.3) | 10.0 (-7.5) | 9.0 ( 9.8) | -0.01 | -0.21 | 0.27 | **** | ns | ns† | ns | ns |
| BG01950 (B) | 16.1 (59.6) | 13.4 (16.5) | 18.5 (114.1) | 1.51 | 0.62 | 4.05 | * | **** | **** | *** | **** |
| BG01976 (B) | 12.2 (21.6) | 11.9 (3.5) | 12.6 (45.9) | 0.55 | 0.13 | 1.63 | ** | **** | **** | ns | **** |
| BG02019 (B) | 11.6 (14.8) | 12.6 (9.6) | 10.5 (22.2) | 0.37 | 0.36 | 0.79 | **** | **** | ns | * | *** |
| BG02039 (A) | 9.0 ( -5.5) | 10.3 (-4.1) | 7.5 ( -8.2) | -0.12 | -0.11 | -0.23 | **** | ns† | ns | ns | ns† |
| BG02049 (B) | 9.6 ( -4.4) | 9.7 ( -15.6) | 9.5 ( 10.4) | -0.11 | -0.59 | 0.37 | **** | ns | **** | **** | ns† |
| BG02128 (B) | 10.7 (6.1) | 11.2 (-2.9) | 10.2 (17.8) | 0.15 | -0.11 | 0.63 | **** | * | *** | ns | *** |
| BG02395 (B) | 11.5 (14.1) | 11.3 (-2.0) | 11.7 (35.5) | 0.36 | -0.08 | 1.26 | ** | ** | *** | ns | **** |
| BG02644 (B) | 11.6 (15.2) | 11.8 (2.3) | 11.4 (32.4) | 0.39 | 0.09 | 1.15 | **** | **** | *** | ns | **** |

Letters in parenthesis after the Line name denote different co-isogenic *Canton S* host strains for *P{GT1}*-element insertion.  ^a^ Percent deviation from the mean life span of the control line. ^b^ standardized mutational effect (see text for explanation), ^c^ *S* and *L* denote the main cross-classified effects of Sex and Line, respectively in the ANOVA of life span. ns *P* > 0.1, † 0.05 < *P* < 0.1, * *P* < 0.05, ** *P* < 0.01,*** *P* < 0.001, **** *P* < 0.0001.

1. **Chill** coma recovery (week 6)

| **Line** | **Mutational effects** | | | | | | ***P*-values from ANOVA** | | | | |
| --- | --- | --- | --- | --- | --- | --- | --- | --- | --- | --- | --- |
|  | **Mean (%^a^)** | | | ***a/σ*_p_^b^** | | | **Sexes Pooled^c^** | | | **Sexes Separate** | |
|  | **♂,♀** | **♂** | **♀** | **♂,♀** | **♂** | **♀** | ***S*** | ***L*** | ***L*×*S*** | ***L*♂** | ***L*♀** |
| BG00004 (F) | 18.7 (-31.2) | 20.9 (-27.0) | 16.6 (-36.8) | -0.46 | -0.46 | -0.50 | * | **** | ns | ** | **** |
| BG00008 (F) | 31.1 (-8.8) | 29.8 (-20.3) | 32.7 (3.9) | -0.15 | -0.45 | 0.06 | ns | ns | * | ** | ns |
| BG00010 (F) | 21.6 (-20.7) | 24.4 (-14.9) | 18.8 (-28.6) | -0.31 | -0.25 | -0.39 | * | ** | ns | ns | ** |
| BG00028 (F) | 18.9 (-30.7) | 17.2 (-40.0) | 20.6 (-21.6) | -0.46 | -0.68 | -0.30 | ns | **** | * | **** | * |
| BG00037 (F) | 24.7 (-27.8) | 28.0 (-24.9) | 21.8 (-30.7) | -0.48 | -0.55 | -0.47 | *** | **** | ns | ** | **** |
| BG00041 (F) | 19.1 (-44.1) | 18.3 (-51.1) | 20.2 (-35.8) | -0.76 | -1.13 | -0.55 | ns | **** | * | **** | **** |
| BG00042 (F) | 28.1 (-22.4) | 29.2 (-30.1) | 27.0 (-11.4) | -0.43 | -1.35 | -0.18 | **** | **** | *** | **** | ns† |
| BG00043 (F) | 23.6 (-13.4) | 23.5 (-17.9) | 23.6 (-10.2) | -0.20 | -0.31 | -0.14 | ns | * | ns | ns† | ns |
| BG00080 (B) | 19.7 (16.6) | 20.0 (8.0) | 19.3 (25.4) | 0.21 | 0.09 | 0.39 | ns | ns† | ns | ns | * |
| BG00121 (F) | 15.4 (-55.0) | 17.9 (-52.2) | 12.9 (-59.2) | -0.95 | -1.15 | -0.91 | *** | **** | ns | **** | **** |
| BG00297 (F) | 17.6 (-35.1) | 16.7 (-41.8) | 18.7 (-29.1) | -0.52 | -0.71 | -0.40 | ns | **** | ns | **** | ** |
| BG00336 (B) | 13.9 (-17.7) | 13.3 (-28.0) | 14.5 (-6.0) | -0.22 | -0.32 | -0.09 | ns | ** | * | ** | ns |
| BG00346 (F) | 15.0 (-44.7) | 14.4 (-49.6) | 15.6 (-40.6) | -0.67 | -0.84 | -0.55 | ns | **** | ns | **** | **** |
| BG00472 (F) | 23.8 (-30.3) | 22.5 (-39.7) | 25.1 (-20.2) | -0.53 | -0.87 | -0.31 | ns | **** | ** | **** | * |
| BG00495 (F) | 14.8 (-45.5) | 16.1 (-43.9) | 13.6 (-48.3) | -0.68 | -0.75 | -0.66 | ns† | **** | ns | **** | **** |
| BG00528 (B) | 20.4 (20.9) | 21.0 (13.6) | 19.8 (28.8) | 0.26 | 0.15 | 0.44 | ns† | ** | ns | ns | ** |
| BG00757 (F) | 20.5 (-40.0) | 22.5 (-39.9) | 18.4 (-41.4) | -0.69 | -0.88 | -0.64 | ** | **** | ns | **** | **** |
| BG00761 (F) | 24.9 (-8.7) | 26.8 (-6.5) | 22.9 (-12.8) | -0.13 | -0.11 | -0.18 | ns | ns | ns | ns | ns |
| BG00767 (B) | 19.1 (13.4) | 19.0 (2.6) | 19.3 (25.2) | 0.17 | 0.03 | 0.39 | ns | ns | ns | ns | * |
| BG00817 (F) | 19.8 (-27.1) | 24.8 (-13.6) | 15.3 (-42.0) | -0.40 | -0.23 | -0.57 | *** | **** | * | ns | **** |
| BG00864 (B) | 25.6 (51.7) | 24.9 (34.3) | 26.3 (70.9) | 0.64 | 0.39 | 1.09 | ns | **** | ns | ** | **** |
| BG00890 (F) | 20.4 (-43.8) | 20.9 (-50.0) | 19.9 (-34.8) | -0.85 | -2.25 | -0.56 | **** | **** | *** | **** | **** |
| BG00907 (F) | 15.8 (-53.6) | 17.1 (-54.2) | 14.6 (-53.5) | -0.93 | -1.19 | -0.82 | ** | **** | ns | **** | **** |
| BG00915 (F) | 19.4 (-46.5) | 20.9 (-50.1) | 18.4 (-39.8) | -0.90 | -2.25 | -0.64 | **** | **** | ** | **** | **** |
| BG01004 (F) | 21.8 (-39.8) | 23.7 (-43.2) | 20.1 (-34.0) | -0.77 | -1.94 | -0.54 | **** | **** | * | **** | **** |
| BG01030 (A) | 19.51 (24.2) | 19.76 (11.8) | 19.27 (41.6) | 0.43 | 0.21 | 1.26 | * | **** | ns | ns | **** |
| BG01031 (A) | 16.9 (30.2) | 16.1 (12.6) | 17.7 (50.9) | 0.49 | 0.19 | 1.21 | ns | **** | ** | ns | **** |
| BG01042 (F) | 15.7 (-42.3) | 14.8 (-48.3) | 16.6 (-37.0) | -0.63 | -0.82 | -0.51 | ns | **** | ns | **** | **** |
| BG01121 (F) | 21.2 (-38.1) | 21.8 (-41.6) | 20.6 (-34.7) | -0.66 | -0.92 | -0.53 | * | **** | ns | **** | **** |
| BG01345 (A) | 18.05 (14.9) | 17.33 (-1.9) | 18.79 (38.1) | 0.26 | -0.03 | 1.15 | ns | ns | * | ns | ** |
| BG01403 (A) | 22.54 (43.5) | 20.11 (13.8) | 24.74 (81.8) | 0.77 | 0.24 | 2.47 | ns | **** | *** | ns | **** |
| BG01540 (B) | 12.8 (-24.4) | 12.8 (-30.9) | 12.7 (-17.5) | -0.30 | -0.35 | -0.27 | ns | **** | ns | ** | * |
| BG01550 (F) | 23.8 (-30.2) | 28.7 (-23.1) | 19.1 (-39.4) | -0.52 | -0.51 | -0.61 | **** | **** | ns | ** | **** |
| BG01553 (F) | 24.2 (-33.3) | 24.9 (-40.3) | 23.4 (-23.3) | -0.65 | -1.81 | -0.37 | **** | **** | *** | **** | ** |
| BG01615 (A) | 17.4 (10.8) | 17.89 (1.2) | 16.93 (24.4) | 0.19 | 0.02 | 0.74 | * | ns | ns | ns | * |
| BG01677 (A) | 13.83 (-12.0) | 16.11 (-8.8) | 11.77 (-13.5) | -0.21 | -0.15 | -0.41 | **** | ns | ns | ns | ** |
| BG01700 (F) | 19.0 (-44.3) | 20.3 (-45.6) | 17.7 (-43.8) | -0.77 | -1.00 | -0.67 | * | **** | ns | **** | **** |
| BG01701 (F) | 21.7 (-36.6) | 23.1 (-38.1) | 20.1 (-36.1) | -0.63 | -0.84 | -0.55 | ** | **** | ns | **** | **** |
| BG01702 (A) | 16.2 (24.5) | 15.5 (8.9) | 16.8 (43.2) | 0.40 | 0.13 | 1.03 | ns | *** | * | ns | *** |
| BG01710 (A) | 14.6 (12.4) | 15.9 (11.4) | 13.2 (12.8) | 0.20 | 0.17 | 0.31 | ** | ns† | ns | ns | ns† |
| BG01878 (B) | 15.5 (-8.3) | 17.2 (-6.9) | 13.7 (-11.0) | -0.10 | -0.08 | -0.17 | ** | ns | ns | ns | ns |
| BG01918 (A) | 15.2 (16.9) | 14.4 (0.8) | 15.9 (35.8) | 0.27 | 0.01 | 0.85 | ns | * | * | ns | *** |
| BG01950 (B) | 21.7 (28.4) | 24.1 (30.1) | 19.2 (24.7) | 0.35 | 0.34 | 0.38 | ** | ** | ns | * | ** |
| BG01976 (B) | 25.8 (53.2) | 30.5 (64.7) | 21.0 (36.7) | 0.66 | 0.73 | 0.57 | **** | **** | * | **** | *** |
| BG02019 (B) | 18.5 (9.7) | 20.2 (8.9) | 16.9 (9.8) | 0.12 | 0.10 | 0.15 | ** | ns | ns | ns | ns |
| BG02039 (A) | 12.8 (-1.7) | 14.3 (0.5) | 11.2 (-4.6) | -0.03 | 0.01 | -0.11 | *** | ns | ns | ns | ns |
| BG02049 (B) | 12.4 (-26.6) | 12.5 (-32.7) | 12.3 (-20.1) | -0.33 | -0.37 | -0.31 | ns† | **** | ns | *** | ** |
| BG02128 (B) | 17.7 (4.9) | 21.5 (16.3) | 13.9 (-9.9) | 0.06 | 0.18 | -0.15 | *** | ns | ns | ns | ns |
| BG02395 (B) | 16.2 (-3.8) | 17.8 (-3.9) | 14.6 (-5.2) | -0.05 | -0.04 | -0.08 | * | ns | ns | ns | ns |
| BG02644 (B) | 17.2 (1.8) | 17.0 (-8.4) | 17.4 (13.2) | 0.02 | -0.10 | 0.20 | ns | ns | ns | ns | ns |

Letters in parenthesis after the Line name denote different co-isogenic *Canton S* host strains for *P{GT1}*-element insertion.  ^a^ Percent deviation from the mean life span of the control line. ^b^ standardized mutational effect (see text for explanation), ^c^ *S* and *L* denote the main cross-classified effects of Sex and Line, respectively in the ANOVA of life span. ns *P* > 0.1, † 0.05 < *P* < 0.1, * *P* < 0.05, ** *P* < 0.01,*** *P* < 0.001, **** *P* < 0.0001.

1. **C**limbing ability (week 1)

| **Line** | **Mutational effects** | | | | | | ***P*-values from ANOVA** | | | | |
| --- | --- | --- | --- | --- | --- | --- | --- | --- | --- | --- | --- |
|  | **Mean (%^a^)** | | | ***a/σ*_p_^b^** | | | **Sexes Pooled^c^** | | | **Sexes Separate** | |
|  | **♂,♀** | **♂** | **♀** | **♂,♀** | **♂** | **♀** | ***S*** | ***L*** | ***L*×*S*** | ***L*♂** | ***L*♀** |
| BG00004 (F) | 14.4 (-4.1) | 13.7 (-11.6) | 15.0 (3.9) | -0.03 | -0.09 | 0.03 | ns | ns | ns | ns | ns |
| BG00008 (F) | 17.2 (91.3) | 19.6 (154.1) | 14.7 (44.0) | 0.55 | 0.83 | 0.30 | ns | **** | ** | **** | * |
| BG00010 (F) | 15.6 (4.0) | 20.2 (30.3) | 10.9 (-24.2) | 0.03 | 0.24 | -0.17 | ** | ns | * | * | ns |
| BG00028 (F) | 6.6 ( -56.2) | 7.6 ( -51.3) | 5.6 ( -61.4) | -0.42 | -0.41 | -0.44 | ns | **** | ns | ** | *** |
| BG00037 (F) | 16.1 (79.2) | 14.3 (85.7) | 17.8 (74.3) | 0.48 | 0.46 | 0.50 | * | **** | ns | ** | *** |
| BG00041 (F) | 14.5 (62.1) | 15.8 (105.6) | 13.2 (29.3) | 0.38 | 0.57 | 0.20 | ns | **** | ns† | **** | ns |
| BG00043 (F) | 8.3 ( -44.9) | 10.0 (-35.8) | 6.5 ( -54.7) | -0.34 | -0.28 | -0.39 | ns | **** | ns | * | *** |
| BG00080 (B) | 17.4 (15.5) | 14.5 (7.4) | 20.3 (22.0) | 0.14 | 0.06 | 0.22 | ** | ns | ns | ns | ns† |
| BG00121 (F) | 16.4 (82.3) | 15.7 (103.5) | 17.0 (66.4) | 0.50 | 0.56 | 0.45 | ns | **** | ns | *** | ** |
| BG00297 (F) | 14.1 (-6.0) | 12.0 (-22.7) | 16.2 (12.0) | -0.05 | -0.18 | 0.09 | ns | ns | ns | ns | ns |
| BG00336 (B) | 17.9 (18.2) | 17.0 (25.6) | 18.7 (12.2) | 0.17 | 0.22 | 0.12 | ns | ns† | ns | ns† | ns |
| BG00346 (F) | 19.9 (32.8) | 19.0 (22.3) | 20.8 (44.1) | 0.25 | 0.18 | 0.31 | ns | ** | ns | ns | ** |
| BG00472 (F) | 15.2 (69.3) | 14.3 (85.3) | 16.1 (57.3) | 0.42 | 0.46 | 0.38 | ns | **** | ns | ** | ** |
| BG00495 (F) | 16.5 (9.8) | 18.9 (21.5) | 14.0 (-2.8) | 0.07 | 0.17 | -0.02 | ns | ns | ns | ns | ns |
| BG00528 (B) | 16.0 (6.0) | 14.4 (6.2) | 17.6 (5.8) | 0.05 | 0.05 | 0.06 | * | ns | ns | ns | ns |
| BG00757 (F) | 16.0 (78.8) | 16.4 (113.0) | 15.7 (53.1) | 0.48 | 0.61 | 0.36 | ns | **** | ns | *** | * |
| BG00761 (F) | 19.3 (28.7) | 18.7 (20.6) | 19.8 (37.4) | 0.22 | 0.16 | 0.27 | ns | * | ns | ns | * |
| BG00767 (B) | 16.1 (6.3) | 15.7 (16.0) | 16.4 (-1.6) | 0.06 | 0.14 | -0.02 | ns | ns | ns | ns | ns |
| BG00817 (F) | 13.6 (-9.1) | 13.3 (-14.4) | 13.9 (-3.5) | -0.07 | -0.11 | -0.02 | ns | ns | ns | ns | ns |
| BG00864 (B) | 18.4 (22.0) | 19.7 (45.8) | 17.1 (2.6) | 0.20 | 0.39 | 0.03 | ns | * | * | ** | ns |
| BG00907 (F) | 16.1 (79.9) | 16.1 (109.5) | 16.1 (57.7) | 0.48 | 0.59 | 0.39 | ns | **** | ns | *** | * |
| BG01031 (A) | 11.8 (-15.6) | 13.9 (10.3) | 9.7 ( -37.0) | -0.12 | 0.07 | -0.32 | ns | ns | * | ns | * |
| BG01042 (F) | 15.6 (4.2) | 16.0 (3.0) | 15.2 (5.5) | 0.03 | 0.02 | 0.04 | ns | ns | ns | ns | ns |
| BG01121 (F) | 8.0 ( -10.8) | 8.0 ( 4.3) | 8.0 ( -22.1) | -0.07 | 0.02 | -0.15 | ns | ns | ns | ns | ns |
| BG01540 (B) | 13.9 (-8.1) | 12.6 (-6.9) | 15.2 (-9.0) | -0.07 | -0.06 | -0.09 | ns† | ns | ns | ns | ns |
| BG01550 (F) | 16.1 (79.0) | 19.2 (149.4) | 12.9 (26.1) | 0.48 | 0.81 | 0.17 | ns | **** | ** | **** | ns |
| BG01700 (F) | 16.1 (79.2) | 18.2 (136.4) | 13.9 (36.2) | 0.48 | 0.74 | 0.24 | ns | **** | * | **** | ns |
| BG01701 (F) | 20.4 (127.5) | 21.4 (177.5) | 19.4 (89.9) | 0.77 | 0.96 | 0.60 | ns | **** | ns | **** | **** |
| BG01702 (A) | 11.9 (-15.3) | 11.2 (-11.1) | 12.5 (-18.7) | -0.12 | -0.08 | -0.16 | ns | ns | ns | ns | ns |
| BG01710 (A) | 10.3 (-26.3) | 8.6 ( -31.9) | 12.0 (-21.7) | -0.20 | -0.22 | -0.19 | ns† | * | ns | ns† | ns |
| BG01878 (B) | 13.5 (-10.7) | 10.0 (-25.9) | 16.9 (1.6) | -0.10 | -0.22 | 0.02 | *** | ns | ns | ns† | ns |
| BG01918 (A) | 7.4 ( -47.3) | 6.3 ( -49.9) | 8.4 ( -45.2) | -0.36 | -0.34 | -0.39 | ns | **** | ns | ** | ** |
| BG01950 (B) | 15.0 (-1.0) | 13.2 (-2.2) | 16.7 (0.0) | -0.01 | -0.02 | 0.00 | * | ns | ns | ns | ns |
| BG01976 (B) | 9.4 ( -37.6) | 7.5 ( -44.6) | 11.3 (-32.0) | -0.35 | -0.38 | -0.32 | * | **** | ns | ** | * |
| BG02019 (B) | 11.9 (-21.4) | 11.8 (-13.1) | 12.0 (-28.2) | -0.20 | -0.11 | -0.28 | ns | * | ns | ns | * |
| BG02039 (A) | 7.9 ( -43.7) | 6.7 ( -46.7) | 9.0 ( -41.3) | -0.34 | -0.32 | -0.35 | ns | **** | ns | ** | ** |
| BG02049 (B) | 7.7 ( -49.0) | 7.4 ( -45.6) | 8.0 ( -51.8) | -0.45 | -0.39 | -0.51 | ns | **** | ns | * | *** |
| BG02128 (B) | 14.0 (-7.2) | 15.3 (13.3) | 12.7 (-23.8) | -0.07 | 0.11 | -0.24 | ns | ns | ns† | ns | ns† |
| BG02395 (B) | 13.5 (-10.5) | 10.9 (-19.5) | 16.1 (-3.2) | -0.10 | -0.17 | -0.03 | ** | ns | ns | ns | ns |
| BG02644 (B) | 16.6 (9.8) | 17.6 (30.0) | 15.6 (-6.6) | 0.09 | 0.26 | -0.07 | ns | ns | ns† | ns† | ns |

Letters in parenthesis after the Line name denote different co-isogenic *Canton S* host strains for *P{GT1}*-element insertion.  ^a^ Percent deviation from the mean life span of the control line. ^b^ standardized mutational effect (see text for explanation), ^c^ *S* and *L* denote the main cross-classified effects of Sex and Line, respectively in the ANOVA of life span. ns *P* > 0.1, † 0.05 < *P* < 0.1, * *P* < 0.05, ** *P* < 0.01,*** *P* < 0.001, **** *P* < 0.0001.

1. **C**limbing ability (week 6)

| **Line** | **Mutational effects** | | | | | | ***P*-values from ANOVA** | | | | |
| --- | --- | --- | --- | --- | --- | --- | --- | --- | --- | --- | --- |
|  | **Mean (%^a^)** | | | ***a/σ*_p_^b^** | | | **Sexes Pooled^c^** | | | **Sexes Separate** | |
|  | **♂,♀** | **♂** | **♀** | **♂,♀** | **♂** | **♀** | ***S*** | ***L*** | ***L*×*S*** | ***L*♂** | ***L*♀** |
| BG00004 (F) | 8.1 ( 170.6) | 6.2 ( 210.0) | 10.0 (150.8) | 0.67 | 0.59 | 0.77 | ** | **** | ns | ** | **** |
| BG00008 (F) | 3.6 ( 8.0) | 1.1 ( -46.7) | 6.1 ( 31.4) | -0.07 | -0.13 | 0.16 | **** | ns | ns | ns | ns |
| BG00010 (F) | 5.2 ( 74.4) | 3.4 ( 71.7) | 7.0 ( 75.8) | 0.29 | 0.20 | 0.39 | *** | ** | ns | ns | * |
| BG00028 (F) | 3.8 ( 26.1) | 4.5 ( 123.3) | 3.1 ( -22.5) | 0.10 | 0.35 | -0.11 | ns | ns | ns† | ns† | ns |
| BG00037 (F) | 4.8 ( 42.5) | 3.9 ( 93.3) | 5.6 ( 20.7) | 0.07 | 0.26 | 0.11 | * | ns | ns | ns | ns |
| BG00041 (F) | 4.3 ( 29.5) | 4.3 ( 113.3) | 4.4 ( -6.4) | 0.02 | 0.32 | -0.03 | ns† | ns | ns | * | ns |
| BG00043 (F) | 2.1 ( -28.9) | 1.4 ( -30.0) | 2.9 ( -28.3) | -0.11 | -0.08 | -0.14 | * | ns | ns | ns | ns |
| BG00080 (B) | 10.6 (183.4) | 11.5 (817.3) | 9.7 ( 80.2) | 0.60 | 2.46 | 0.33 | ns | **** | * | **** | * |
| BG00121 (F) | 8.9 ( 167.5) | 7.5 ( 273.3) | 10.4 (122.1) | 0.56 | 0.77 | 0.63 | ** | **** | ns | *** | *** |
| BG00297 (F) | 12.1 (302.2) | 9.9 ( 393.3) | 14.3 (256.7) | 1.18 | 1.11 | 1.30 | ** | **** | ns | **** | **** |
| BG00336 (B) | 7.9 ( 111.7) | 9.2 ( 638.7) | 6.6 ( 22.2) | 0.37 | 1.92 | 0.09 | ns | *** | ** | **** | ns |
| BG00346 (F) | 8.1 ( 168.3) | 4.7 ( 136.7) | 11.4 (184.2) | 0.66 | 0.39 | 0.94 | *** | **** | * | ns† | **** |
| BG00472 (F) | 4.1 ( 23.0) | 2.2 ( 10.0) | 6.0 ( 28.6) | -0.01 | 0.03 | 0.15 | **** | ns | ns | ns | ns |
| BG00495 (F) | 10.5 (248.3) | 10.4 (420.0) | 10.5 (162.5) | 0.97 | 1.19 | 0.83 | ns | **** | ns | **** | *** |
| BG00528 (B) | 7.8 ( 107.2) | 3.0 ( 137.3) | 12.5 (132.1) | 0.35 | 0.41 | 0.54 | **** | **** | * | ns† | **** |
| BG00757 (F) | 5.2 ( 57.0) | 4.9 ( 146.7) | 5.5 ( 18.6) | 0.12 | 0.42 | 0.10 | ns† | * | ns | * | ns |
| BG00761 (F) | 7.9 ( 163.3) | 6.7 ( 233.3) | 9.1 ( 128.3) | 0.64 | 0.66 | 0.65 | * | **** | ns | ** | *** |
| BG00767 (B) | 6.1 ( 63.1) | 3.5 ( 180.0) | 8.7 ( 61.1) | 0.21 | 0.54 | 0.25 | **** | ** | ns | * | ns† |
| BG00817 (F) | 4.8 ( 60.0) | 2.2 ( 10.0) | 7.4 ( 85.0) | 0.23 | 0.03 | 0.43 | **** | * | ns† | ns | * |
| BG00864 (B) | 6.4 ( 72.0) | 5.8 ( 361.3) | 7.1 ( 31.5) | 0.24 | 1.09 | 0.13 | * | * | ns | ** | ns |
| BG00907 (F) | 3.9 ( 17.0) | 3.5 ( 75.0) | 4.3 ( -7.9) | -0.03 | 0.21 | -0.04 | * | ns | ns | ns | ns |
| BG01031 (A) | 2.6 ( -33.8) | 3.1 ( 12.2) | 2.2 ( -58.1) | -0.13 | 0.04 | -0.26 | ns | ns | ns† | ns | * |
| BG01042 (F) | 8.7 ( 190.6) | 10.6 (431.7) | 6.8 ( 70.0) | 0.74 | 1.22 | 0.36 | ns | **** | ** | **** | * |
| BG01121 (F) | 1.1 ( -67.5) | 1.2 ( -41.7) | 1.0 ( -78.6) | -0.37 | -0.12 | -0.41 | * | *** | * | ns | *** |
| BG01540 (B) | 5.9 ( 58.6) | 4.4 ( 252.0) | 7.5 ( 38.3) | 0.19 | 0.76 | 0.16 | ** | * | ns | * | ns |
| BG01550 (F) | 4.0 ( 20.5) | 3.5 ( 73.3) | 4.6 ( -2.1) | -0.02 | 0.21 | -0.01 | * | ns | ns | ns | ns |
| BG01700 (F) | 3.3 ( 0.0) | 3.2 ( 58.3) | 3.5 ( -25.0) | -0.10 | 0.17 | -0.13 | ns† | ns | ns | ns | ns |
| BG01701 (F) | 6.9 ( 107.5) | 5.4 ( 171.7) | 8.4 ( 80.0) | 0.32 | 0.49 | 0.41 | ** | *** | ns | * | * |
| BG01702 (A) | 8.1 ( 105.1) | 4.6 ( 69.5) | 11.6 (123.9) | 0.41 | 0.24 | 0.56 | **** | **** | * | ns | *** |
| BG01710 (A) | 3.9 ( -2.5) | 3.7 ( 34.1) | 4.0 ( -21.9) | -0.01 | 0.12 | -0.10 | ns | ns | ns | ns | ns |
| BG01878 (B) | 8.5 ( 126.4) | 9.4 ( 654.7) | 7.5 ( 38.9) | 0.42 | 1.97 | 0.16 | ns | *** | * | **** | ns |
| BG01918 (A) | 6.9 ( 73.4) | 6.4 ( 134.1) | 7.3 ( 41.3) | 0.29 | 0.47 | 0.19 | ns | ** | ns | * | ns |
| BG01950 (B) | 5.7 ( 52.9) | 8.5 ( 580.0) | 3.9 ( -28.4) | 0.17 | 1.75 | -0.12 | ns | * | *** | *** | ns |
| BG01976 (B) | 6.4 ( 70.6) | 7.1 ( 464.0) | 5.9 ( 9.9) | 0.23 | 1.40 | 0.04 | ns | ** | * | **** | ns |
| BG02019 (B) | 3.2 ( -14.4) | 2.1 ( 68.0) | 4.3 ( -20.4) | -0.05 | 0.20 | -0.08 | ** | ns | ns | ns | ns |
| BG02039 (A) | 7.2 ( 81.0) | 3.4 ( 24.4) | 10.9 (111.0) | 0.32 | 0.09 | 0.50 | **** | ** | * | ns | ** |
| BG02049 (B) | 8.5 ( 126.4) | 9.0 ( 622.7) | 7.9 ( 46.3) | 0.42 | 1.88 | 0.19 | ns | **** | * | **** | ns |
| BG02128 (B) | 6.3 ( 69.3) | 6.4 ( 409.3) | 6.3 ( 16.7) | 0.23 | 1.23 | 0.07 | ns† | * | ns† | ** | ns |
| BG02395 (B) | 8.2 ( 119.3) | 5.8 ( 366.7) | 10.6 (95.7) | 0.39 | 1.10 | 0.39 | *** | *** | ns | ** | ** |
| BG02644 (B) | 7.3 ( 94.7) | 6.4 ( 412.0) | 8.2 ( 51.2) | 0.31 | 1.24 | 0.21 | ** | *** | ns | *** | ns† |

Letters in parenthesis after the Line name denote different co-isogenic *Canton S* host strains for *P{GT1}*-element insertion.  ^a^ Percent deviation from the mean life span of the control line. ^b^ standardized mutational effect (see text for explanation), ^c^ *S* and *L* denote the main cross-classified effects of Sex and Line, respectively in the ANOVA of life span. ns *P* > 0.1, † 0.05 < *P* < 0.1, * *P* < 0.05, ** *P* < 0.01,*** *P* < 0.001, **** *P* < 0.0001.
