# Supplementary material for: Quantitative and Molecular Genetic Analyses of Mutations Increasing Drosophila Life Span
Source: PLoS Genet. 2010 Jul 29;6(7):e1001037. doi: 10.1371/journal.pgen.1001037 (PMC2912381; doi:10.1371/journal.pgen.1001037)
Supplement: Table S6 — Analyses of variance of pleiotropic effects of mutations with increased life span. (A) Starvation resistance; (B) Chill coma recovery; (C) Climbing ability. (0.10 MB DOC) [file pgen.1001037.s010.doc]

**Table S6**

**Analyses of variance of pleiotropic effects of mutations with increased life span**

1. **Starvation resistance**

| **Time** | **Analysis** | **Source** | **d.f.** | **SS** | ***F*** | ***P*** | **σ^2^** |
| --- | --- | --- | --- | --- | --- | --- | --- |
| **Week 1** | **Pooled Sexes** | Line | 49 | 357621 | 75.23 | <0.00001 | 89.297 |
|  |  | Sex | 1 | 28939.9 | 298.29 | <0.00001 | ― |
|  |  | Line × Sex | 49 | 95333.5 | 20.05 | <0.00001 | 61.380 |
|  |  | Rep (Line × Sex) | 200 | 21042 | 1.08 | 0.21 | 0.819 |
|  |  | Error | 2699 | 261853 | ― | ― | 97.019 |
|  | **Females** | Line | 49 | 344694 | 47.17 | <0.00001 | 230.254 |
|  |  | Rep (Sex) | 100 | 13536.9 | 0.91 | 0.73 | 0.000 |
|  |  | Error | 1349 | 201162 | ― | ― | 149.119 |
|  | **Males** | Line | 49 | 108275 | 49.15 | <0.00001 | 71.155 |
|  |  | Rep (Sex) | 100 | 7505.07 | 1.67 | 0.00007 | 3.009 |
|  |  | Error | 1350 | 60691.2 | ― | ― | 44.956 |
| **Week 6** | **Pooled Sexes** | Line | 49 | 130708 | 35.44 | <0.00001 | 32.251 |
|  |  | Sex | 1 | 26367.5 | 350.28 | <0.00001 | ― |
|  |  | Line × Sex | 49 | 39396.5 | 10.68 | <0.00001 | 25.506 |
|  |  | Rep (Line × Sex) | 195 | 13734.5 | 0.94 | 0.72 | 0.000 |
|  |  | Error | 2627 | 197749 | ― | ― | 75.276 |
|  | **Females** | Line | 49 | 135660 | 22.67 | <0.00001 | 89.697 |
|  |  | Rep (Sex) | 99 | 9959 | 0.82 | 0.89 | 0.000 |
|  |  | Error | 1339 | 163549 | ― | ― | 122.142 |
|  | **Males** | Line | 49 | 34917.1 | 26.84 | <0.00001 | 24.632 |
|  |  | Rep (Sex) | 96 | 3775.51 | 1.48 | 0.0024 | 1.306 |
|  |  | Error | 1288 | 34200 | ― | ― | 26.553 |

1. **Chill coma recovery**

| **Time** | **Analysis** | **Source** | **d.f.** | **SS** | ***F*** | ***P*** | **σ^2^** |
| --- | --- | --- | --- | --- | --- | --- | --- |
| **Week 1** | **Pooled Sexes** | Line | 49 | 17257 | 46.81 | <0.00001 | 5.152 |
|  |  | Sex | 1 | 460.106 | 61.16 | <0.00001 | ― |
|  |  | Line × Sex | 49 | 2341.26 | 6.35 | <0.00001 | 1.349 |
|  |  | Error | 2886 | 21712.4 | ― | ― | 7.523 |
|  | **Females** | Line | 49 | 12544.7 | 33.91 | <0.00001 | 8.316 |
|  |  | Error | 1444 | 10900.9 | ― | ― | 7.549 |
|  | **Males** | Line | 49 | 7215.4 | 19.64 | <0.00001 | 4.684 |
|  |  | Error | 1442 | 10811.4 | ― | ― | 7.498 |
| **Week 6** | **Pooled Sexes** | Line | 49 | 168114 | 66.96 | <0.00001 | 54.676 |
|  |  | Sex | 1 | 5243.63 | 102.34 | <0.00001 | ― |
|  |  | Line × Sex | 49 | 11939.8 | 4.76 | <0.00001 | 6.603 |
|  |  | Error | 2820 | 144493 | ― | ― | 51.238 |
|  | **Females** | Line | 49 | 79774.7 | 40.08 | <0.00001 | 54.070 |
|  |  | Error | 1418 | 57599.4 | ― | ― | 40.620 |
|  | **Males** | Line | 49 | 100592 | 33.12 | <0.00001 | 68.564 |
|  |  | Error | 1402 | 86893.5 | ― | ― | 61.978 |

1. **Climbing ability**

| **Time** | **Analysis** | **Source** | **d.f.** | **SS** | ***F*** | ***P*** | **σ^2^** |
| --- | --- | --- | --- | --- | --- | --- | --- |
| **Week 1** | **Pooled Sexes** | Line | 39 | 56660.1 | 20.49 | <0.00001 | 21.216 |
|  |  | Sex | 1 | 1728.9 | 24.38 | <0.00001 | ― |
|  |  | Line × Sex | 39 | 7015.11 | 2.54 | <0.00001 | 3.632 |
|  |  | Error | 2320 | 164527 | ― | ― | 70.917 |
|  | **Females** | Line | 39 | 26184.2 | 9.29 | <0.00001 | 19.970 |
|  |  | Error | 1160 | 83867.7 | ― | ― | 72.300 |
|  | **Males** | Line | 39 | 37491 | 13.82 | <0.00001 | 29.726 |
|  |  | Error | 1160 | 80659.3 | ― | ― | 69.534 |
| **Week 6** | **Pooled Sexes** | Line | 39 | 13554.3 | 9.06 | <0.00001 | 2.953 |
|  |  | Sex | 1 | 1560.81 | 40.69 | <0.00001 | ― |
|  |  | Line × Sex | 39 | 6734.11 | 4.50 | <0.00001 | 4.522 |
|  |  | Error | 2300 | 88229.1 | ― | ― | 38.360 |
|  | **Females** | Line | 39 | 11327.1 | 7.31 | <0.00001 | 8.356 |
|  |  | Error | 1160 | 46104.3 | ― | ― | 39.745 |
|  | **Males** | Line | 39 | 9009.81 | 6.25 | <0.00001 | 6.580 |
|  |  | Error | 1140 | 42124.9 | ― | ― | 36.952 |
