# Supplementary material for: Quantitative and Molecular Genetic Analyses of Mutations Increasing Drosophila Life Span
Source: PLoS Genet. 2010 Jul 29;6(7):e1001037. doi: 10.1371/journal.pgen.1001037 (PMC2912381; doi:10.1371/journal.pgen.1001037)
Supplement: Table S8 — Over-represented Biological Process Gene Ontology (GO) categories. (A) Females; (B) Males; (C) pyd; (D) mub; (E) crol; (F) CG10990; (G) CG9238; (H) BG00817; (I) esg. (1.12 MB DOC) [file pgen.1001037.s012.doc]

**Table S8**

**Over-represented Biological Process Gene Ontology (GO) categories**

1. **Females**

| **GO ID** | **Biological Process** | **Total probes on array** | **Probe sets in list** | **chi-square** | ***P*-value** |
| --- | --- | --- | --- | --- | --- |
| 15074 | DNA integration | 17 | 5 | 25.2441569 | 5.05E-07 |
| 9581 | detection of external stimulus | 67 | 10 | 17.8894362 | 2.34E-05 |
| 8015 | Circulation | 15 | 4 | 17.6774784 | 2.62E-05 |
| 8016 | regulation of heart contraction | 15 | 4 | 17.6774784 | 2.62E-05 |
| 51606 | detection of stimulus | 74 | 10 | 14.8735073 | 0.000115 |
| 51663 | oocyte nucleus localization during oocyte axis determination | 11 | 3 | 13.6742505 | 0.000217 |
| 6875 | metal ion homeostasis | 36 | 6 | 13.0071982 | 0.00031 |
| 51647 | nucleus localization | 27 | 5 | 12.8850061 | 0.000331 |
| 9583 | detection of light stimulus | 57 | 8 | 12.7845395 | 0.000349 |
| 5975 | carbohydrate metabolism | 592 | 43 | 12.5350834 | 0.000399 |
| 7602 | Phototransduction | 47 | 7 | 12.4746854 | 0.000413 |
| 6873 | cell ion homeostasis | 38 | 6 | 11.8569501 | 0.000574 |
| 30003 | cation homeostasis | 38 | 6 | 11.8569501 | 0.000574 |
| 9416 | response to light stimulus | 83 | 10 | 11.8172883 | 0.000587 |
| 9628 | response to abiotic stimulus | 179 | 17 | 11.5242507 | 0.000687 |
| 8643 | carbohydrate transport | 85 | 10 | 11.237046 | 0.000802 |
| 9582 | detection of abiotic stimulus | 62 | 8 | 10.8720391 | 0.000976 |
| 6508 | Proteolysis | 851 | 56 | 10.7554117 | 0.00104 |
| 6865 | amino acid transport | 52 | 7 | 10.3345726 | 0.001306 |
| 15837 | amine transport | 52 | 7 | 10.3345726 | 0.001306 |
| 51189 | prosthetic group metabolism | 65 | 8 | 9.88149926 | 0.001669 |
| 7440 | foregut morphogenesis | 14 | 3 | 9.68656 | 0.001856 |
| 50801 | ion homeostasis | 43 | 6 | 9.4841293 | 0.002073 |
| 9314 | response to radiation | 92 | 10 | 9.43404026 | 0.00213 |
| 7097 | nuclear migration | 23 | 4 | 9.30925735 | 0.00228 |
| 16059 | deactivation of rhodopsin mediated signaling | 15 | 3 | 8.72315151 | 0.003142 |
| 30005 | di-, tri-valent inorganic cation homeostasis | 34 | 5 | 8.70840719 | 0.003167 |
| 6874 | calcium ion homeostasis | 25 | 4 | 8.08810508 | 0.004456 |
| 48123 | oocyte dorsal/ventral axis determination (sensu Insecta) | 16 | 3 | 7.88549423 | 0.004983 |
| 40023 | establishment of nucleus localization | 26 | 4 | 7.55289556 | 0.005991 |
| 6858 | extracellular transport | 130 | 12 | 7.53962573 | 0.006036 |
| 6631 | fatty acid metabolism | 116 | 11 | 7.42707964 | 0.006425 |
| 15849 | organic acid transport | 62 | 7 | 7.19295253 | 0.007319 |
| 46942 | carboxylic acid transport | 62 | 7 | 7.19295253 | 0.007319 |
| 44255 | cellular lipid metabolism | 416 | 29 | 7.17702539 | 0.007384 |
| 6986 | response to unfolded protein | 28 | 4 | 6.60629308 | 0.010162 |
| 51789 | response to protein stimulus | 28 | 4 | 6.60629308 | 0.010162 |
| 8152 | Metabolism | 6114 | 301 | 6.30885488 | 0.012014 |
| 44262 | cellular carbohydrate metabolism | 375 | 26 | 6.29042243 | 0.012139 |
| 51239 | regulation of organismal physiological process | 41 | 5 | 6.05979112 | 0.013829 |
| 7026 | negative regulation of microtubule depolymerization | 10 | 2 | 5.81543434 | 0.015886 |
| 30722 | establishment of oocyte nucleus localization during oocyte axis determination | 10 | 2 | 5.81543434 | 0.015886 |
| 31111 | negative regulation of microtubule polymerization or depolymerization | 10 | 2 | 5.81543434 | 0.015886 |
| 48126 | establishment of oocyte nucleus localization during oocyte axis determination (sensu Insecta) | 10 | 2 | 5.81543434 | 0.015886 |
| 51662 | oocyte nucleus localization during oocyte axis determination (sensu Insecta) | 10 | 2 | 5.81543434 | 0.015886 |
| 6936 | muscle contraction | 113 | 10 | 5.5869872 | 0.018094 |
| 7051 | spindle organization and biogenesis | 43 | 5 | 5.4792657 | 0.019243 |
| 9408 | response to heat | 56 | 6 | 5.47580731 | 0.019281 |
| 7601 | visual perception | 133 | 11 | 5.02159253 | 0.025033 |
| 50953 | sensory perception of light stimulus | 133 | 11 | 5.02159253 | 0.025033 |
| 6665 | sphingolipid metabolism | 21 | 3 | 4.95471981 | 0.02602 |
| 6082 | organic acid metabolism | 499 | 31 | 4.46455318 | 0.034605 |
| 19752 | carboxylic acid metabolism | 499 | 31 | 4.46455318 | 0.034605 |
| 82 | G1/S transition of mitotic cell cycle | 12 | 2 | 4.33573275 | 0.03732 |
| 6541 | glutamine metabolism | 12 | 2 | 4.33573275 | 0.03732 |
| 6821 | chloride transport | 12 | 2 | 4.33573275 | 0.03732 |
| 7019 | microtubule depolymerization | 12 | 2 | 4.33573275 | 0.03732 |
| 9253 | peptidoglycan catabolism | 12 | 2 | 4.33573275 | 0.03732 |
| 31110 | regulation of microtubule polymerization or depolymerization | 12 | 2 | 4.33573275 | 0.03732 |
| 31114 | regulation of microtubule depolymerization | 12 | 2 | 4.33573275 | 0.03732 |
| 51640 | organelle localization | 48 | 5 | 4.27066537 | 0.038776 |
| 9605 | response to external stimulus | 157 | 12 | 4.21826417 | 0.039991 |
| 6030 | chitin metabolism | 108 | 9 | 4.20608993 | 0.040279 |
| 6629 | lipid metabolism | 582 | 35 | 4.20125797 | 0.040394 |
| 7498 | mesoderm development | 262 | 18 | 4.18986419 | 0.040666 |

1. **Males**

| **GO ID** | **Biological Process** | **Total probes on array** | **Probe sets in list** | **chi-square** | ***P*-value** |
| --- | --- | --- | --- | --- | --- |
| 45297 | post-mating behavior | 22 | 6 | 31.7926407 | 1.72E-08 |
| 15074 | DNA integration | 17 | 5 | 29.2505602 | 6.36E-08 |
| 5975 | carbohydrate metabolism | 592 | 48 | 28.7145431 | 8.39E-08 |
| 30239 | myofibril assembly | 15 | 4 | 20.5714286 | 5.74E-06 |
| 51146 | striated muscle cell differentiation | 15 | 4 | 20.5714286 | 5.74E-06 |
| 55001 | muscle cell development | 15 | 4 | 20.5714286 | 5.74E-06 |
| 55002 | striated muscle cell development | 15 | 4 | 20.5714286 | 5.74E-06 |
| 44262 | cellular carbohydrate metabolism | 375 | 31 | 19.5557143 | 9.77E-06 |
| 18208 | peptidyl-proline modification | 16 | 4 | 18.8595238 | 1.41E-05 |
| 18401 | peptidyl-proline hydroxylation to 4-hydroxy-L-proline | 16 | 4 | 18.8595238 | 1.41E-05 |
| 19471 | 4-hydroxyproline metabolism | 16 | 4 | 18.8595238 | 1.41E-05 |
| 19511 | peptidyl-proline hydroxylation | 16 | 4 | 18.8595238 | 1.41E-05 |
| 6508 | Proteolysis | 851 | 57 | 18.6379078 | 1.58E-05 |
| 51704 | interaction between organisms | 87 | 11 | 17.8229064 | 2.42E-05 |
| 7617 | mating behavior | 81 | 10 | 15.4931217 | 8.28E-05 |
| 51705 | behavioral interaction between organisms | 81 | 10 | 15.4931217 | 8.28E-05 |
| 9116 | nucleoside metabolism | 19 | 4 | 14.8290727 | 0.000118 |
| 19098 | reproductive behavior | 85 | 10 | 14.1204482 | 0.000171 |
| 5976 | polysaccharide metabolism | 214 | 18 | 11.8953716 | 0.000563 |
| 6030 | chitin metabolism | 108 | 11 | 11.5240079 | 0.000687 |
| 6807 | nitrogen compound metabolism | 506 | 34 | 11.2465462 | 0.000798 |
| 16052 | carbohydrate catabolism | 99 | 10 | 10.2865801 | 0.00134 |
| 44275 | cellular carbohydrate catabolism | 99 | 10 | 10.2865801 | 0.00134 |
| 44264 | cellular polysaccharide metabolism | 129 | 12 | 10.2166113 | 0.001392 |
| 6041 | glucosamine metabolism | 116 | 11 | 9.80051314 | 0.001745 |
| 6044 | N-acetylglucosamine metabolism | 116 | 11 | 9.80051314 | 0.001745 |
| 6040 | amino sugar metabolism | 117 | 11 | 9.60457875 | 0.001941 |
| 44248 | cellular catabolism | 394 | 27 | 9.57868625 | 0.001968 |
| 9308 | amine metabolism | 491 | 32 | 9.45017942 | 0.002111 |
| 6066 | alcohol metabolism | 238 | 18 | 8.80196078 | 0.003009 |
| 6633 | fatty acid biosynthesis | 39 | 5 | 8.31263736 | 0.003937 |
| 6090 | pyruvate metabolism | 18 | 3 | 7.81071429 | 0.005194 |
| 7062 | sister chromatid cohesion | 18 | 3 | 7.81071429 | 0.005194 |
| 9056 | Catabolism | 439 | 28 | 7.60308059 | 0.005827 |
| 272 | polysaccharide catabolism | 19 | 3 | 7.15802005 | 0.007463 |
| 6032 | chitin catabolism | 19 | 3 | 7.15802005 | 0.007463 |
| 6043 | glucosamine catabolism | 19 | 3 | 7.15802005 | 0.007463 |
| 6046 | N-acetylglucosamine catabolism | 19 | 3 | 7.15802005 | 0.007463 |
| 44247 | cellular polysaccharide catabolism | 19 | 3 | 7.15802005 | 0.007463 |
| 46348 | amino sugar catabolism | 19 | 3 | 7.15802005 | 0.007463 |
| 45861 | negative regulation of proteolysis | 10 | 2 | 6.88095238 | 0.008712 |
| 6510 | ATP-dependent proteolysis | 20 | 3 | 6.57440476 | 0.010346 |
| 5996 | monosaccharide metabolism | 136 | 11 | 6.53573179 | 0.010573 |
| 6629 | lipid metabolism | 582 | 34 | 6.31060383 | 0.012002 |
| 18193 | peptidyl-amino acid modification | 45 | 5 | 6.29761905 | 0.01209 |
| 8355 | olfactory learning | 59 | 6 | 6.2645682 | 0.012318 |
| 19722 | calcium-mediated signaling | 75 | 7 | 6.00714286 | 0.014248 |
| 6638 | neutral lipid metabolism | 11 | 2 | 5.96450216 | 0.014597 |
| 6639 | acylglycerol metabolism | 11 | 2 | 5.96450216 | 0.014597 |
| 46483 | heterocycle metabolism | 176 | 13 | 5.91072781 | 0.015049 |
| 16053 | organic acid biosynthesis | 47 | 5 | 5.75324215 | 0.016458 |
| 46394 | carboxylic acid biosynthesis | 47 | 5 | 5.75324215 | 0.016458 |
| 48627 | myoblast development | 34 | 4 | 5.64817927 | 0.017474 |
| 48628 | myoblast maturation | 34 | 4 | 5.64817927 | 0.017474 |
| 45445 | myoblast differentiation | 35 | 4 | 5.33333333 | 0.020921 |
| 9253 | peptidoglycan catabolism | 12 | 2 | 5.20714286 | 0.022494 |
| 16998 | cell wall catabolism | 12 | 2 | 5.20714286 | 0.022494 |
| 45924 | regulation of female receptivity | 12 | 2 | 5.20714286 | 0.022494 |
| 6575 | amino acid derivative metabolism | 64 | 6 | 5.20372024 | 0.022539 |
| 7612 | Learning | 64 | 6 | 5.20372024 | 0.022539 |
| 6006 | glucose metabolism | 81 | 7 | 4.96534392 | 0.02586 |
| 70 | mitotic sister chromatid segregation | 37 | 4 | 4.76087516 | 0.029114 |
| 819 | sister chromatid segregation | 37 | 4 | 4.76087516 | 0.029114 |
| 6100 | tricarboxylic acid cycle intermediate metabolism | 24 | 3 | 4.75803571 | 0.029162 |
| 9166 | nucleotide catabolism | 13 | 2 | 4.57216117 | 0.032496 |
| 46486 | glycerolipid metabolism | 13 | 2 | 4.57216117 | 0.032496 |
| 51186 | cofactor metabolism | 308 | 19 | 4.50037879 | 0.033887 |
| 6961 | antibacterial humoral response (sensu Protostomia) | 25 | 3 | 4.40238095 | 0.035889 |
| 21700 | developmental maturation | 39 | 4 | 4.25494505 | 0.039136 |
| 48469 | cell maturation | 39 | 4 | 4.25494505 | 0.039136 |
| 8610 | lipid biosynthesis | 121 | 9 | 4.18183786 | 0.040859 |
| 6092 | main pathways of carbohydrate metabolism | 122 | 9 | 4.07589774 | 0.043499 |
| 16082 | synaptic vesicle priming | 14 | 2 | 4.03333333 | 0.04461 |
| 50795 | regulation of behavior | 14 | 2 | 4.03333333 | 0.04461 |
| 8652 | amino acid biosynthesis | 106 | 8 | 3.88715184 | 0.048657 |
| 6732 | coenzyme metabolism | 278 | 17 | 3.87914526 | 0.048889 |
| 9408 | response to heat | 56 | 5 | 3.85208333 | 0.049684 |

1. ***pyd***

| **GO ID** | **Biological Process** | **Total probes on array** | **Probe sets in list** | **chi-square** | ***P*-value** |
| --- | --- | --- | --- | --- | --- |
| 51189 | prosthetic group metabolism | 65 | 7 | 21.669772 | 3.24E-06 |
| 15074 | DNA integration | 17 | 3 | 18.41944 | 1.77E-05 |
| 6090 | pyruvate metabolism | 18 | 3 | 17.105616 | 3.54E-05 |
| 9116 | nucleoside metabolism | 19 | 3 | 15.932407 | 6.56E-05 |
| 16042 | lipid catabolism | 34 | 4 | 14.122053 | 0.000171 |
| 6030 | chitin metabolism | 108 | 8 | 13.292609 | 0.000266 |
| 6721 | terpenoid metabolism | 11 | 2 | 12.758048 | 0.000354 |
| 16096 | polyisoprenoid metabolism | 11 | 2 | 12.758048 | 0.000354 |
| 42214 | terpene metabolism | 11 | 2 | 12.758048 | 0.000354 |
| 43449 | alkene metabolism | 11 | 2 | 12.758048 | 0.000354 |
| 6041 | glucosamine metabolism | 116 | 8 | 11.61256 | 0.000655 |
| 6044 | N-acetylglucosamine metabolism | 116 | 8 | 11.61256 | 0.000655 |
| 6040 | amino sugar metabolism | 117 | 8 | 11.420402 | 0.000726 |
| 6508 | Proteolysis | 851 | 33 | 10.857685 | 0.000984 |
| 44242 | cellular lipid catabolism | 13 | 2 | 10.261182 | 0.001359 |
| 44264 | cellular polysaccharide metabolism | 129 | 8 | 9.373507 | 0.002201 |
| 18991 | Oviposition | 14 | 2 | 9.2849879 | 0.00231 |
| 6858 | extracellular transport | 130 | 8 | 9.2221916 | 0.002391 |
| 6820 | anion transport | 112 | 7 | 8.3365894 | 0.003885 |
| 6732 | coenzyme metabolism | 278 | 13 | 7.7314303 | 0.005427 |
| 7632 | visual behavior | 16 | 2 | 7.7069297 | 0.005501 |
| 6398 | histone mRNA 3'-end processing | 18 | 2 | 6.4893366 | 0.010852 |
| 8334 | histone mRNA metabolism | 18 | 2 | 6.4893366 | 0.010852 |
| 7617 | mating behavior | 81 | 5 | 5.801498 | 0.016013 |
| 51705 | behavioral interaction between organisms | 81 | 5 | 5.801498 | 0.016013 |
| 51186 | cofactor metabolism | 308 | 13 | 5.7026094 | 0.01694 |
| 6807 | nitrogen compound metabolism | 506 | 19 | 5.5442009 | 0.018542 |
| 9126 | purine nucleoside monophosphate metabolism | 20 | 2 | 5.5240691 | 0.018757 |
| 9127 | purine nucleoside monophosphate biosynthesis | 20 | 2 | 5.5240691 | 0.018757 |
| 9167 | purine ribonucleoside monophosphate metabolism | 20 | 2 | 5.5240691 | 0.018757 |
| 9168 | purine ribonucleoside monophosphate biosynthesis | 20 | 2 | 5.5240691 | 0.018757 |
| 6790 | sulfur metabolism | 85 | 5 | 5.2298929 | 0.022202 |
| 19098 | reproductive behavior | 85 | 5 | 5.2298929 | 0.022202 |
| 2778 | antibacterial peptide production | 21 | 2 | 5.1135284 | 0.02374 |
| 2780 | antibacterial peptide biosynthesis | 21 | 2 | 5.1135284 | 0.02374 |
| 2808 | regulation of antibacterial peptide biosynthesis | 21 | 2 | 5.1135284 | 0.02374 |
| 6665 | sphingolipid metabolism | 21 | 2 | 5.1135284 | 0.02374 |
| 6963 | positive regulation of antibacterial peptide biosynthesis | 21 | 2 | 5.1135284 | 0.02374 |
| 51704 | interaction between organisms | 87 | 5 | 4.9668378 | 0.025838 |
| 6626 | protein targeting to mitochondrion | 41 | 3 | 4.8726514 | 0.027286 |
| 6720 | isoprenoid metabolism | 22 | 2 | 4.7423111 | 0.02943 |
| 45297 | post-mating behavior | 22 | 2 | 4.7423111 | 0.02943 |
| 6839 | mitochondrial transport | 66 | 4 | 4.4637198 | 0.034622 |
| 7097 | nuclear migration | 23 | 2 | 4.4052882 | 0.035828 |
| 9156 | ribonucleoside monophosphate biosynthesis | 23 | 2 | 4.4052882 | 0.035828 |
| 9161 | ribonucleoside monophosphate metabolism | 23 | 2 | 4.4052882 | 0.035828 |
| 44272 | sulfur compound biosynthesis | 44 | 3 | 4.2589351 | 0.039044 |
| 44260 | cellular macromolecule metabolism | 2570 | 72 | 4.1995455 | 0.040435 |
| 9123 | nucleoside monophosphate metabolism | 24 | 2 | 4.0981853 | 0.042929 |
| 9124 | nucleoside monophosphate biosynthesis | 24 | 2 | 4.0981853 | 0.042929 |
| 6960 | antimicrobial humoral response (sensu Protostomia) | 45 | 3 | 4.0745043 | 0.043535 |
| 19731 | antibacterial humoral response | 45 | 3 | 4.0745043 | 0.043535 |
| 5976 | polysaccharide metabolism | 214 | 9 | 3.9028796 | 0.048203 |

1. ***mub***

| **GO ID** | **Biological Process** | **Total probes on array** | **Probe sets in list** | **chi-square** | ***P*-value** |
| --- | --- | --- | --- | --- | --- |
| 6139 | nucleobase, nucleoside, nucleotide and nucleic acid metabolism | 2234 | 954 | 53.435721 | 2.7E-13 |
| 44238 | primary metabolism | 5502 | 2165 | 51.657296 | 6.6E-13 |
| 6259 | DNA metabolism | 526 | 272 | 50.472928 | 1.2E-12 |
| 44237 | cellular metabolism | 5627 | 2199 | 47.884424 | 4.5E-12 |
| 43170 | macromolecule metabolism | 4600 | 1807 | 42.170457 | 8.4E-11 |
| 8152 | Metabolism | 6114 | 2356 | 41.981997 | 9.2E-11 |
| 6260 | DNA replication | 143 | 90 | 36.192284 | 1.8E-09 |
| 6396 | RNA processing | 374 | 190 | 32.352705 | 1.3E-08 |
| 6281 | DNA repair | 128 | 80 | 31.433828 | 2.1E-08 |
| 43283 | biopolymer metabolism | 2978 | 1176 | 29.371221 | 6E-08 |
| 6974 | response to DNA damage stimulus | 140 | 84 | 28.666678 | 8.6E-08 |
| 16070 | RNA metabolism | 1579 | 655 | 28.192074 | 1.1E-07 |
| 50875 | cellular physiological process | 7572 | 2812 | 26.176108 | 3.1E-07 |
| 6261 | DNA-dependent DNA replication | 74 | 50 | 25.136428 | 5.3E-07 |
| 9719 | response to endogenous stimulus | 167 | 92 | 22.609866 | 2E-06 |
| 16071 | mRNA metabolism | 286 | 141 | 20.581749 | 5.7E-06 |
| 6397 | mRNA processing | 274 | 135 | 19.640725 | 9.3E-06 |
| 51276 | chromosome organization and biogenesis | 294 | 142 | 18.52485 | 1.7E-05 |
| 7067 | Mitosis | 288 | 138 | 17.206563 | 3.4E-05 |
| 6270 | DNA replication initiation | 16 | 15 | 17.096268 | 3.6E-05 |
| 279 | M phase | 383 | 176 | 16.989064 | 3.8E-05 |
| 87 | M phase of mitotic cell cycle | 289 | 138 | 16.86529 | 4E-05 |
| 16072 | rRNA metabolism | 59 | 38 | 16.472708 | 4.9E-05 |
| 7049 | cell cycle | 605 | 261 | 15.913104 | 6.6E-05 |
| 16568 | chromatin modification | 132 | 71 | 15.759779 | 7.2E-05 |
| 7001 | chromosome organization and biogenesis (sensu Eukaryota) | 273 | 130 | 15.633577 | 7.7E-05 |
| 6323 | DNA packaging | 237 | 115 | 15.395544 | 8.7E-05 |
| 6325 | establishment and/or maintenance of chromatin architecture | 237 | 115 | 15.395544 | 8.7E-05 |
| 42254 | ribosome biogenesis and assembly | 59 | 37 | 14.703156 | 0.00013 |
| 7046 | ribosome biogenesis | 51 | 33 | 14.518072 | 0.00014 |
| 19538 | protein metabolism | 2606 | 991 | 14.32292 | 0.00015 |
| 74 | regulation of progression through cell cycle | 236 | 113 | 14.029758 | 0.00018 |
| 51726 | regulation of cell cycle | 237 | 113 | 13.690001 | 0.00022 |
| 44267 | cellular protein metabolism | 2451 | 932 | 13.456425 | 0.00024 |
| 8380 | RNA splicing | 211 | 102 | 13.37161 | 0.00026 |
| 278 | mitotic cell cycle | 341 | 154 | 13.231124 | 0.00028 |
| 44249 | cellular biosynthesis | 1093 | 438 | 13.072116 | 0.0003 |
| 31497 | chromatin assembly | 64 | 38 | 12.488797 | 0.00041 |
| 9058 | Biosynthesis | 1180 | 467 | 11.990498 | 0.00053 |
| 6277 | DNA amplification | 17 | 14 | 11.921921 | 0.00055 |
| 9987 | cellular process | 8166 | 2935 | 11.926006 | 0.00055 |
| 6352 | transcription initiation | 96 | 52 | 11.89848 | 0.00056 |
| 9892 | negative regulation of metabolism | 218 | 103 | 11.825682 | 0.00058 |
| 9059 | macromolecule biosynthesis | 618 | 258 | 11.802372 | 0.00059 |
| 7059 | chromosome segregation | 162 | 80 | 11.781273 | 0.0006 |
| 6367 | transcription initiation from RNA polymerase II promoter | 94 | 51 | 11.751856 | 0.00061 |
| 6342 | chromatin silencing | 32 | 22 | 11.642582 | 0.00064 |
| 31507 | heterochromatin formation | 32 | 22 | 11.642582 | 0.00064 |
| 45814 | negative regulation of gene expression, epigenetic | 32 | 22 | 11.642582 | 0.00064 |
| 6412 | protein biosynthesis | 577 | 242 | 11.557173 | 0.00067 |
| 7582 | physiological process | 8256 | 2962 | 11.363889 | 0.00075 |
| 6333 | chromatin assembly or disassembly | 171 | 83 | 11.130411 | 0.00085 |
| 375 | RNA splicing, via transesterification reactions | 198 | 94 | 11.104383 | 0.00086 |
| 377 | RNA splicing, via transesterification reactions with bulged adenosine as nucleophile | 198 | 94 | 11.104383 | 0.00086 |
| 398 | nuclear mRNA splicing, via spliceosome | 198 | 94 | 11.104383 | 0.00086 |
| 7307 | chorion gene amplification | 16 | 13 | 10.717435 | 0.00106 |
| 30261 | chromosome condensation | 29 | 20 | 10.681327 | 0.00108 |
| 45934 | negative regulation of nucleobase, nucleoside, nucleotide and nucleic acid metabolism | 158 | 77 | 10.55838 | 0.00116 |
| 44260 | cellular macromolecule metabolism | 2570 | 962 | 10.486429 | 0.0012 |
| 51261 | protein depolymerization | 20 | 15 | 10.105033 | 0.00148 |
| 16569 | covalent chromatin modification | 42 | 26 | 9.8919559 | 0.00166 |
| 16570 | histone modification | 42 | 26 | 9.8919559 | 0.00166 |
| 6364 | rRNA processing | 40 | 25 | 9.8230712 | 0.00172 |
| 31123 | RNA 3'-end processing | 34 | 22 | 9.6787149 | 0.00186 |
| 31124 | mRNA 3'-end processing | 34 | 22 | 9.6787149 | 0.00186 |
| 9566 | Fertilization | 15 | 12 | 9.5260304 | 0.00203 |
| 6220 | pyrimidine nucleotide metabolism | 17 | 13 | 9.2121982 | 0.0024 |
| 6996 | organelle organization and biogenesis | 1019 | 399 | 8.9211978 | 0.00282 |
| 70 | mitotic sister chromatid segregation | 37 | 23 | 8.8742523 | 0.00289 |
| 819 | sister chromatid segregation | 37 | 23 | 8.8742523 | 0.00289 |
| 9299 | mRNA transcription | 25 | 17 | 8.7104252 | 0.00316 |
| 6338 | chromatin remodeling | 59 | 33 | 8.630161 | 0.00331 |
| 15931 | nucleobase, nucleoside, nucleotide and nucleic acid transport | 48 | 28 | 8.6213477 | 0.00332 |
| 7338 | fertilization (sensu Metazoa) | 14 | 11 | 8.3505123 | 0.00386 |
| 31324 | negative regulation of cellular metabolism | 203 | 92 | 8.0969546 | 0.00443 |
| 6221 | pyrimidine nucleotide biosynthesis | 16 | 12 | 8.0840265 | 0.00447 |
| 6379 | mRNA cleavage | 16 | 12 | 8.0840265 | 0.00447 |
| 45132 | meiotic chromosome segregation | 49 | 28 | 7.9701121 | 0.00476 |
| 16042 | lipid catabolism | 34 | 21 | 7.92838 | 0.00487 |
| 6398 | histone mRNA 3'-end processing | 18 | 13 | 7.9116799 | 0.00491 |
| 8334 | histone mRNA metabolism | 18 | 13 | 7.9116799 | 0.00491 |
| 6913 | nucleocytoplasmic transport | 81 | 42 | 7.8946182 | 0.00496 |
| 51168 | nuclear export | 30 | 19 | 7.8001628 | 0.00522 |
| 6366 | transcription from RNA polymerase II promoter | 845 | 332 | 7.7669738 | 0.00532 |
| 6378 | mRNA polyadenylation | 24 | 16 | 7.7241266 | 0.00545 |
| 43631 | RNA polyadenylation | 24 | 16 | 7.7241266 | 0.00545 |
| 6399 | tRNA metabolism | 110 | 54 | 7.7042835 | 0.00551 |
| 16458 | gene silencing | 54 | 30 | 7.6331568 | 0.00573 |
| 16481 | negative regulation of transcription | 142 | 67 | 7.6295516 | 0.00574 |
| 45892 | negative regulation of transcription, DNA-dependent | 126 | 60 | 7.2154972 | 0.00723 |
| 31109 | microtubule polymerization or depolymerization | 13 | 10 | 7.1945461 | 0.00731 |
| 44242 | cellular lipid catabolism | 13 | 10 | 7.1945461 | 0.00731 |
| 6206 | pyrimidine base metabolism | 35 | 21 | 7.1666694 | 0.00743 |
| 50791 | regulation of physiological process | 1734 | 648 | 6.842 | 0.0089 |
| 9116 | nucleoside metabolism | 19 | 13 | 6.7835555 | 0.0092 |
| 245 | spliceosome assembly | 23 | 15 | 6.765333 | 0.00929 |
| 6631 | fatty acid metabolism | 116 | 55 | 6.4480563 | 0.01111 |
| 7143 | female meiosis | 52 | 28 | 6.2445116 | 0.01246 |
| 7051 | spindle organization and biogenesis | 43 | 24 | 6.2229801 | 0.01261 |
| 6310 | DNA recombination | 50 | 27 | 6.0964957 | 0.01355 |
| 6275 | regulation of DNA replication | 12 | 9 | 6.0630199 | 0.0138 |
| 6383 | transcription from RNA polymerase III promoter | 12 | 9 | 6.0630199 | 0.0138 |
| 7019 | microtubule depolymerization | 12 | 9 | 6.0630199 | 0.0138 |
| 8062 | eclosion rhythm | 12 | 9 | 6.0630199 | 0.0138 |
| 31110 | regulation of microtubule polymerization or depolymerization | 12 | 9 | 6.0630199 | 0.0138 |
| 31114 | regulation of microtubule depolymerization | 12 | 9 | 6.0630199 | 0.0138 |
| 51322 | Anaphase | 12 | 9 | 6.0630199 | 0.0138 |
| 51244 | regulation of cellular physiological process | 1683 | 626 | 6.0197119 | 0.01415 |
| 6479 | protein amino acid methylation | 26 | 16 | 5.965443 | 0.01459 |
| 8213 | protein amino acid alkylation | 26 | 16 | 5.965443 | 0.01459 |
| 51129 | negative regulation of cell organization and biogenesis | 14 | 10 | 5.9024406 | 0.01512 |
| 6405 | RNA export from nucleus | 24 | 15 | 5.8938427 | 0.01519 |
| 6950 | response to stress | 312 | 130 | 5.8389423 | 0.01568 |
| 6720 | isoprenoid metabolism | 22 | 14 | 5.8377951 | 0.01569 |
| 1775 | cell activation | 18 | 12 | 5.793095 | 0.01609 |
| 9451 | RNA modification | 18 | 12 | 5.793095 | 0.01609 |
| 8033 | tRNA processing | 33 | 19 | 5.56789 | 0.01829 |
| 51327 | M phase of meiotic cell cycle | 134 | 61 | 5.532771 | 0.01866 |
| 9314 | response to radiation | 92 | 44 | 5.4266129 | 0.01983 |
| 51169 | nuclear transport | 75 | 37 | 5.4199486 | 0.01991 |
| 43118 | negative regulation of physiological process | 323 | 133 | 5.3218291 | 0.02106 |
| 51243 | negative regulation of cellular physiological process | 315 | 130 | 5.3208566 | 0.02107 |
| 51321 | meiotic cell cycle | 135 | 61 | 5.2600354 | 0.02182 |
| 7126 | Meiosis | 130 | 59 | 5.2430036 | 0.02204 |
| 51128 | regulation of cell organization and biogenesis | 66 | 33 | 5.185624 | 0.02277 |
| 9416 | response to light stimulus | 83 | 40 | 5.1536302 | 0.0232 |
| 43037 | Translation | 226 | 96 | 5.1373686 | 0.02342 |
| 44255 | cellular lipid metabolism | 416 | 167 | 5.0872254 | 0.0241 |
| 226 | microtubule cytoskeleton organization and biogenesis | 113 | 52 | 5.0656086 | 0.02441 |
| 6350 | Transcription | 1212 | 454 | 5.0171732 | 0.0251 |
| 6721 | terpenoid metabolism | 11 | 8 | 4.9625991 | 0.0259 |
| 16096 | polyisoprenoid metabolism | 11 | 8 | 4.9625991 | 0.0259 |
| 42214 | terpene metabolism | 11 | 8 | 4.9625991 | 0.0259 |
| 43449 | alkene metabolism | 11 | 8 | 4.9625991 | 0.0259 |
| 6665 | sphingolipid metabolism | 21 | 13 | 4.9459779 | 0.02615 |
| 32774 | RNA biosynthesis | 1140 | 428 | 4.9365796 | 0.02629 |
| 6082 | organic acid metabolism | 499 | 197 | 4.9033739 | 0.0268 |
| 19752 | carboxylic acid metabolism | 499 | 197 | 4.9033739 | 0.0268 |
| 6406 | mRNA export from nucleus | 19 | 12 | 4.8817382 | 0.02714 |
| 42168 | heme metabolism | 13 | 9 | 4.8605276 | 0.02748 |
| 7076 | mitotic chromosome condensation | 15 | 10 | 4.8275791 | 0.02801 |
| 46011 | regulation of oskar mRNA translation | 15 | 10 | 4.8275791 | 0.02801 |
| 6351 | transcription, DNA-dependent | 1137 | 426 | 4.7275702 | 0.02968 |
| 6633 | fatty acid biosynthesis | 39 | 21 | 4.6833837 | 0.03046 |
| 122 | negative regulation of transcription from RNA polymerase II promoter | 70 | 34 | 4.5768857 | 0.03241 |
| 9056 | Catabolism | 439 | 174 | 4.5516533 | 0.03289 |
| 7028 | cytoplasm organization and biogenesis | 95 | 44 | 4.4676999 | 0.03454 |
| 9586 | rhodopsin mediated phototransduction | 24 | 14 | 4.3106739 | 0.03787 |
| 51236 | establishment of RNA localization | 33 | 18 | 4.2430687 | 0.03941 |
| 6414 | translational elongation | 20 | 12 | 4.0952397 | 0.043 |
| 7304 | eggshell formation (sensu Insecta) | 74 | 35 | 4.0437131 | 0.04434 |
| 30703 | eggshell formation | 74 | 35 | 4.0437131 | 0.04434 |
| 6997 | nuclear organization and biogenesis | 45 | 23 | 4.0348176 | 0.04457 |
| 9583 | detection of light stimulus | 57 | 28 | 4.0088089 | 0.04526 |
| 43543 | protein amino acid acylation | 29 | 16 | 3.9566761 | 0.04669 |
| 16571 | histone methylation | 16 | 10 | 3.9292285 | 0.04745 |
| 6013 | mannose metabolism | 10 | 7 | 3.9026153 | 0.04821 |
| 7026 | negative regulation of microtubule depolymerization | 10 | 7 | 3.9026153 | 0.04821 |
| 7094 | mitotic spindle checkpoint | 10 | 7 | 3.9026153 | 0.04821 |
| 31111 | negative regulation of microtubule polymerization or depolymerization | 10 | 7 | 3.9026153 | 0.04821 |
| 31577 | spindle checkpoint | 10 | 7 | 3.9026153 | 0.04821 |
| 6123 | mitochondrial electron transport, cytochrome c to oxygen | 14 | 9 | 3.8779948 | 0.04892 |
| 16043 | cell organization and biogenesis | 1833 | 667 | 3.8632156 | 0.04936 |
| 50794 | regulation of cellular process | 1833 | 667 | 3.8632156 | 0.04936 |
| 212 | meiotic spindle organization and biogenesis | 12 | 8 | 3.8620633 | 0.04939 |
| 6563 | L-serine metabolism | 12 | 8 | 3.8620633 | 0.04939 |
| 6783 | heme biosynthesis | 12 | 8 | 3.8620633 | 0.04939 |

1. ***crol***

| **GO ID** | **Biological Process** | **Total probes on array** | **Probe sets in list** | **chi-square** | ***P*-value** |
| --- | --- | --- | --- | --- | --- |
| 42254 | ribosome biogenesis and assembly | 59 | 23 | 25.347598 | 4.79E-07 |
| 7046 | ribosome biogenesis | 51 | 20 | 22.324662 | 2.3E-06 |
| 16072 | rRNA metabolism | 59 | 21 | 18.876896 | 1.39E-05 |
| 6364 | rRNA processing | 40 | 16 | 18.626729 | 1.59E-05 |
| 7279 | pole cell formation | 14 | 7 | 12.564718 | 0.000393 |
| 6398 | histone mRNA 3'-end processing | 18 | 8 | 11.524515 | 0.000687 |
| 8334 | histone mRNA metabolism | 18 | 8 | 11.524515 | 0.000687 |
| 6383 | transcription from RNA polymerase III promoter | 12 | 6 | 10.769758 | 0.001032 |
| 16042 | lipid catabolism | 34 | 12 | 10.575528 | 0.001146 |
| 6732 | coenzyme metabolism | 278 | 60 | 10.50856 | 0.001188 |
| 7028 | cytoplasm organization and biogenesis | 95 | 25 | 9.7175759 | 0.001825 |
| 19538 | protein metabolism | 2606 | 431 | 9.6273834 | 0.001917 |
| 44267 | cellular protein metabolism | 2451 | 407 | 9.5890532 | 0.001957 |
| 44242 | cellular lipid catabolism | 13 | 6 | 9.2921761 | 0.002301 |
| 44260 | cellular macromolecule metabolism | 2570 | 423 | 8.8468236 | 0.002936 |
| 15074 | DNA integration | 17 | 7 | 8.6560984 | 0.00326 |
| 51186 | cofactor metabolism | 308 | 63 | 8.3377754 | 0.003883 |
| 51169 | nuclear transport | 75 | 20 | 8.1242539 | 0.004368 |
| 6508 | Proteolysis | 851 | 151 | 7.3144589 | 0.00684 |
| 30431 | Sleep | 15 | 6 | 6.9850233 | 0.008219 |
| 7277 | pole cell development | 19 | 7 | 6.8110471 | 0.00906 |
| 18193 | peptidyl-amino acid modification | 45 | 13 | 6.7746242 | 0.009246 |
| 6913 | nucleocytoplasmic transport | 81 | 20 | 6.2055545 | 0.012735 |
| 18208 | peptidyl-proline modification | 16 | 6 | 6.0744514 | 0.013715 |
| 18401 | peptidyl-proline hydroxylation to 4-hydroxy-L-proline | 16 | 6 | 6.0744514 | 0.013715 |
| 19471 | 4-hydroxyproline metabolism | 16 | 6 | 6.0744514 | 0.013715 |
| 19511 | peptidyl-proline hydroxylation | 16 | 6 | 6.0744514 | 0.013715 |
| 51704 | interaction between organisms | 87 | 21 | 5.9777278 | 0.014488 |
| 19236 | response to pheromone | 13 | 5 | 5.3520666 | 0.020698 |
| 6606 | protein import into nucleus | 49 | 13 | 5.1922114 | 0.022688 |
| 19098 | reproductive behavior | 85 | 20 | 5.143942 | 0.023328 |
| 6479 | protein amino acid methylation | 26 | 8 | 4.983954 | 0.025583 |
| 8213 | protein amino acid alkylation | 26 | 8 | 4.983954 | 0.025583 |
| 9310 | amine catabolism | 65 | 16 | 4.9075982 | 0.026739 |
| 44270 | nitrogen compound catabolism | 65 | 16 | 4.9075982 | 0.026739 |
| 51189 | prosthetic group metabolism | 65 | 16 | 4.9075982 | 0.026739 |
| 6858 | extracellular transport | 130 | 28 | 4.8549576 | 0.027567 |
| 51170 | nuclear import | 50 | 13 | 4.8504132 | 0.02764 |
| 7617 | mating behavior | 81 | 19 | 4.8254922 | 0.028042 |
| 51705 | behavioral interaction between organisms | 81 | 19 | 4.8254922 | 0.028042 |
| 45297 | post-mating behavior | 22 | 7 | 4.7695881 | 0.028967 |
| 7320 | Insemination | 10 | 4 | 4.6566822 | 0.030933 |
| 48609 | reproductive organismal physiological process | 10 | 4 | 4.6566822 | 0.030933 |
| 50876 | reproductive physiological process | 10 | 4 | 4.6566822 | 0.030933 |
| 6418 | tRNA aminoacylation for protein translation | 66 | 16 | 4.6311289 | 0.031397 |
| 43039 | tRNA aminoacylation | 66 | 16 | 4.6311289 | 0.031397 |
| 18991 | Oviposition | 14 | 5 | 4.5302106 | 0.033302 |
| 6399 | tRNA metabolism | 110 | 24 | 4.4291772 | 0.03533 |
| 43038 | amino acid activation | 67 | 16 | 4.3671646 | 0.036638 |
| 43037 | Translation | 226 | 44 | 4.3301765 | 0.037442 |
| 9063 | amino acid catabolism | 62 | 15 | 4.3080221 | 0.037933 |
| 7602 | Phototransduction | 47 | 12 | 4.2035149 | 0.04034 |
| 9059 | macromolecule biosynthesis | 618 | 107 | 4.0867102 | 0.043222 |
| 15931 | nucleobase, nucleoside, nucleotide and nucleic acid transport | 48 | 12 | 3.897869 | 0.048347 |

1. ***CG10990***

| **GO ID** | **Biological Process** | **Total probes on array** | **Probe sets in list** | **chi-square** | ***P*-value** |
| --- | --- | --- | --- | --- | --- |
| 18208 | peptidyl-proline modification | 16 | 4 | 45.6605 | 1.41E-11 |
| 18401 | peptidyl-proline hydroxylation to 4-hydroxy-L-proline | 16 | 4 | 45.6605 | 1.41E-11 |
| 19471 | 4-hydroxyproline metabolism | 16 | 4 | 45.6605 | 1.41E-11 |
| 19511 | peptidyl-proline hydroxylation | 16 | 4 | 45.6605 | 1.41E-11 |
| 15074 | DNA integration | 17 | 4 | 42.54038 | 6.92E-11 |
| 6575 | amino acid derivative metabolism | 64 | 6 | 19.21475 | 1.17E-05 |
| 6875 | metal ion homeostasis | 36 | 4 | 16.3905 | 5.15E-05 |
| 45297 | post-mating behavior | 22 | 3 | 16.24165 | 5.58E-05 |
| 15012 | heparan sulfate proteoglycan biosynthesis | 11 | 2 | 15.61002 | 7.78E-05 |
| 30201 | heparan sulfate proteoglycan metabolism | 11 | 2 | 15.61002 | 7.78E-05 |
| 40018 | positive regulation of body size | 11 | 2 | 15.61002 | 7.78E-05 |
| 6873 | cell ion homeostasis | 38 | 4 | 15.17978 | 9.77E-05 |
| 30003 | cation homeostasis | 38 | 4 | 15.17978 | 9.77E-05 |
| 7629 | flight behavior | 24 | 3 | 14.46002 | 0.000143 |
| 6023 | aminoglycan biosynthesis | 12 | 2 | 14.01177 | 0.000182 |
| 6024 | glycosaminoglycan biosynthesis | 12 | 2 | 14.01177 | 0.000182 |
| 30307 | positive regulation of cell growth | 13 | 2 | 12.66229 | 0.000373 |
| 44242 | cellular lipid catabolism | 13 | 2 | 12.66229 | 0.000373 |
| 50801 | ion homeostasis | 43 | 4 | 12.66096 | 0.000373 |
| 44272 | sulfur compound biosynthesis | 44 | 4 | 12.22845 | 0.000471 |
| 18193 | peptidyl-amino acid modification | 45 | 4 | 11.816 | 0.000587 |
| 51704 | interaction between organisms | 87 | 6 | 11.71069 | 0.000621 |
| 6022 | aminoglycan metabolism | 14 | 2 | 11.50827 | 0.000693 |
| 18991 | Oviposition | 14 | 2 | 11.50827 | 0.000693 |
| 30203 | glycosaminoglycan metabolism | 14 | 2 | 11.50827 | 0.000693 |
| 7549 | dosage compensation | 16 | 2 | 9.64001 | 0.001904 |
| 45793 | positive regulation of cell size | 16 | 2 | 9.64001 | 0.001904 |
| 7584 | response to nutrient | 17 | 2 | 8.874035 | 0.002893 |
| 8286 | insulin receptor signaling pathway | 17 | 2 | 8.874035 | 0.002893 |
| 30166 | proteoglycan biosynthesis | 17 | 2 | 8.874035 | 0.002893 |
| 30005 | di-, tri-valent inorganic cation homeostasis | 34 | 3 | 8.762053 | 0.003076 |
| 6090 | pyruvate metabolism | 18 | 2 | 8.195251 | 0.0042 |
| 6398 | histone mRNA 3'-end processing | 18 | 2 | 8.195251 | 0.0042 |
| 7016 | cytoskeletal anchoring | 18 | 2 | 8.195251 | 0.0042 |
| 8334 | histone mRNA metabolism | 18 | 2 | 8.195251 | 0.0042 |
| 7617 | mating behavior | 81 | 5 | 7.987311 | 0.004711 |
| 51705 | behavioral interaction between organisms | 81 | 5 | 7.987311 | 0.004711 |
| 6508 | Proteolysis | 851 | 27 | 7.658912 | 0.005649 |
| 6029 | proteoglycan metabolism | 19 | 2 | 7.58989 | 0.00587 |
| 7160 | cell-matrix adhesion | 38 | 3 | 7.350185 | 0.006706 |
| 31589 | cell-substrate adhesion | 38 | 3 | 7.350185 | 0.006706 |
| 19098 | reproductive behavior | 85 | 5 | 7.287244 | 0.006945 |
| 6936 | muscle contraction | 113 | 6 | 7.11752 | 0.007633 |
| 21700 | developmental maturation | 39 | 3 | 7.044873 | 0.007949 |
| 48469 | cell maturation | 39 | 3 | 7.044873 | 0.007949 |
| 6807 | nitrogen compound metabolism | 506 | 17 | 5.95938 | 0.014639 |
| 8344 | adult locomotory behavior | 44 | 3 | 5.739257 | 0.01659 |
| 1558 | regulation of cell growth | 23 | 2 | 5.711143 | 0.016858 |
| 7475 | apposition of dorsal and ventral wing surfaces | 24 | 2 | 5.343212 | 0.020803 |
| 45927 | positive regulation of growth | 24 | 2 | 5.343212 | 0.020803 |
| 9308 | amine metabolism | 491 | 16 | 5.022978 | 0.025013 |
| 6874 | calcium ion homeostasis | 25 | 2 | 5.006215 | 0.025256 |
| 9991 | response to extracellular stimulus | 25 | 2 | 5.006215 | 0.025256 |
| 31667 | response to nutrient levels | 25 | 2 | 5.006215 | 0.025256 |
| 7365 | periodic partitioning | 48 | 3 | 4.904662 | 0.026784 |
| 19722 | calcium-mediated signaling | 75 | 4 | 4.789137 | 0.02864 |
| 6812 | cation transport | 461 | 15 | 4.683028 | 0.030462 |
| 1539 | ciliary or flagellar motility | 27 | 2 | 4.411273 | 0.035702 |
| 46483 | heterocycle metabolism | 176 | 7 | 4.154402 | 0.041526 |
| 1501 | skeletal development | 28 | 2 | 4.147682 | 0.041692 |
| 9725 | response to hormone stimulus | 28 | 2 | 4.147682 | 0.041692 |
| 35265 | organ growth | 28 | 2 | 4.147682 | 0.041692 |
| 46620 | regulation of organ size | 28 | 2 | 4.147682 | 0.041692 |
| 6519 | amino acid and derivative metabolism | 362 | 12 | 4.010363 | 0.045221 |

1. ***CG9238***

| **GO ID** | **Biological Process** | **Total probes on array** | **Probe sets in list** | **chi-square** | ***P*-value** |
| --- | --- | --- | --- | --- | --- |
| 8586 | wing vein morphogenesis | 26 | 6 | 13.83947 | 0.000199 |
| 578 | embryonic axis specification | 72 | 11 | 11.66086 | 0.000638 |
| 35222 | wing disc pattern formation | 37 | 7 | 11.40387 | 0.000733 |
| 7632 | visual behavior | 16 | 4 | 10.50396 | 0.001191 |
| 48512 | circadian behavior | 39 | 7 | 10.32262 | 0.001314 |
| 7623 | circadian rhythm | 58 | 9 | 9.868228 | 0.001682 |
| 48511 | rhythmic process | 59 | 9 | 9.508593 | 0.002045 |
| 51239 | regulation of organismal physiological process | 41 | 7 | 9.357947 | 0.00222 |
| 45475 | locomotor rhythm | 33 | 6 | 9.069679 | 0.002599 |
| 8362 | embryonic cuticle biosynthesis (sensu Insecta) | 11 | 3 | 9.020769 | 0.002669 |
| 35296 | regulation of tube diameter | 11 | 3 | 9.020769 | 0.002669 |
| 7622 | rhythmic behavior | 42 | 7 | 8.914124 | 0.00283 |
| 7367 | segment polarity determination | 34 | 6 | 8.561982 | 0.003433 |
| 7351 | regional subdivision | 62 | 9 | 8.510296 | 0.003531 |
| 8595 | determination of anterior/posterior axis, embryo | 62 | 9 | 8.510296 | 0.003531 |
| 9063 | amino acid catabolism | 62 | 9 | 8.510296 | 0.003531 |
| 50801 | ion homeostasis | 43 | 7 | 8.493587 | 0.003564 |
| 2168 | larval development (sensu Insecta) | 53 | 8 | 8.258592 | 0.004056 |
| 6807 | nitrogen compound metabolism | 506 | 44 | 8.077281 | 0.004482 |
| 42306 | regulation of protein import into nucleus | 19 | 4 | 7.896378 | 0.004953 |
| 48100 | wing disc anterior/posterior pattern formation | 19 | 4 | 7.896378 | 0.004953 |
| 6541 | glutamine metabolism | 12 | 3 | 7.877974 | 0.005004 |
| 7458 | progression of morphogenetic furrow (sensu Endopterygota) | 12 | 3 | 7.877974 | 0.005004 |
| 8062 | eclosion rhythm | 12 | 3 | 7.877974 | 0.005004 |
| 7610 | Behavior | 319 | 30 | 7.77049 | 0.005311 |
| 6875 | metal ion homeostasis | 36 | 6 | 7.640678 | 0.005707 |
| 9310 | amine catabolism | 65 | 9 | 7.619888 | 0.005773 |
| 44270 | nitrogen compound catabolism | 65 | 9 | 7.619888 | 0.005773 |
| 51704 | interaction between organisms | 87 | 11 | 7.415344 | 0.006467 |
| 6112 | energy reserve metabolism | 20 | 4 | 7.212388 | 0.00724 |
| 51223 | regulation of protein transport | 20 | 4 | 7.212388 | 0.00724 |
| 16335 | morphogenesis of larval imaginal disc epithelium | 13 | 3 | 6.919736 | 0.008525 |
| 6873 | cell ion homeostasis | 38 | 6 | 6.828318 | 0.008972 |
| 30003 | cation homeostasis | 38 | 6 | 6.828318 | 0.008972 |
| 7474 | wing vein specification | 21 | 4 | 6.598954 | 0.010204 |
| 46822 | regulation of nucleocytoplasmic transport | 21 | 4 | 6.598954 | 0.010204 |
| 6606 | protein import into nucleus | 49 | 7 | 6.379541 | 0.011544 |
| 7617 | mating behavior | 81 | 10 | 6.325408 | 0.011902 |
| 51705 | behavioral interaction between organisms | 81 | 10 | 6.325408 | 0.011902 |
| 9308 | amine metabolism | 491 | 41 | 6.143596 | 0.013189 |
| 5977 | glycogen metabolism | 14 | 3 | 6.10651 | 0.013468 |
| 6073 | glucan metabolism | 14 | 3 | 6.10651 | 0.013468 |
| 6376 | mRNA splice site selection | 14 | 3 | 6.10651 | 0.013468 |
| 7440 | foregut morphogenesis | 14 | 3 | 6.10651 | 0.013468 |
| 9453 | energy taxis | 14 | 3 | 6.10651 | 0.013468 |
| 16082 | synaptic vesicle priming | 14 | 3 | 6.10651 | 0.013468 |
| 18991 | Oviposition | 14 | 3 | 6.10651 | 0.013468 |
| 30237 | female sex determination | 14 | 3 | 6.10651 | 0.013468 |
| 35161 | imaginal disc lineage restriction | 14 | 3 | 6.10651 | 0.013468 |
| 42331 | Phototaxis | 14 | 3 | 6.10651 | 0.013468 |
| 48190 | wing disc dorsal/ventral pattern formation | 14 | 3 | 6.10651 | 0.013468 |
| 51170 | nuclear import | 50 | 7 | 6.084485 | 0.013637 |
| 9967 | positive regulation of signal transduction | 22 | 4 | 6.046453 | 0.013934 |
| 45297 | post-mating behavior | 22 | 4 | 6.046453 | 0.013934 |
| 9952 | anterior/posterior pattern formation | 179 | 18 | 6.020778 | 0.014138 |
| 51248 | negative regulation of protein metabolism | 61 | 8 | 5.926876 | 0.014912 |
| 6520 | amino acid metabolism | 328 | 29 | 5.755059 | 0.016441 |
| 2376 | immune system process | 261 | 24 | 5.663552 | 0.017321 |
| 35282 | Segmentation | 157 | 16 | 5.61247 | 0.017833 |
| 7448 | anterior/posterior pattern formation, imaginal disc | 23 | 4 | 5.546938 | 0.018513 |
| 19098 | reproductive behavior | 85 | 10 | 5.530552 | 0.018687 |
| 8015 | Circulation | 15 | 3 | 5.409291 | 0.02003 |
| 8016 | regulation of heart contraction | 15 | 3 | 5.409291 | 0.02003 |
| 9070 | serine family amino acid biosynthesis | 15 | 3 | 5.409291 | 0.02003 |
| 30713 | stalk formation (sensu Insecta) | 15 | 3 | 5.409291 | 0.02003 |
| 19748 | secondary metabolism | 86 | 10 | 5.346696 | 0.020762 |
| 7450 | dorsal/ventral pattern formation, imaginal disc | 24 | 4 | 5.093785 | 0.024012 |
| 6508 | Proteolysis | 851 | 64 | 5.052744 | 0.024587 |
| 7350 | blastoderm segmentation | 137 | 14 | 4.958311 | 0.025966 |
| 42127 | regulation of cell proliferation | 44 | 6 | 4.896345 | 0.026914 |
| 16042 | lipid catabolism | 34 | 5 | 4.869614 | 0.027334 |
| 30005 | di-, tri-valent inorganic cation homeostasis | 34 | 5 | 4.869614 | 0.027334 |
| 8589 | regulation of smoothened signaling pathway | 16 | 3 | 4.80633 | 0.028355 |
| 6874 | calcium ion homeostasis | 25 | 4 | 4.681431 | 0.03049 |
| 50803 | regulation of synapse structure and function | 25 | 4 | 4.681431 | 0.03049 |
| 7447 | imaginal disc pattern formation | 67 | 8 | 4.614765 | 0.031698 |
| 6206 | pyrimidine base metabolism | 35 | 5 | 4.556815 | 0.032788 |
| 16333 | morphogenesis of follicular epithelium | 35 | 5 | 4.556815 | 0.032788 |
| 5975 | carbohydrate metabolism | 592 | 46 | 4.535485 | 0.033199 |
| 7611 | learning and/or memory | 91 | 10 | 4.506762 | 0.033761 |
| 9636 | response to toxin | 142 | 14 | 4.356157 | 0.036875 |
| 6519 | amino acid and derivative metabolism | 362 | 30 | 4.317968 | 0.037712 |
| 271 | polysaccharide biosynthesis | 17 | 3 | 4.280991 | 0.038541 |
| 15074 | DNA integration | 17 | 3 | 4.280991 | 0.038541 |
| 43284 | biopolymer biosynthesis | 17 | 3 | 4.280991 | 0.038541 |
| 9880 | embryonic pattern specification | 169 | 16 | 4.25739 | 0.03908 |
| 8355 | olfactory learning | 59 | 7 | 3.965763 | 0.046434 |
| 1539 | ciliary or flagellar motility | 27 | 4 | 3.960989 | 0.046566 |
| 16334 | establishment and/or maintenance of polarity of follicular epithelium | 27 | 4 | 3.960989 | 0.046566 |
| 9948 | anterior/posterior axis specification | 159 | 15 | 3.934878 | 0.047295 |
| 2217 | physiological defense response | 120 | 12 | 3.93379 | 0.047325 |
| 7365 | periodic partitioning | 48 | 6 | 3.924057 | 0.0476 |
| 5976 | polysaccharide metabolism | 214 | 19 | 3.843428 | 0.049941 |

1. **BG00817**

| **GO ID** | **Biological Process** | **Total probes on array** | **Probe sets in list** | **chi-square** | ***P*-value** |
| --- | --- | --- | --- | --- | --- |
| 21700 | developmental maturation | 39 | 15 | 22.23801 | 2.41E-06 |
| 48469 | cell maturation | 39 | 15 | 22.23801 | 2.41E-06 |
| 16042 | lipid catabolism | 34 | 12 | 15.000982 | 0.000107 |
| 48627 | myoblast development | 34 | 12 | 15.000982 | 0.000107 |
| 48628 | myoblast maturation | 34 | 12 | 15.000982 | 0.000107 |
| 30239 | myofibril assembly | 15 | 7 | 14.718103 | 0.000125 |
| 51146 | striated muscle cell differentiation | 15 | 7 | 14.718103 | 0.000125 |
| 55001 | muscle cell development | 15 | 7 | 14.718103 | 0.000125 |
| 55002 | striated muscle cell development | 15 | 7 | 14.718103 | 0.000125 |
| 45445 | myoblast differentiation | 35 | 12 | 14.126107 | 0.000171 |
| 51189 | prosthetic group metabolism | 65 | 18 | 12.935642 | 0.000322 |
| 42692 | muscle cell differentiation | 44 | 13 | 10.968332 | 0.000927 |
| 7519 | striated muscle development | 80 | 20 | 10.884125 | 0.00097 |
| 7520 | myoblast fusion | 22 | 8 | 10.624161 | 0.001116 |
| 6508 | Proteolysis | 851 | 136 | 10.309196 | 0.001324 |
| 16202 | regulation of striated muscle development | 13 | 5 | 7.41267 | 0.006477 |
| 44242 | cellular lipid catabolism | 13 | 5 | 7.41267 | 0.006477 |
| 44255 | cellular lipid metabolism | 416 | 69 | 6.7556343 | 0.009345 |
| 48637 | skeletal muscle development | 65 | 15 | 6.3952996 | 0.011442 |
| 48741 | skeletal muscle fiber development | 65 | 15 | 6.3952996 | 0.011442 |
| 48747 | muscle fiber development | 65 | 15 | 6.3952996 | 0.011442 |
| 44260 | cellular macromolecule metabolism | 2570 | 356 | 6.1637732 | 0.013039 |
| 44267 | cellular protein metabolism | 2451 | 340 | 6.0151963 | 0.014183 |
| 6732 | coenzyme metabolism | 278 | 48 | 6.0020948 | 0.014289 |
| 6629 | lipid metabolism | 582 | 91 | 5.8376952 | 0.015686 |
| 19538 | protein metabolism | 2606 | 359 | 5.7042073 | 0.016924 |
| 43543 | protein amino acid acylation | 29 | 8 | 5.6927822 | 0.017035 |
| 51186 | cofactor metabolism | 308 | 52 | 5.6921104 | 0.017041 |
| 5975 | carbohydrate metabolism | 592 | 92 | 5.6184996 | 0.017772 |
| 7076 | mitotic chromosome condensation | 15 | 5 | 5.5444096 | 0.01854 |
| 8152 | Metabolism | 6114 | 806 | 5.4180174 | 0.01993 |
| 51247 | positive regulation of protein metabolism | 11 | 4 | 5.3120805 | 0.021178 |
| 6473 | protein amino acid acetylation | 25 | 7 | 5.1741284 | 0.022926 |
| 6643 | membrane lipid metabolism | 119 | 23 | 5.0543262 | 0.024565 |
| 6575 | amino acid derivative metabolism | 64 | 14 | 4.988339 | 0.025519 |
| 7632 | visual behavior | 16 | 5 | 4.8082014 | 0.028325 |
| 18208 | peptidyl-proline modification | 16 | 5 | 4.8082014 | 0.028325 |
| 18401 | peptidyl-proline hydroxylation to 4-hydroxy-L-proline | 16 | 5 | 4.8082014 | 0.028325 |
| 19471 | 4-hydroxyproline metabolism | 16 | 5 | 4.8082014 | 0.028325 |
| 19511 | peptidyl-proline hydroxylation | 16 | 5 | 4.8082014 | 0.028325 |
| 7413 | axonal fasciculation | 21 | 6 | 4.6652088 | 0.03078 |
| 6644 | phospholipid metabolism | 103 | 20 | 4.484763 | 0.034198 |
| 8062 | eclosion rhythm | 12 | 4 | 4.4355276 | 0.035198 |
| 9253 | peptidoglycan catabolism | 12 | 4 | 4.4355276 | 0.035198 |
| 16998 | cell wall catabolism | 12 | 4 | 4.4355276 | 0.035198 |
| 51235 | maintenance of localization | 32 | 8 | 4.3536499 | 0.03693 |
| 15074 | DNA integration | 17 | 5 | 4.1728947 | 0.041076 |
| 45475 | locomotor rhythm | 33 | 8 | 3.9761005 | 0.04615 |
| 6936 | muscle contraction | 113 | 21 | 3.8570575 | 0.049537 |
| 6457 | protein folding | 173 | 30 | 3.8451116 | 0.049891 |

1. ***esg***

| **GO ID** | **Biological Process** | **Total probes on array** | **Probe sets in list** | **chi-square** | ***P*-value** |
| --- | --- | --- | --- | --- | --- |
| 9416 | response to light stimulus | 83 | 33 | 30.697958 | 3.01E-08 |
| 9314 | response to radiation | 92 | 34 | 26.567439 | 2.54E-07 |
| 9583 | detection of light stimulus | 57 | 24 | 25.389942 | 4.68E-07 |
| 7602 | Phototransduction | 47 | 21 | 25.215235 | 5.13E-07 |
| 7601 | visual perception | 133 | 43 | 23.523477 | 1.23E-06 |
| 50953 | sensory perception of light stimulus | 133 | 43 | 23.523477 | 1.23E-06 |
| 9581 | detection of external stimulus | 67 | 26 | 22.85758 | 1.74E-06 |
| 6396 | RNA processing | 374 | 94 | 21.326243 | 3.87E-06 |
| 9582 | detection of abiotic stimulus | 62 | 24 | 20.976024 | 4.65E-06 |
| 51606 | detection of stimulus | 74 | 26 | 17.867623 | 2.37E-05 |
| 16072 | rRNA metabolism | 59 | 22 | 17.573013 | 2.76E-05 |
| 43283 | biopolymer metabolism | 2978 | 555 | 16.622039 | 4.56E-05 |
| 6403 | RNA localization | 127 | 38 | 16.43208 | 5.04E-05 |
| 7046 | ribosome biogenesis | 51 | 19 | 15.143545 | 9.96E-05 |
| 42254 | ribosome biogenesis and assembly | 59 | 21 | 14.924506 | 0.000112 |
| 9586 | rhodopsin mediated phototransduction | 24 | 11 | 13.919505 | 0.000191 |
| 6139 | nucleobase, nucleoside, nucleotide and nucleic acid metabolism | 2234 | 417 | 12.71841 | 0.000362 |
| 43170 | macromolecule metabolism | 4600 | 816 | 12.458879 | 0.000416 |
| 9605 | response to external stimulus | 157 | 42 | 12.278321 | 0.000458 |
| 6364 | rRNA processing | 40 | 15 | 12.148663 | 0.000491 |
| 44242 | cellular lipid catabolism | 13 | 7 | 12.079046 | 0.00051 |
| 6259 | DNA metabolism | 526 | 114 | 12.05584 | 0.000516 |
| 6270 | DNA replication initiation | 16 | 8 | 12.021288 | 0.000526 |
| 16071 | mRNA metabolism | 286 | 68 | 11.96176 | 0.000543 |
| 44238 | primary metabolism | 5502 | 962 | 11.447349 | 0.000716 |
| 16070 | RNA metabolism | 1579 | 299 | 10.687413 | 0.001079 |
| 7338 | fertilization (sensu Metazoa) | 14 | 7 | 10.518627 | 0.001182 |
| 6397 | mRNA processing | 274 | 64 | 10.305805 | 0.001326 |
| 15931 | nucleobase, nucleoside, nucleotide and nucleic acid transport | 48 | 16 | 9.5426352 | 0.002008 |
| 9451 | RNA modification | 18 | 8 | 9.5001081 | 0.002055 |
| 6399 | tRNA metabolism | 110 | 30 | 9.4314312 | 0.002133 |
| 9566 | Fertilization | 15 | 7 | 9.1871682 | 0.002437 |
| 16059 | deactivation of rhodopsin mediated signaling | 15 | 7 | 9.1871682 | 0.002437 |
| 8152 | Metabolism | 6114 | 1052 | 9.1043944 | 0.00255 |
| 8062 | eclosion rhythm | 12 | 6 | 9.0159658 | 0.002676 |
| 51236 | establishment of RNA localization | 33 | 12 | 9.0059628 | 0.002691 |
| 6406 | mRNA export from nucleus | 19 | 8 | 8.4633142 | 0.003624 |
| 6281 | DNA repair | 128 | 33 | 8.3328028 | 0.003894 |
| 7549 | dosage compensation | 16 | 7 | 8.04174 | 0.004571 |
| 7315 | pole plasm assembly | 64 | 19 | 8.011149 | 0.004649 |
| 9628 | response to abiotic stimulus | 179 | 43 | 7.9485622 | 0.004813 |
| 6505 | GPI anchor metabolism | 13 | 6 | 7.7008616 | 0.005519 |
| 6506 | GPI anchor biosynthesis | 13 | 6 | 7.7008616 | 0.005519 |
| 44237 | cellular metabolism | 5627 | 964 | 7.5796736 | 0.005903 |
| 9620 | response to fungus | 20 | 8 | 7.5458781 | 0.006015 |
| 6405 | RNA export from nucleus | 24 | 9 | 7.2891981 | 0.006937 |
| 51028 | mRNA transport | 24 | 9 | 7.2891981 | 0.006937 |
| 19094 | pole plasm mRNA localization | 57 | 17 | 7.2752256 | 0.006991 |
| 9893 | positive regulation of metabolism | 118 | 30 | 7.1477308 | 0.007506 |
| 31325 | positive regulation of cellular metabolism | 118 | 30 | 7.1477308 | 0.007506 |
| 50657 | nucleic acid transport | 32 | 11 | 7.1346109 | 0.007561 |
| 50658 | RNA transport | 32 | 11 | 7.1346109 | 0.007561 |
| 48113 | pole plasm assembly (sensu Insecta) | 62 | 18 | 7.051791 | 0.007919 |
| 46474 | glycerophospholipid biosynthesis | 17 | 7 | 7.0495134 | 0.007929 |
| 7316 | pole plasm RNA localization | 58 | 17 | 6.8744495 | 0.008744 |
| 7028 | cytoplasm organization and biogenesis | 95 | 25 | 6.8562594 | 0.008833 |
| 6913 | nucleocytoplasmic transport | 81 | 22 | 6.8111583 | 0.009059 |
| 8298 | intracellular mRNA localization | 72 | 20 | 6.7228359 | 0.009519 |
| 9453 | energy taxis | 14 | 6 | 6.5960271 | 0.010221 |
| 30237 | female sex determination | 14 | 6 | 6.5960271 | 0.010221 |
| 42331 | Phototaxis | 14 | 6 | 6.5960271 | 0.010221 |
| 45475 | locomotor rhythm | 33 | 11 | 6.5605617 | 0.010426 |
| 278 | mitotic cell cycle | 341 | 72 | 6.4268553 | 0.011241 |
| 50875 | cellular physiological process | 7572 | 1274 | 6.3505113 | 0.011735 |
| 45087 | innate immune response | 55 | 16 | 6.3106921 | 0.012001 |
| 7613 | Memory | 38 | 12 | 6.1277697 | 0.013307 |
| 16042 | lipid catabolism | 34 | 11 | 6.0295027 | 0.014069 |
| 31123 | RNA 3'-end processing | 34 | 11 | 6.0295027 | 0.014069 |
| 31124 | mRNA 3'-end processing | 34 | 11 | 6.0295027 | 0.014069 |
| 51168 | nuclear export | 30 | 10 | 5.964147 | 0.0146 |
| 51169 | nuclear transport | 75 | 20 | 5.7758149 | 0.016248 |
| 48512 | circadian behavior | 39 | 12 | 5.6648115 | 0.017309 |
| 48149 | behavioral response to ethanol | 15 | 6 | 5.6594086 | 0.017362 |
| 6418 | tRNA aminoacylation for protein translation | 66 | 18 | 5.6588587 | 0.017367 |
| 43039 | tRNA aminoacylation | 66 | 18 | 5.6588587 | 0.017367 |
| 45893 | positive regulation of transcription, DNA-dependent | 85 | 22 | 5.6448081 | 0.017507 |
| 6974 | response to DNA damage stimulus | 140 | 33 | 5.5629821 | 0.018344 |
| 7067 | Mitosis | 288 | 61 | 5.5609427 | 0.018366 |
| 87 | M phase of mitotic cell cycle | 289 | 61 | 5.4325817 | 0.019764 |
| 50832 | defense response to fungus | 19 | 7 | 5.4279056 | 0.019817 |
| 43038 | amino acid activation | 67 | 18 | 5.3483135 | 0.020742 |
| 48112 | oocyte anterior/posterior axis determination (sensu Insecta) | 67 | 18 | 5.3483135 | 0.020742 |
| 7623 | circadian rhythm | 58 | 16 | 5.2454826 | 0.022004 |
| 6519 | amino acid and derivative metabolism | 362 | 74 | 5.2392156 | 0.022083 |
| 6023 | aminoglycan biosynthesis | 12 | 5 | 5.1692969 | 0.02299 |
| 6024 | glycosaminoglycan biosynthesis | 12 | 5 | 5.1692969 | 0.02299 |
| 6275 | regulation of DNA replication | 12 | 5 | 5.1692969 | 0.02299 |
| 9253 | peptidoglycan catabolism | 12 | 5 | 5.1692969 | 0.02299 |
| 45934 | negative regulation of nucleobase, nucleoside, nucleotide and nucleic acid metabolism | 158 | 36 | 5.0891983 | 0.024075 |
| 8654 | phospholipid biosynthesis | 36 | 11 | 5.0820254 | 0.024175 |
| 8380 | RNA splicing | 211 | 46 | 5.0447277 | 0.024701 |
| 48511 | rhythmic process | 59 | 16 | 4.9251147 | 0.026469 |
| 7632 | visual behavior | 16 | 6 | 4.8594654 | 0.027495 |
| 9584 | detection of visible light | 16 | 6 | 4.8594654 | 0.027495 |
| 19732 | antifungal humoral response | 16 | 6 | 4.8594654 | 0.027495 |
| 46489 | phosphoinositide biosynthesis | 16 | 6 | 4.8594654 | 0.027495 |
| 9892 | negative regulation of metabolism | 218 | 47 | 4.8093226 | 0.028306 |
| 279 | M phase | 383 | 77 | 4.7853941 | 0.028702 |
| 6261 | DNA-dependent DNA replication | 74 | 19 | 4.717244 | 0.029862 |
| 70 | mitotic sister chromatid segregation | 37 | 11 | 4.6594102 | 0.030884 |
| 819 | sister chromatid segregation | 37 | 11 | 4.6594102 | 0.030884 |
| 19538 | protein metabolism | 2606 | 452 | 4.6142123 | 0.031708 |
| 45451 | pole plasm oskar mRNA localization | 51 | 14 | 4.5082434 | 0.033732 |
| 8033 | tRNA processing | 33 | 10 | 4.5017172 | 0.033861 |
| 2217 | physiological defense response | 120 | 28 | 4.4849355 | 0.034195 |
| 9719 | response to endogenous stimulus | 167 | 37 | 4.4688338 | 0.034519 |
| 43037 | Translation | 226 | 48 | 4.4568944 | 0.034761 |
| 7622 | rhythmic behavior | 42 | 12 | 4.4530063 | 0.03484 |
| 45941 | positive regulation of transcription | 105 | 25 | 4.4277477 | 0.035359 |
| 7059 | chromosome segregation | 162 | 36 | 4.4245519 | 0.035425 |
| 6807 | nitrogen compound metabolism | 506 | 98 | 4.3923723 | 0.0361 |
| 30261 | chromosome condensation | 29 | 9 | 4.3616769 | 0.036756 |
| 6873 | cell ion homeostasis | 38 | 11 | 4.2672897 | 0.038853 |
| 30003 | cation homeostasis | 38 | 11 | 4.2672897 | 0.038853 |
| 6961 | antibacterial humoral response (sensu Protostomia) | 25 | 8 | 4.2477624 | 0.039302 |
| 45935 | positive regulation of nucleobase, nucleoside, nucleotide and nucleic acid metabolism | 106 | 25 | 4.2263681 | 0.039801 |
| 6865 | amino acid transport | 52 | 14 | 4.1936387 | 0.040576 |
| 15837 | amine transport | 52 | 14 | 4.1936387 | 0.040576 |
| 6277 | DNA amplification | 17 | 6 | 4.1720784 | 0.041095 |
| 15074 | DNA integration | 17 | 6 | 4.1720784 | 0.041095 |
| 30717 | karyosome formation | 17 | 6 | 4.1720784 | 0.041095 |
| 45471 | response to ethanol | 17 | 6 | 4.1720784 | 0.041095 |
| 50830 | defense response to Gram-positive bacterium | 17 | 6 | 4.1720784 | 0.041095 |
| 6260 | DNA replication | 143 | 32 | 4.0933252 | 0.043053 |
| 45944 | positive regulation of transcription from RNA polymerase II promoter | 34 | 10 | 4.090036 | 0.043137 |
| 48111 | oocyte axis determination (sensu Insecta) | 77 | 19 | 3.9753169 | 0.046172 |
| 31324 | negative regulation of cellular metabolism | 203 | 43 | 3.9221297 | 0.047654 |
| 375 | RNA splicing, via transesterification reactions | 198 | 42 | 3.8671408 | 0.04924 |
| 377 | RNA splicing, via transesterification reactions with bulged adenosine as nucleophile | 198 | 42 | 3.8671408 | 0.04924 |
| 398 | nuclear mRNA splicing, via spliceosome | 198 | 42 | 3.8671408 | 0.04924 |
| 7049 | cell cycle | 605 | 114 | 3.8643046 | 0.049324 |
| 7582 | physiological process | 8256 | 1365 | 3.8493338 | 0.049766 |
