# Supplementary material for: Quantitative and Molecular Genetic Analyses of Mutations Increasing Drosophila Life Span
Source: PLoS Genet. 2010 Jul 29;6(7):e1001037. doi: 10.1371/journal.pgen.1001037 (PMC2912381; doi:10.1371/journal.pgen.1001037)
Supplement: Table S10 — Candidate genes on the array. (0.20 MB DOC) [file pgen.1001037.s014.doc]

**Table S10**

**Candidate life span genes on the array**

| **Reason** | **Candidate gene name** | **Gene symbol** | **Probe sets** | **Genotype *P*-value** | **Sex *P*-value** | **Genotype × Sex *P*-value** |
| --- | --- | --- | --- | --- | --- | --- |
| **Lines on Microarray** | *polychaetoid* | *pyd* | 1627360_at | 0.00371 | 0.153104 | 2.15E-10 |
|  |  | *pyd* | 1631905_a_at | 0.32705 | 0.380906 | 0.118432 |
|  |  | *pyd* | 1630791_a_at | 0.39715 | 0.592731 | 0.033877 |
|  |  | *pyd* | 1637322_at | 0.00059 | 0.174538 | 8.53E-08 |
|  |  | *pyd* | 1637428_a_at | 0.00021 | 0.008277 | 2.13E-10 |
|  | *mushroom-body expressed* | *mub* | 1627456_s_at | 0.00048 | 0.06327 | 0.00026 |
|  | *crooked legs* | *crol* | 1626018_a_at | 0.08785 | 0.008821 | 3.81E-10 |
|  | *CG10990* | *CG10990* | 1640020_at | 0.00000 | 0.006239 | 3.12E-10 |
|  | *CG9238* | *CG9238* | 1632222_a_at | 0.00002 | 0.222866 | 1.51E-05 |
|  | BG00817 | BG00817 | Unknown |  |  |  |
|  | *escargot* | *esg* | 1641639_at | 0.00001 | 0.003489 | 1.60E-07 |
| **Screen Increased** | *CG31531* | *CG31531* | 1623254_s_at | 0.00016 | 0.000115 | 2.39E-14 |
|  | *Trapped in endoderm-1* | *Tre1* | 1625691_at | 0.00323 | 0.001547 | 3.21E-17 |
|  | *Gustatory receptor 5a* | *Gr5a* | No expression |  |  |  |
|  | *CG18418* | *CG18418* | 1627208_at | 0.00075 | 0.001425 | 1.71E-14 |
|  | *Guanine nucleotide exchange factor GEF64C* | *Gef64C* | 1623087_at | 0.66474 | 0.93185 | 0.001093 |
|  | *Osiris 9* | *Osi9* | No expression |  |  |  |
|  | *Tetraspanin 42Ef* | *Tsp42Ef* | 1634845_at | 0.38113 | 0.595013 | 0.694978 |
|  | *CG13334* | *CG13334* | No expression |  |  |  |
|  | *CG13503* | *CG13503* | 1634562_s_at | 0.17543 | 0.49916 | 6.13E-08 |
|  | *meiotic from via Salaria 332* | *mei-S332* | 1639029_at | 0.00119 | 0.006952 | 2.51E-14 |
|  | *pipsqueak* | *psq* | 1639136_s_at | 0.14164 | 0.080615 | 8.37E-14 |
|  | *CG14696* | *CG14696* | 1635764_at | 0.50798 | 0.884622 | 2.92E-07 |
|  | *CG4674* | *CG4674* | 1627043_at | 0.13420 | 0.170006 | 8.27E-12 |
|  | *CG32679* | *CG32679* | No expression |  |  |  |
|  | *Ecdysone-induced protein 75B* | *Eip75B* | 1635393_s_at | 0.35954 | 0.075864 | 0.19305 |
|  |  | *Eip75B* | 1640781_a_at | 0.04023 | 0.079918 | 3.72E-09 |
|  | *scalloped* | *sd* | 1625515_a_at | 0.03109 | 0.571102 | 0.227793 |
|  | *kayak* | *kay* | 1640886_a_at | 0.00136 | 0.019123 | 2.00E-09 |
|  | *CG13130* | *CG13130* | No expression |  |  |  |
|  | *big brain* | *bib* | 1634044_at | 0.10398 | 0.332684 | 9.98E-05 |
|  | *foxhead box, sub-group O* | *foxo* | 1631597_at | 0.24744 | 0.010422 | 0.800791 |
|  | *nervana 3* | *nrv3* | 1640729_s_at | 0.15210 | 0.658699 | 1.91E-11 |
|  | *CG17841* | *CG17841* | 1623704_at | 0.03622 | 0.07664 | 5.02E-12 |
|  | *CG4630* | *CG4630* | 1633536_at | 0.09748 | 0.037183 | 5.85E-13 |
|  | *CG4646* | *CG4646* | 1638233_at | 0.03458 | 0.024135 | 2.54E-10 |
|  | *Dek* | *Dek* | 1628041_a_at | 0.00128 | 0.006991 | 7.32E-18 |
|  | *Glucose transporter 1* | *Glut1* | 1634033_s_at | 0.13956 | 0.076951 | 1.36E-10 |
|  |  | *Glut1* | 1628757_at | 0.75927 | 0.800139 | 0.051221 |
|  | *Phosphogycerate kinase* | *Pgk* | 1639766_at | 0.15942 | 0.03507 | 0.003108 |
|  | *CG9171* | *CG9171* | 1633801_s_at | 0.08264 | 0.420647 | 0.000134 |
|  | *Defense repressor 1* | *Dnr1* | 1626336_at | 0.11620 | 0.243392 | 7.55E-11 |
|  | *CG32604* | *CG32604* | Not on array |  |  |  |
|  | *Fkbp13* | *Fkbp13* | 1633836_a_at | 0.02725 | 0.383334 | 1.73E-07 |
|  |  |  |  |  |  |  |
| **Screen Decreased** | *6-phosphofructo-2-kinase* | *Pfrx* | 1633454_s_at | 0.04065 | 0.421154 | 6.91E-10 |
|  | *Actin 5C* | *Act5c* | 1626163_s_at | 0.01550 | 0.327268 | 0.007675 |
|  | *Adh-related* | *Adhr* | Not on array |  |  |  |
|  | *outspread* | *osp* | 1627054_at | 0.44994 | 0.134978 | 1.82E-09 |
|  | *β4GalNAcTA* | *β4GalNAcTA* | 1638113_at | 0.31412 | 0.10037 | 2.75E-10 |
|  | *Basigin* | *Bsg* | 1628276_s_at | 0.04587 | 0.433909 | 3.48E-07 |
|  |  | *Bsg* | 1640457_s_at | 0.11869 | 0.504995 | 0.941386 |
|  | *CG13334* | *CG13334* | No expression |  |  |  |
|  | *CG13333* | *CG13333* | No expression |  |  |  |
|  | *CG14478* | *CG14478* | 1638359_s_at | 0.00009 | 0.084546 | 7.27E-13 |
|  | *CG14709* | *CG14709* | 1637439_at | 0.92822 | 0.553751 | 3.52E-05 |
|  | *CG31176* | *CG31176* | 1637788_at | 0.00055 | 0.044339 | 1.25E-07 |
|  | *CG32541* | *CG32541* | 1640125_s_at | 0.19032 | 0.24999 | 6.15E-09 |
|  |  | *CG32541* | 1639917_at | 0.32928 | 0.327388 | 3.90E-12 |
|  | *CG3777* | *CG3777* | 1639917_at | 0.32928 | 0.327388 | 3.90E-12 |
|  |  | *CG3777* | 1637077_s_at | 0.00071 | 8.48E-05 | 8.25E-09 |
|  | *CG4004* | *CG4004* | 1638751_a_at | 0.00000 | 0.001401 | 5.07E-11 |
|  | *CG6854* | *CG6854* | 1639986_at | 0.00000 | 4.33E-10 | 7.23E-16 |
|  |  | *CG6854* | 1640191_a_at | 0.01321 | 0.056292 | 2.34E-11 |
|  | *CG9650* | *CG9650* | 1625641_s_at | 0.09188 | 0.224863 | 0.000946 |
|  |  | *CG9650* | 1639550_a_at | 0.00006 | 0.385491 | 0.890127 |
|  | *CG9894* | *CG9894* | 1628814_s_at | 0.30507 | 0.884755 | 0.001577 |
|  | *chameau* | *Chm* | 1631863_at | 0.03701 | 0.069558 | 1.16E-15 |
|  | *couch potato* | *Cpo* | 1632644_s_at | 0.00005 | 0.001899 | 5.35E-12 |
|  |  | *Cpo* | 1624608_s_at | 0.02512 | 0.104114 | 5.62E-09 |
|  | *dacapo* | *Dap* | 1625199_s_at | 0.08879 | 0.121556 | 2.04E-13 |
|  | *division abnormally delayed* | *Dally* | 1628489_at | 0.05406 | 0.598904 | 1.47E-05 |
|  | *Defense repressor 1* | *Dnr1* | 1626336_at | 0.11620 | 0.243392 | 7.55E-11 |
|  | *escargot* | *Esg* | 1641639_at | 0.00001 | 0.003489 | 1.60E-07 |
|  | *failed axon connections* | *Fax* | 1624705_a_at | 0.46868 | 0.174456 | 4.36E-07 |
|  | *GDP dissociation inhibitor* | *Gdi* | 1641129_at | 0.70223 | 0.07848 | 1.72E-07 |
|  | *Gliotactin* | *Gli* | 1624203_s_at | 0.18260 | 0.254794 | 1.54E-08 |
|  | *grapes* | *Grp* | 1629880_at | 0.48372 | 0.519405 | 0.477172 |
|  |  | *Grp* | 1634230_s_at | 0.03433 | 0.089501 | 1.90E-18 |
|  |  | *Grp* | 1625416_x_at | 0.20469 | 0.378622 | 0.029202 |
|  | *inaF* | *inaF* | 1633646_at | 0.00001 | 0.01086 | 2.16E-12 |
|  | *innexin 7* | *inx7* | 1638225_a_at | 0.01022 | 0.033513 | 0.01024 |
|  | *ken and barbie* | *Ken* | 1628840_at | 0.00369 | 0.003084 | 1.02E-16 |
|  | *Laminin A* | *LanA* | 1641324_at | 0.00000 | 0.211264 | 0.003946 |
|  | *Lipid storage droplet-2* | *Lsd-2* | 1637359_at | 0.00007 | 0.02482 | 4.60E-13 |
|  | *longitudinals lacking* | *Lola* | 1623411_at | 0.03613 | 0.064539 | 8.80E-11 |
|  |  | *Lola* | 1634495_s_at | 0.03664 | 0.672669 | 7.56E-08 |
|  |  | *Lola* | 1627324_at | 0.04434 | 0.00672 | 4.73E-14 |
|  |  | *Lola* | 1637581_at | 0.04663 | 0.074247 | 3.57E-11 |
|  |  | *Lola* | 1625768_s_at | 0.12377 | 0.106139 | 1.58E-16 |
|  |  | *Lola* | 1641609_at | 0.13149 | 0.694246 | 1.45E-05 |
|  |  | *Lola* | 1629523_at | 0.19323 | 0.207933 | 0.703383 |
|  |  | *Lola* | 1640945_at | 0.20505 | 0.132804 | 0.014554 |
|  |  | *Lola* | 1633089_a_at | 0.22648 | 0.838396 | 4.12E-07 |
|  |  | *Lola* | 1630936_at | 0.55668 | 0.727902 | 0.018777 |
|  |  | *Lola* | 1624729_at | 0.01158 | 0.251913 | 1.38E-06 |
|  |  | *Lola* | 1633422_a_at | 0.01160 | 0.314343 | 1.03E-09 |
|  |  | *Lola* | 1628421_at | 0.01769 | 0.117611 | 6.07E-11 |
|  |  | *Lola* | 1640280_at | 0.02480 | 0.016341 | 1.98E-09 |
|  | *Malic enzyme* | *Men* | 1629095_a_at | 0.00003 | 0.008492 | 3.49E-15 |
|  | *mushroom-body expressed* | *Mub* | 1627456_s_at | 0.00048 | 0.06327 | 0.00026 |
|  | *neuralized* | *Neur* | 1638682_a_at | 0.79723 | 0.093373 | 1.06E-10 |
|  | *plexus* | *Px* | 1638412_at | 0.12987 | 0.023615 | 0.008999 |
|  | *polychaetoid* | *Pyd* | 1627360_at | 0.00371 | 0.153104 | 2.15E-10 |
|  |  | *Pyd* | 1631905_a_at | 0.32705 | 0.380906 | 0.118432 |
|  |  | *Pyd* | 1630791_a_at | 0.39715 | 0.592731 | 0.033877 |
|  |  | *Pyd* | 1637322_at | 0.00059 | 0.174538 | 8.53E-08 |
|  |  | *Pyd* | 1637428_a_at | 0.00021 | 0.008277 | 2.13E-10 |
|  | *Protein kinase 61C* | *Pk61C* | 1629078_s_at | 0.00001 | 0.205837 | 0.000459 |
|  | *Protein tyrosine phosphatase 10D* | *Ptp10D* | 1624660_at | 0.58279 | 0.440217 | 1.94E-09 |
|  |  | *Ptp10D* | 1634653_a_at | 0.04158 | 0.345119 | 2.15E-08 |
|  | *Rab23* | *Rab23* | 1635643_at | 0.00163 | 0.001611 | 1.54E-17 |
|  | *rutabaga* | *Rut* | 1628594_at | 0.28583 | 0.022281 | 3.28E-05 |
|  | *Sema-1a* | *Sema-1a* | 1634588_at | 0.43898 | 0.163681 | 7.17E-07 |
|  | *serrano* | *Sano* | 1627971_s_at | 0.25419 | 0.710066 | 1.47E-11 |
|  | *singed* | *Sn* | 1622932_s_at | 0.00002 | 0.026886 | 2.87E-12 |
|  | *Tob* | *Tob* | 1628358_at | 0.00714 | 0.026936 | 0.000435 |
|  | *Vacuolar H+ ATPase 16kD subunit* | *Vha16* | 1632117_s_at | 0.05983 | 0.382292 | 0.002188 |
|  |  |  |  |  |  |  |
| **Known life span genes** | *I'm not dead yet* | *Indy* | 1641566_s_at | 0.00108 | 0.343714 | 8.12E-06 |
|  | *chico* | *Chico* | 1631374_at | 0.00223 | 0.167928 | 1.01E-05 |
|  | *Insulin-like receptor* | *InR* | 1629141_at | 0.00000 | 0.02448 | 0.080178 |
|  | *Superoxide dismutase* | *Sod* | 1630845_at | 0.00047 | 0.029841 | 2.99E-08 |
|  | *Superoxide dismutase 2 (Mn)* | *Sod2* | 1622984_at | 0.06031 | 0.269416 | 0.002475 |
|  | *Alcohol dehydrogenase* | *Adh* | 1632699_s_at | 0.00907 | 0.367264 | 1.35E-10 |
|  |  | *Adh* | 1631333_s_at | 0.00008 | 0.015023 | 0.020979 |
|  | *shuttle craft* | *Stc* | 1640237_a_at | 0.00716 | 0.072542 | 7.62E-13 |
|  | *Dopa decarboxylase* | *Ddc* | 1639320_a_at | 0.58643 | 0.643968 | 4.64E-11 |
|  | *Target of rapamycin* | *Tor* | 1641510_at | 0.08786 | 0.27943 | 2.32E-09 |
|  | *Catecholamines up* | *Catsup* | 1627458_at | 0.12968 | 0.241096 | 1.57E-09 |
|  | *Glutathione S transferase S1* | *GstS1* | 1623957_s_at | 0.00369 | 0.006176 | 1.36E-16 |
|  | *Sirt2* | *Sirt2* | 1626458_at | 0.00013 | 0.006002 | 2.11E-10 |
|  | *methuselah* | *Mth* | 1632989_a_at | 0.03618 | 0.551107 | 0.155524 |
|  | *Pten* | *Pten* | 1628114_s_at | 0.13704 | 0.016861 | 2.89E-13 |
|  |  | *Pten* | 1630738_s_at | 0.90044 | 0.12625 | 4.30E-06 |
|  | *Akt1* | *Akt1* | 1639064_s_at | 0.01945 | 0.173143 | 1.34E-09 |
|  | *Vacuolar H^+^-ATPase SFD subunit* | *VhaSFD* | 1633931_a_at | 0.00037 | 0.227853 | 1.54E-06 |
|  | *sugarbabe* | *Sug* | 1624195_at | 0.01664 | 0.350361 | 9.81E-07 |
|  | *cheerio* | *Cher* | 1632339_s_at | 0.13092 | 0.114286 | 0.385455 |
|  | *four wheel drive* | *Fwd* | 1634509_s_at | 0.44338 | 0.222732 | 0.008595 |
|  | *CTP:phosphocholine cytidylyltransferase 1* | *Cctl* | 1629930_s_at | 0.00107 | 0.05407 | 2.96E-13 |
|  | *Red-herring* | *Rdh* | Not on array |  |  |  |
